# Global and regional causes of maternal deaths 2009–20: a WHO systematic analysis

**DOI:** 10.1016/S2214-109X(24)00560-6

**Published:** 2025-03-08

**Authors:** Jenny A Cresswell, Monica Alexander, Michael Y C Chong, Heather M Link, Marija Pejchinovska, Ursula Gazeley, Sahar M A Ahmed, Doris Chou, Ann-Beth Moller, Daniel Simpson, Leontine Alkema, Gemma Villanueva, Yanina Sguassero, Özge Tunçalp, Qian Long, Shaoming Xiao, Lale Say

**Affiliations:** a UNDP–UNFPA–UNICEF–WHO–World Bank Special Programme of Research, Development, and Research Training in Human Reproduction (HRP), Department of Sexual and Reproductive Health and Research, WHO, Geneva, Switzerland; b Department of Statistical Sciences, University of Toronto, ON, Canada; c Department of Sociology, University of Toronto, ON, Canada; d Kaleida Health, Division of Maternal Fetal Medicine, Buffalo, NY, USA; e Department of Infectious Disease Epidemiology and International Health, London School of Hygiene & Tropical Medicine, London, UK; f Department of Econometrics and Business Statistics, Monash University, Melbourne, VIC, Australia; g Department of Biostatistics and Epidemiology, University of Massachusetts Amherst, Amherst, MA, USA; h Cochrane Response, Cochrane, London, UK; i Global Health Research Center, Duke Kunshan University, Kunshan, China; j School of Health Humanities, Peking University, Beijing, China

## Abstract

**Background:**

Maternal mortality is not on track to meet Sustainable Development Goal (SDG) target 3.1 of a global maternal mortality ratio below 70 per 100 000 livebirths by 2030. Updated evidence on causes of death is needed to accelerate progress.

**Methods:**

We conducted a multi-strategy systematic review to identify causes of maternal deaths occurring in 2009–20. Data sources included civil registration and vital statistics systems data from the WHO Mortality Database, reports published by Member States, and national and subnational journal articles identified via bibliographic databases. We used a Bayesian hierarchical model to estimate the maternal cause of death distribution by SDG regions and worldwide. Given the paucity of data on maternal suicide and late maternal deaths occurring beyond 42 days postpartum, additional analyses were conducted to estimate the proportion of maternal deaths from suicide and the ratio of maternal to late maternal deaths (all cause).

**Findings:**

Globally, the most common cause of maternal death was haemorrhage (27%; 80% uncertainty interval 22–32), followed by indirect obstetric deaths (23%, 18–30), and hypertensive disorders (16%, 14–19). The proportion of haemorrhage deaths varied substantially by region and was highest in sub-Saharan Africa and Western Asia and Northern Africa. The proportion of maternal deaths from hypertensive disorders was highest in Latin America and the Caribbean. Most maternal deaths from haemorrhage and sepsis occurred during the postpartum period. Only 12 countries recorded one or more maternal suicides; of those countries, the proportion of deaths from suicide ranged from below 1% to 26% of maternal deaths. For countries reporting at least one late maternal death (ie, deaths that occur more than 42 days but less than 1 year after the termination of pregnancy), the ratio of late maternal deaths to maternal deaths up to 42 days ranged from <0·01 to 0·07.

**Interpretation:**

Haemorrhage remains the leading cause of death, despite the existence of effective clinical interventions, emphasising the need for improved access to quality health care. The timing of most deaths in the postpartum period demands renewed commitment to improving the provision of postpartum care in addition to intrapartum care. Indirect causes of death require health system approaches to integrate obstetric and non-obstetric care.

**Funding:**

USAID; US Fund for UNICEF via the Bill & Melinda Gates Foundation; and UNDP–UNFPA–UNICEF–WHO–World Bank Special Programme of Research, Development, and Research Training in Human Reproduction (HRP).

## Introduction

A maternal death is defined by WHO as the “death of a woman while pregnant or within 42 days of termination of pregnancy, irrespective of the duration and the site of the pregnancy, from any cause related to or aggravated by pregnancy or its management, but not from accidental or incidental causes”.^1^ The UN Maternal Mortality Estimation Interagency Group (MMEIG) estimated that globally in 2020 approximately 287 000 (80% uncertainty interval [UI] 273 000–343 000) maternal deaths occurred.^2^ Globally in 2020, a 15-year-old girl had a one in 210 chance of dying from a maternal cause, almost half of the lifetime risk of maternal death in 2000, which was one in 116.^2^

Advances in maternal survival achieved between 2000 and 2015 stagnated in the first 5 years of the Sustainable Development Goal (SDG) era.^2^ In 2020, the global maternal mortality ratio (MMR) was 223 maternal deaths per 100 000 livebirths; progress is not on track to meet SDG target 3.1 of a global MMR of less than 70 per 100 000 livebirths by 2030.^2^ Global trends obscure substantial inequities in maternal survival as 95% of maternal deaths occur in low-income and lower-middle-income countries (LMICs) or fragile settings, and are preventable.^2^ Understanding the causes of maternal mortality is crucial to designing health system approaches across the continuum of care to end preventable maternal mortality.^3^

**Research in context**

**Evidence before this study**

Two previous WHO estimates of global causes of maternal mortality by Millenium Development Goal regions were published in 2006 and 2014. In 2006, haemorrhage was the leading cause of death in Africa and Asia, hypertensive disorders in Latin America and the Caribbean, and other direct causes in (what were then classified as) developed countries. In 2014, haemorrhage was the leading cause in all Millenium Development Goal regions except sub-Saharan Africa and developed regions, where indirect causes were highest. Widespread transitions in epidemiology, obstetric care utilisation and coverage, and health systems have occurred in the last two decades. We combined data from civil registration and vital statistics systems, government reports, and bibliographic database sources from 129 countries, including 139 381 total maternal deaths from 2009 to 2020, to update the previous WHO systematic reviews.

**Added value of this study**

To our knowledge, this Article provides the first update in a decade to the global causes of maternal mortality. Consistent with previous publications, haemorrhage remains the largest cause of maternal deaths globally, followed by indirect causes. Hypertensive disorders of pregnancy were the leading cause of maternal death in Latin America and the Caribbean. Deaths from haemorrhage disproportionately affect low-income and middle-income countries (LMICs), indicating persistent inequities in access to, and the quality of, emergency obstetric care. Most deaths from haemorrhage and sepsis occur in the postpartum period. To our knowledge, this Article is the first to report on suicide. Only 12 countries reported at least one maternal suicide; the mean proportion of deaths from suicide varied substantially between Sustainable Development Goal regions (from <1% in sub-Saharan Africa to 26% in Australia and New Zealand).

**Implications of all the available evidence**

Decisive action is needed to address mortality and morbidity from haemorrhage in LMICs. The WHO Roadmap to Combat Postpartum Haemorrhage 2023 to 2030 provides strategies for research, norms and standards, implementation, and advocacy to accelerate progress on haemorrhage. The high proportion of deaths occurring in the postpartum period underscores the need for renewed commitment to improve early routine postpartum care. In Latin America and the Caribbean in particular, the considerable contribution of hypertensive disorders emphasises the need to improve preventive interventions, in addition to early diagnosis and identification of severe disease. Finally, the substantial contribution of indirect maternal deaths emphasises the importance of a health systems approach to integrate obstetric and non-obstetric care providers across the continuum of care.

Previous WHO analyses, published in 2006 and 2014, described the distribution of causes of maternal death.^4^, ^5^ Since 2014, the distribution of maternal causes of death might have changed due to transitions in proximal and distal determinants of maternal mortality, which influence the health of women before, during, and after pregnancy.^6^ The strengthening of health systems has led to substantial improvements in the coverage and use of obstetric care, including antenatal care and births attended by skilled health personnel.^7^ These improvements have occurred alongside widespread transitions in epidemiology. HIV/AIDS-related mortality has decreased by 60% since its peak in 2004, and by 39% since 2010.^8^ The burden of non-communicable diseases remains substantial.^9^

The aim of this systematic analysis was to develop estimates of the causes of maternal deaths at global and regional levels for the period from 2009 to 2020. Specifically, we estimate the proportion of maternal deaths according to seven groups: abortion, embolism, haemorrhage, hypertensive disorders, pregnancy-related sepsis, other direct causes, and indirect causes. We present data reporting maternal suicides, when available, as a secondary objective.

## Methods

### Search strategy and selection criteria

For this systematic analysis, we collected data in two stages: search 1 covered 2009–17 and search 2 covered 2017–20. In search 1, studies were eligible if the midpoint of the reference period (ie, time period in which maternal deaths were recorded) was 2009 onwards, where non-disaggregated pre-2009 data were present. In search 2, pre-2017 data were eligible if they were published after search 1 and the midpoint of the reference period was 2017 onwards.

We included national and subnational studies representative at country administrative level 1 and higher. Single-facility studies were excluded. Studies were eligible if they reported a usable denominator (ie, livebirths, total births, pregnancies, deliveries, obstetric admissions, women of reproductive age, or total maternal deaths). Studies reporting on sub-populations only were excluded (eg, caesarean deliveries only).

Data were identified via three main pathways: the WHO Mortality Database, reports published by Member States (ie, the MMEIG Database), and journal articles identified via bibliographic databases.

Civil registration and vital statistics systems (CRVS) data available in the WHO Mortality Database as of Feb 21, 2024, were included. Where a country had both CRVS data and high-quality national data from an in-depth investigation into maternal mortality, such as a confidential enquiry, the latter were preferred.

The MMEIG Database contains data reported by Member States used to calculate the MMEIG maternal mortality estimates.^2^ In addition, a search of the websites of the Ministry of Health and National Statistics Office of each Member State was conducted on July 24, 2019.

For the bibliographic searches, we used search terms adapted from earlier WHO maternal cause of death searches (appendix p 6) from database inception until April 30, 2019.^4^, ^5^

For search 1, searches were separated by script: search 1A covered Latin-script databases (ie, MEDLINE, Embase, Global Index Medicus, Web of Science, and Popline); search 1B covered Chinese-script databases (ie, Wanfang and CNKI); search 1C used a Russian-script database (eLIBRARY.RU). The databases for Russian and Chinese scripts were included as these are the predominant languages for scientific publishing in some world regions. For search 1A, two reviewers screened for eligibility and a third reviewer (GV, YS, or JAC) adjudicated if conflicts arose. Due to capacity constraints, a single reviewer screened Chinese-script (SX) and Russian-script publications, who discussed and consulted with a third reviewer (GV for Russian, JAC for Chinese).

In search 2, only search 1A was repeated due to the low yield of search 1B and search 1C. We used an artificial intelligence classifier model (Risklick Artificial Intelligence; University of Bern, Bern, Switzerland) that ranked citations according to relevance to improve search efficiency (appendix p 45).^10^ Two reviewers (SMAA and UG) screened for eligibility. Double-reviewer screening was piloted for the first 1000 sources with 99% agreement; sources were single-reviewer screened thereafter.

### Data extraction and classification of maternal deaths

For manuscripts in which the cause of maternal death was published in free-text format, the underlying cause of death was assigned an ICD-10 code by an obstetrician (HML; appendix pp 46–50).^1^ We used the ICD 10th edition definition of maternal mortality: “the death of a woman while pregnant or within 42 days of termination of pregnancy, irrespective of the duration and the site of the pregnancy, from any cause related to or aggravated by the pregnancy or its management, but not from accidental or incidental causes”.^1^

We grouped maternal causes of death into the categories described by Say and colleagues:^5^ abortion, embolism, haemorrhage, hypertensive disorders, pregnancy-related sepsis, other direct causes, and indirect causes. Indirect causes include pre-existing hypertensive disorders complicating pregnancy, childbirth, and the puerperium; diabetes in pregnancy, childbirth, and the puerperium; other maternal infectious and parasitic diseases complicating pregnancy, childbirth, and the puerperium; and other maternal diseases classifiable elsewhere. Haemorrhage and sepsis deaths are disaggregated by timing (ie, antepartum, intrapartum, or postpartum). Other direct maternal deaths are disaggregated into complications of anaesthesia, obstructed labour, obstetric trauma, and other. The ICD-10 codes in each category are available in the appendix (pp 46–50).

Within the maternal mortality section of the ICD, deaths by suicide occurring temporal to pregnancy, childbirth, and the puerperium are considered direct maternal deaths,^1^ but are not included in the MMEIG maternal mortality estimates due to considerable international variation in data availability. Consequently, we report deaths by suicide separately.

### Statistical analysis

We estimated maternal cause of death distributions using a Bayesian hierarchical model; further details, including model evaluation and validation, are available in the appendix (pp 51–56).^11^ The model estimates the cause of death distribution per country-year of observation for each of the categories described above. Country-year estimates are then aggregated to SDG region (appendix pp 57, 58). The MMEIG maternal mortality estimates are used to convert cause of death distributions into the number of maternal deaths by cause.^2^

Due to substantial variability in the HIV/AIDS epidemic internationally, the model estimates non-HIV/AIDS deaths only.^2^, ^12^ Estimates of HIV/AIDS-related indirect maternal deaths from the MMEIG Bayesian maternal mortality model were incorporated into the final distributions presented.^2^, ^12^

The Bayesian hierarchical model enables estimation even when data are sparse. Country estimates are informed by patterns in their region, and regions with scarce information by patterns elsewhere. The model accounts for correlation between causes that commonly co-occur. The model assigns varying amounts of uncertainty in the data based on data source: high-quality national data from in-depth investigations were assumed to have the least uncertainty, whereas subnational studies were assumed to have the most uncertainty. The model also accounts for additional uncertainty in studies where only a subset of the maternal deaths had a cause assigned.

We used a Markov Chain Monte Carlo algorithm to generate samples of the posterior distributions of all parameters in the model, including country-year specific cause of death distributions. This was implemented in Stan using the ‘cmdstanr’ package in the statistical software R. Point estimates for proportions were given by the posterior medians of the proportion and 80% UIs were given by the 10th and 90th percentiles of the posterior distributions. UIs were not estimated for suicides because these derive from observed data and were not modelled.

We report the number of countries reporting maternal suicide, and the average proportion of maternal deaths due to suicide for countries that report at least one such death. However, since suicide ICD codes in CRVS data do not report pregnancy status, the data we report on maternal suicide derive only from specialised reporting.

For countries with available data, we report the average ratio of late maternal deaths to maternal deaths up to 42 days after the end of pregnancy. We also report on late maternal deaths, which occur more than 42 days but less than 1 year after the termination of pregnancy.^1^

This study is a secondary analysis of publicly available data, and thus no ethical approval was obtained.

The protocol was registered on PROSPERO (CRD42019121340) and the GATHER checklist is available in the appendix (pp 4,5).

### Role of the funding source

The funders of the study had no role in study design, data collection, data analysis, data interpretation, or writing of the report.

## Results

**Figure 1:**
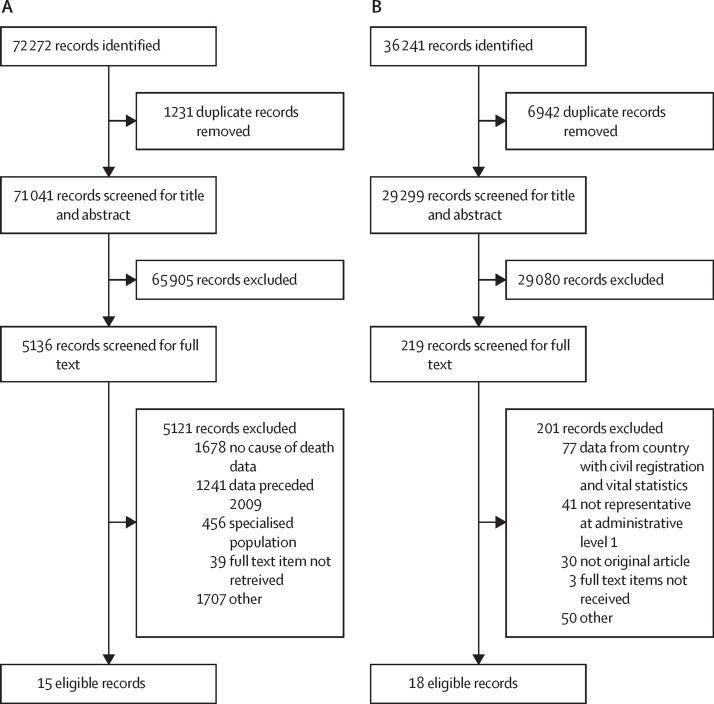
Search strategy and selection criteria (A) Search 1 is for 2009–17 and was conducted in 2019. (B) Search 2 is for 2017–20 and was conducted in 2023. Detailed PRISMA diagrams for all searches are available in the appendix (pp 36–44).

Search 1 identified 74 272 citations; 5136 citations were screened for full text; and 15 citations were eligible (figure 1; appendix pp 36–38). Search 2 identified 36 241 citations via the search of the bibliographic databases; 219 citations were reviewed for full text; and 18 sources were eligible (figure 1; appendix p 44).

We included CRVS data from 110 countries, covering 908 country-years; government reports from 28 countries covering 79 country-years; and data originating from bibliographic databases from 20 countries covering 33 country-years (figure 1). In total, we included data from 129 countries, used to generate estimates for 185 countries and 972 country-years. The 56 countries for which no data are available are listed in the appendix (pp 59–62). The total number of maternal deaths in the final input dataset in 2009–20 was 139 381, which represents 3·7% of the 3 813 266 maternal deaths estimated by the MMEIG over this period globally.

**Figure 2:**
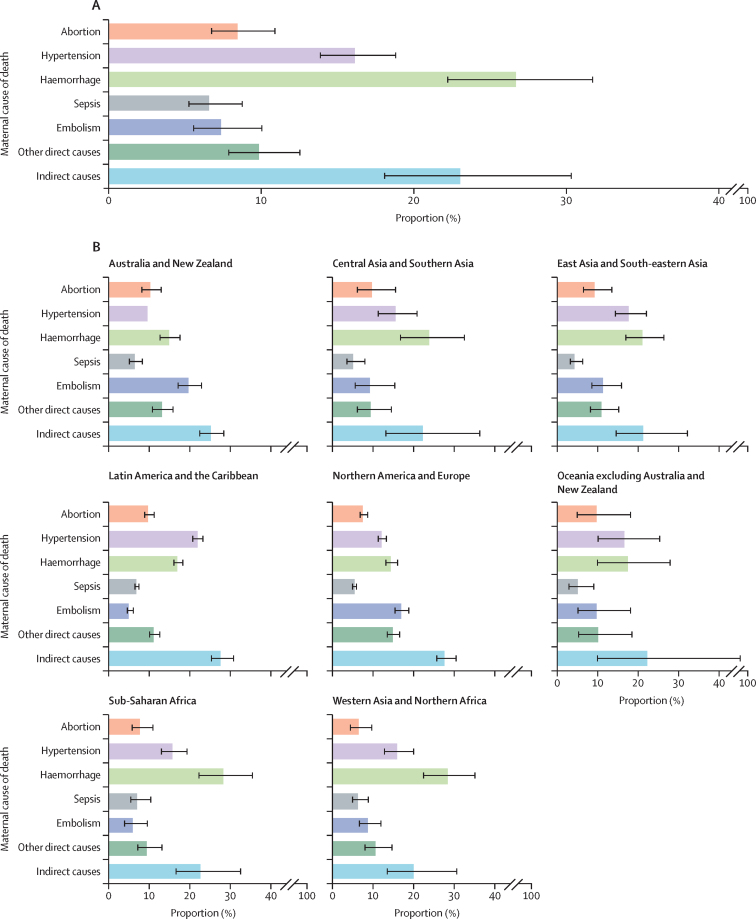
Distribution of causes of maternal deaths (A) Global distribution of causes of maternal deaths. (B) Regional distribution of causes of maternal deaths for Sustainable Development Goal regions. Bars indicate median values with 80% uncertainty intervals.

Figure 2 presents the global distribution of cause of maternal death by SDG region. Globally, the most common cause of maternal death between 2009 and 2020 was haemorrhage (27%, UI 22–32), followed by indirect obstetric deaths (23%, 18–30), and hypertensive disorders (16%, 14–19). Abortion represented 8% of maternal deaths (7–11), pregnancy-related infection 7% (5–9); embolism 7% (6–10); and other direct causes 10% (8–13; appendix pp 63, 64).

There was substantial regional variation in the proportion of maternal deaths due to haemorrhage. Haemorrhage caused 29% (UI 22–35) of maternal deaths in Western Asia and Northern Africa and 28% (22–36) of maternal deaths in sub-Saharan Africa. However, in Australia and New Zealand, haemorrhage caused 15% (13–18) of maternal deaths, and in Northern America and Europe, it caused 15% (13–16) of maternal deaths. As the proportion of deaths due to haemorrhage is generally higher in regions with a higher absolute burden of maternal deaths, the difference in number of deaths is substantial.

The Latin America and the Caribbean region was estimated to have approximately double the proportion of maternal deaths due to hypertension (22%, UI 21–23) than Australia and New Zealand (10%, 8–12) and Northern America and Europe (12%, 11–13). The proportion of deaths due to embolism was highest in Australia and New Zealand (20%, 17–23) and lowest in Latin America and the Caribbean (5%, 4–6).

Maternal deaths due to complications of anaesthesia were approximately 1% (UI 0·8–1·9) of maternal deaths globally (table 1). Maternal deaths due to obstructed labour were estimated to represent around 2% (1·4–2·9) of maternal deaths, with deaths due to obstetric trauma other than obstructed labour representing a further 2% (1·6–3·3).

**Table 1:** Distribution of other direct causes of maternal deaths, by Sustainable Development Goal regions

	**Complications of anaesthesia**		**Obstructed labour**		**Obstetric trauma**		**Other**		**All direct causes**	
	n	% (80% UI)	n	% (80% UI)	n	% (80% UI)	n	% (80% UI)	N	% (80% UI)
Global	45 694	1·2% (0·8–1·9)	74 144	1·9% (1·4–2·9)	86 546	2·2% (1·6–3·3)	160 786	4·2% (3·1–5·6)	379 002	9·8% (7·8–12·5)
Australia and New Zealand	5	1·9% (0·9–3·5)	5	2·1% (1·1–3·9)	7	2·9% (1·3–5·2)	14	5·5% (3·3–8·2)	33	13·1% (10·6–15·9)
Central Asia and Southern Asia	11 052	1·4% (0·7–2·8)	14 116	1·7% (0·8–3·6)	12 091	1·5% (0·7–3·1)	33 710	4·2% (2·3–7·1)	76 707	9·4% (6·0–14·6)
Eastern Asia and Southeastern Asia	2149	0·8% (0·4–1·5)	7104	2·6% (1·8–4·3)	6380	2·4% (1·4–4·2)	12 721	4·7% (3·2–7·2)	29 856	11·1% (8·1–15·4)
Latin America and the Caribbean	955	1·0% (0·8–1·5)	2736	2·8% (2·5–3·5)	940	1·0% (0·8–1·4)	5888	6·1% (5·4–7·1)	10 735	11·1% (9·9–12·7)
North America and Europe	142	0·9% (0·6–1·3)	163	1·0% (0·7–1·5)	247	1·5% (1·1–2·1)	1890	11·4% (10·2–12·7)	2470	14·9% (13·4–16·6)
Oceania*	52	0·7% (0·2–2·0)	131	1·8% (0·6–4·8)	180	2·5% (0·9–5·9)	299	4·1% (1·6–8·8)	749	10·3% (5·2–18·6)
Sub-Saharan Africa	27 586	1·1% (0·7–2·0)	44 484	1·8% (1·1–3·0)	60 295	2·4% (1·6–3·8)	95 813	3·8% (2·5–5·8)	237 864	9·4% (6·9–13·1)
Western Asia and Northern Africa	1458	1·2% (0·7–2·1)	2173	1·8% (1·0–3·2)	3025	2·5% (1·5–4·3)	5912	4·8% (3·3–7·0)	13 178	10·7% (7·9–14·8)

Data are estimated counts of cause of death and % with 80% UI. Causes of death that make up the “Other” category are listed in the appendix (pp 49, 50). Discrepancies are a consequence of how the estimates are generated in the Bayesian model. The output from the model provides samples of the estimates of interest (eg, the number of antepartum haemorrhage deaths), and the median of those samples is used to derive the point estimate. Therefore, the median number of deaths for each subgroup might not add to the total median all direct causes. UI=uncertainty interval.

* Including Australia and New Zealand.

Only 12 countries reported deaths due to maternal suicide (appendix p 65). Two countries in Australia and New Zealand (average proportion of deaths from suicide out of all maternal deaths 26%); two countries in Central Asia and Southern Asia (average proportion 3%); one country in Eastern Asia and South-Eastern Asia (21%); one country in Latin America and the Caribbean (9%); three countries in Northern America and Europe (6%); one country in sub-Saharan Africa (below 1%); and two countries in Western Asia and Northern Africa (4%).

Table 2 presents the breakdown of haemorrhage and pregnancy-related infection deaths by timing relative to the end of pregnancy. Most haemorrhage deaths occurred in the postpartum period (up to 42 days after the end of pregnancy) with the remainder approximately evenly distributed between the antepartum and intrapartum periods. Globally, most pregnancy-related infection deaths occurred postpartum.

**Table 2:** Subgroup analysis of deaths due to haemorrhage and sepsis by Sustainable Development Goal region

	**Antepartum**		**Intrapartum**		**Postpartum**		**Total**	
	n	% (80% UI)	n	% (80% UI)	n	% (80% UI)	N	% (80% UI)
**Haemorrhage**								
Global	240 173	6·2 (4·7–8·8)	192 538	5·0 (3·7–7·3)	571 940	14·8 (11·6–18·6)	1 028 470	26·7 (22·2–31·7)
Australia and New Zealand	11	4·3 (3·0–6·0)	8	3·4 (2·1–5·1)	17	7·0 (5·0–9·3)	37	14·9 (12·5–17·7)
Central Asia and Southern Asia	41 414	5·1 (2·9–8·8)	38 851	4·8 (2·7–8·3)	105 482	13·0 (8·0–19·3)	194 770	24·0 (16·8–32·6)
Eastern Asia and South-eastern Asia	13 529	5·0 (3·5–7·4)	8753	3·2 (2·2–5·0)	33 483	12·4 (9·4–16·5)	57 296	21·3 (17·0–26·6)
Latin America and the Caribbean	4203	4·3 (4·0–4·9)	2220	2·3 (2·0–2·7)	9897	10·2 (9·5–11·1)	16 435	17·0 (16·0–18·2)
Northern America and Europe	679	4·1 (3·6–4·9)	551	3·3 (2·8–4·1)	1136	6·9 (5·9–8·1)	2394	14·5 (13·0–16·2)
Oceania*	315	4·3 (1·8–8·8)	184	2·5 (1·0–5·5)	708	9·7 (4·4–17·3)	1287	17·6 (9·9–28·0)
Sub-Saharan Africa	167 286	6·6 (4·6–9·9)	132 220	5·2 (3·6–8·2)	394 825	15·6 (11·2–20·9)	717 502	28·4 (22·2–35·5)
Western Asia and Northern Africa	7860	6·4 (4·3–9·8)	5537	4·5 (3·0–7·2)	20 555	16·7 (12·3–21·9)	35 075	28·5 (22·4–35·2)
**Sepsis**								
Global	55 774	1·4 (1·0–2·3)	24 223	0·6 (0·3–1·3)	167 645	4·4 (3·3–6·0)	252 972	6·6 (5·2–8·7)
Australia and New Zealand	5	1·9 (1·0–3·1)	1	0·5 (0·2–1·5)	9	3·8 (2·5–5·3)	16	6·5 (5·0–8·3)
Central Asia and Southern Asia	8990	1·1 (0·5–2·3)	3561	0·4 (0·2–1·2)	27 682	3·4 (2·0–5·5)	42 348	5·2 (3·4–8·0)
Eastern Asia and South-eastern Asia	2377	0·9 (0·5–1·7)	1034	0·4 (0·2–0·9)	8003	3·0 (2·0–4·5)	11 939	4·4 (3·1–6·4)
Latin America and the Caribbean	1499	1·5 (1·4–1·9)	376	0·4 (0·3–0·6)	4599	4·8 (4·3–5·3)	6535	6·8 (6·2–7·5)
Northern America and Europe	360	2·2 (1·8–2·6)	81	0·5 (0·3–0·9)	450	2·7 (2·3–3·2)	901	5·4 (4·9–6·1)
Oceania*	75	1·0 (0·4–2·6)	29	0·4 (0·1–1·3)	244	3·3 (1·6–6·3)	377	5·2 (2·9–9·1)
Sub-Saharan Africa	38 078	1·5 (0·9–2·7)	16 977	0·7 (0·3–1·5)	117 580	4·7 (3·2–7·1)	178 272	7·1 (5·2–10·4)
Western Asia and Northern Africa	1697	1·4 (0·8–2·5)	714	0·6 (0·3–1·3)	5209	4·2 (3·0–6·2)	7914	6·4 (4·8–9·0)

Data are estimated counts of cause of death and % with 80% UI. Estimated number of deaths for haemorrhage or sepsis subgroups might not sum to the total number of deaths for haemorrhage or sepsis. Discrepancies are a consequence of how the estimates are generated in the Bayesian model. The output from the model provides samples of the estimates of interest (eg, the number of antepartum haemorrhage deaths), and the median of those samples is used to derive the point estimate. Therefore, the median number of deaths for each subgroup might not add to the total median for haemorrhage or sepsis deaths. UI=uncertainty interval.

* Including Australia and New Zealand.

In total, 111 countries reported at least one late maternal death between 42 days and 1 year postpartum. All SDG regions had at least one country reporting one or more late maternal death. Within an SDG region, the average ratio of late maternal to maternal deaths ranged from <0·01 to 0·07 (table 3). Only four countries conducted a confidential inquiry that included late maternal deaths (France, Morocco, UK, and USA); of these countries, France, the UK, and the USA all had ratios higher than 0·1. The UK reports a ratio higher than 1, meaning that there were more late deaths than those up to 42 days. The common causes of late maternal deaths reported were mental disorders, diseases of the nervous system, cardiomyopathy in the puerperium, other diseases of the circulatory system, and malignant neoplasm.

**Table 3:** Late maternal deaths by Sustainable Development Goal region

	**Number of countries or territories contributing data**	**Countries or territories contributing data**	**Mean ratio of late maternal deaths to maternal deaths up to day 42**
Australia and New Zealand	2	Australia, New Zealand	0·03
Central Asia and Southern Asia	7	Iran, Kazakhstan, Kyrgyzstan, Sri Lanka, Maldives, Tajikistan, Uzbekistan	0·01
Eastern Asia and South-eastern Asia	8	Brunei, Japan, South Korea, Myanmar, Malaysia, Philippines, Singapore, Thailand	0·03
Latin America and the Caribbean	30	Argentina, Antigua and Barbuda, Bahamas, Belize, Brazil, Barbados, Chile, Colombia, Costa Rica, Cuba, Dominican Republic, Ecuador, Grenada, Guatamala, Guyana, Honduras, Jamaica, Saint Lucia, Mexico, Nicaragua, Panama, Peru, Puerto Rico, Paraguay, El Salvador, Suriname, Trinidad and Tobago, Uruguay, Saint Vincent and the Grenadines, Venezuela	0·06
Northern America and Europe	38	Austria, Belgium, Bulgaria, Bosnia and Herzegovina, Belarus, Canada, Switzerland, Czech Republic, Germany, Denmark, Spain, Estonia, Finland, France, UK, Greece, Croatia, Hungary, Ireland, Iceland, Italy, Lithuania, Luxemburg, Latvia, Moldova, North Macedonia, Malta, Montenegro, Netherlands, Norway, Poland, Portugal, Romania, Serbia, Slovakia, Slovenia, Sweden, USA	0·05
Oceania excluding Australia and New Zealand	2	Fiji, Solomon Islands	<0·01
Sub-Saharan Africa	4	Cabo Verde, Mauritius, South Africa, Nigeria	0·01
Western Asia and Northern Africa	20	United Arab Emirates, Armenia, Bahrain, Cyprus, Egypt, Georgia, Iraq, Israel, Jordan, Kuwait, Lebanon, Libya, Morocco, Oman, State of Palestine, Qatar, Saudi Arabia, Syria, Tunisia, Türkiye	0·07

## Discussion

Similar to previous WHO estimates, obstetric haemorrhage remains the leading cause of maternal death globally, disproportionately affecting women in LMICs.^4^, ^5^ The existence of effective clinical interventions means deaths from haemorrhage are largely preventable.^13^ Hypertensive disorders remain the second most common direct obstetric cause of death and were the leading cause of maternal death in Latin America and the Caribbean. Increases in the proportion of women delivering in health-care facilities have not been sufficient to reduce deaths from haemorrhage or hypertensive disorders of pregnancy.^13^, ^14^ For hypertensive disorders, increases in the coverage of antenatal care potentially have not corresponded to adequate implementation of preventive strategies.^14^ Persistent regional heterogeneity in the contribution of these two leading direct obstetric causes of mortality is indicative of substantial inequities in access to, and the quality of, basic and emergency obstetric care.^14^

Our results indicate that most deaths from haemorrhage and sepsis occur in the postpartum period. The timing of these deaths emphasises the need for renewed commitment towards improving immediate and early postpartum care for haemorrhage, which typically occurs soon after delivery, and postpartum care after hospital discharge for the prevention of maternal deaths from sepsis.^15^, ^16^ Postpartum care consistently has the lowest coverage compared with antepartum and intrapartum care.^7^, ^17^ Historical neglect of the postpartum period and medium-to-long-term consequences of pregnancy and childbirth^15^ are also evidenced by the scarcity of data on late maternal deaths, especially in LMICs.^18^, ^19^ The data available suggest the distribution of causes of late maternal deaths differs from maternal deaths before 42 days, with indirect causes being more prevalent. We urgently need better reporting of late maternal deaths, consistent with ICD-11 coding.^18^, ^19^ Increased reporting of “comprehensive maternal deaths”—which include all deaths up to 1 year postpartum, introduced in ICD-11^20^—is encouraged to improve the visibility of deaths beyond 42 days postpartum.

There has been no substantial change in the proportion of maternal deaths from indirect causes since the two previous WHO analyses (20% in 2006^4^ and 27% in 2014^5^), which remain the second largest cause of maternal death.^15^ As the maternal mortality transition progresses—from high to low maternal mortality and direct to indirect causes—improving maternal outcomes is a dual challenge for health systems: to provide quality emergency obstetric care and to improve coordination with other medical specialties.^6^, ^15^, ^21^ This challenge will be best addressed by a health systems approach across the continuum of care, rather than vertical interventions focused on obstetric outcomes.^3^

This Article reports the first WHO maternal cause of death estimates for maternal suicide. Globally, only 12 countries reported at least one maternal death from suicide. The proportion of maternal deaths due to suicide varied by region, ranging from below 1% in sub-Saharan Africa, to 26% in Australia and New Zealand. Robust confidential enquiry systems in the UK and Ireland show that maternal suicide is currently the third largest cause of direct maternal deaths occurring during or within 42 days of pregnancy and the leading cause of direct late maternal deaths.^22^

Maternal suicide is considered a direct maternal death by WHO,^1^ but such deaths are not currently included in the MMEIG maternal mortality ratio. Within CRVS data, it is not possible to determine whether a death by suicide was temporal to pregnancy. Deaths due to suicide are generally stigmatised; misclassification of maternal suicide as a non-intentional death due to external injury can lead to underestimation of its prevalence.^23^ Systematic recording and reporting of maternal suicide should be prioritised, particularly in low-mortality settings where maternal suicide is a dominant cause of maternal death. Programmatic interventions are likely to be complex and require timely and efficient referrals and communication between different levels of the health system and with social welfare more broadly; robust data are an essential step in this chain.

There are only a few countries where maternal mortality is declining at a sufficiently rapid rate for global SDG target 3.1 for maternal mortality to be met.^2^ Accurate and complete data are crucial to leverage increased commitment to reducing maternal mortality and inform health systems' responses. Two important issues in maternal cause of death data exist: the extent to which deaths are identified, and misclassification of the cause of death.^24^ Indirect maternal deaths and late maternal deaths, which often take place away from the delivery ward and away from the time of delivery, are particularly vulnerable to these issues.^25^

Improving data availability requires strengthening routine data collection systems and, where possible, triangulating data sources to identify incomplete and misclassified deaths.^24^ In 2015, WHO released the Strategies toward Ending Preventable Maternal Mortality report, underscoring a need to improve measurement and to ensure that all maternal deaths are counted.^26^ This report encourages countries to develop national CRVS systems, implement standard death certificates, and use standardised definitions for coding of deaths consistent with ICD-11.^26^ Improving the availability of data on maternal causes of death is particularly crucial in sub-Saharan Africa, where many countries in our estimates were entirely model driven (ie, no data available; appendix pp 59–62).

This Article used a similar methodology to the last WHO estimates by Say and colleagues.^5^ The main change was a relaxation of the inclusion criteria to allow large subnational studies representative at administrative level 1 or higher to be included in the input dataset. The criterion to exclude health facility data where the institutional birth rate was below 50% was also removed, because few countries now have an institutional birth rate this low, and high facility birth rates do not always correspond to adequate quality of care or referral networks.^14^ Unlike the previous estimates, we also included studies that reported only one cause of death, so long as a suitable denominator was reported, to help identify data on abortion-related mortality, which are often published with a single-cause focus.

With these changes, we identified at least one observation for 129 (70%) of 185 countries included in the Article. The geographical coverage of our estimates also reiterates the utility of Bayesian hierarchical models for maternal mortality estimation in data scarce contexts, allowing sufficient flexibility to estimate the cause distribution, even in contexts without data.

Our study also has limitations. First, this study uses the broad categories for cause of death previously reported in global estimates^4^, ^5^ to ensure comparability with previous WHO estimates; however, these categories differ from ICD maternal mortality cause of death categories.^1^ Different groupings might suit different research or programmatic purposes. Second, as this study was conducted in two rounds (ie, 2009–17 and 2017–20), minor methodological changes were implemented to the search strategy, which might have introduced some heterogeneity over time. Third, resource constraints limited our capacity to systematically search for Chinese and Russian script studies. Finally, as noted previously, estimates are only as strong as input data, which are subject to incompleteness and misclassification, or which are not available in some countries. Variation between countries in reporting within and outside facility maternal deaths also introduces heterogeneity and could lead to underestimation of causes prevalent after discharge (eg, sepsis).

As recognised by the 2024 World Health Assembly resolution, stalling progress to reduce maternal mortality requires decisive action to recover lost progress in improving maternal survival.^27^ With haemorrhage remaining the leading cause of maternal death globally, the WHO Roadmap for Postpartum Haemorrhage 2023 to 2030 provides key priorities for research, norms and standards, implementation, and advocacy to tackle obstetric haemorrhage.^13^ These four strategic areas were identified by key stakeholders to accelerate progress towards reducing deaths from postpartum haemorrhage.^13^ The dual challenge of direct and indirect obstetric causes highlights the need for a health systems approach that integrates obstetric and non-obstetric providers across the continuum of care.^15^, ^21^

### Contributors

### Data sharing

The data (ie, input data to the models and analysis) are available on reasonable request to the corresponding author. Our code repository and data from the WHO Mortality Database are openly available at https://www.who.int/data/data-collection-tools/who-mortality-database.

## Declaration of interests

Five WHO staff members (JAC, DC, A-BM, ÖT, and LS) are part of the team that conducted the study. The findings in this Article represent the conclusions of the authors. The named authors alone are responsible for the views expressed in this publication, which do not necessarily represent the decisions or the policies of the UNDP–UNFPA–UNICEF–WHO–World Bank Special Programme of Research, Development, and Research Training in Human Reproduction (HRP) or WHO. GV and YS have been employees of Cochrane Response since 2017 and 2019, respectively. Cochrane Response was commissioned by WHO to undertake tasks relevant to this Article. All other authors declare no competing interests.
