# Supplementary material for: Global and regional causes of maternal deaths 2009–20: a WHO systematic analysis
Source: Lancet Glob Health. 2025 Mar 8;13(4):e626–34. doi: 10.1016/S2214-109X(24)00560-6 (PMC11946934; doi:10.1016/S2214-109X(24)00560-6)
Supplement: Supplementary appendix [file mmc1.pdf]

THE LANCET
Global Health

Supplementary appendix

This appendix formed part of the original submission and has been peer reviewed. We post it as supplied by the authors.

Supplement to: Cresswell JA, Alexander M, Chong MYC, et al. Global and regional causes of maternal deaths 2009–20: a WHO systematic analysis. *Lancet Glob Health* 2025; published online March 7. https://doi.org/10.1016/S2214-109X(24)00560-6.

SUPPLEMENTARY MATERIAL: Global and regional causes of maternal deaths 2009-2020: a WHO systematic analysis

Jenny A. Cresswell, PhD[1], Monica Alexander PhD[2,3], Michael Y.C. Chong[2], Heather M. Link MD[4], Marija Pejchinovska, MSc[2], Ursula Gazeley, PhD[5], Sahar Ahmed, MD[1], Doris Chou, MD[1], Ann-Beth Moller, MPH[1], Daniel Simpson, PhD[6], Professor Leontine Alkema, PhD[7], Gemma Villanueva[8], MSc [2], Yanina Sguassero, PhD[8], Özge Tunçalp, MD [1], Professor Qian Long, MD[9], Shaoming Xiao, PhD[10], Lale Say, MD[1]

Correspondence

Correspondence to Jenny A. Cresswell, cresswellj@who.int, UNDP-UNFPA-UNICEF-WHO-World Bank Special Programme of Research, Development and Research Training in Human Reproduction (HRP), Department of Sexual and Reproductive Health and Research, World Health Organization, 20 Avenue Appia, 1211 Geneva, Switzerland.

Affiliations

[1] UNDP-UNFPA-UNICEF-WHO-World Bank Special Programme of Research, Development and Research Training in Human Reproduction (HRP), Department of Sexual and Reproductive Health and Research, World Health Organization, 20 Avenue Appia, 1211 Geneva, Switzerland.

[2] Department of Statistical Sciences, University of Toronto, Canada

[3] Department of Sociology, University of Toronto, Canada

[4] Kaleida Health, Division of Maternal Fetal Medicine, Buffalo NY 14226, USA

[5] Department of Infectious Disease Epidemiology, London School of Hygiene and Tropical Medicine, London WC1E 7HT, United Kingdom

[6] Department of Econometrics and Business Statistics, Monash University, Melbourne, Australia

[7] Department of Biostatistics and Epidemiology, University of Massachusetts Amherst, 715 North Pleasant Street, USA

[8] Cochrane Response, Cochrane, 11-13 Cavendish Square, London, W1G 0AN, United Kingdom

[9] Global Health Research Center, Duke Kunshan University, No. 8 Duke Avenue, Kunshan, Jiangsu Province, China 215316

[10] Johns Hopkins University School of Medicine, Baltimore, Maryland, USA

Contents

CONTENTS 2

TABLES 2

FIGURES 2

APPENDIX 1: GATHER CHECKLIST 4

APPENDIX 2: SEARCH STRATEGY 6

APPENDIX 3: ARTIFICIAL INTELLIGENCE CLASSIFIER MODEL 45

APPENDIX 4: PROCESS FOR ASSIGNING DEATHS TO AN ICD CODE OR GROUPING WHERE THE CAUSE OF DEATH WAS REPORTED IN FREE TEXT FORMAT 46

APPENDIX 5: DETAILS OF THE STATISTICAL MODEL 51

APPENDIX 6: SUSTAINABLE DEVELOPMENT GOAL (SDG) REGIONAL CLASSIFICATION 57

APPENDIX 7: COUNTRIES WITH AND WITHOUT OBSERVED DATA 59

APPENDIX 8: GLOBAL DISTRIBUTION OF MATERNAL DEATHS BY SDG REGION 63

APPENDIX 9: MATERNAL DEATHS DUE TO SUICIDE 65

Tables

Table S1 Gather checklist 4
Table S2 Details of Member States website search 7
Table S3 Classification of deaths by ICD codes 49
Table S4 Bayesian hierarchical model regional classification 52
Table S5 Sustainable Development Goal regional classification 57
Table S6 Countries without observed data by Sustainable Development Goal region 59
7 Table S7 Global Distribution of Maternal Deaths by SDG Region 63
8 Table S8 Maternal Deaths due to Suicide 65

Figures

Figure S1 PRISMA for Search 1a 36
Figure S2 PRISMA for Search 1b 37
Figure S3 PRISMA for Search 1C 38

Figure S4 PRISMA for Search 2 44

APPENDIX 1: GATHER CHECKLIST

Table S1 Gather checklist

Item #	Checklist item	Reported on page #
**Objectives and funding**		
1	Define the indicator(s), populations (including age, sex, and geographic entities), and time period(s) for which estimates were made.	2
2	List the funding sources for the work.	2
**Data Inputs**		
For all data inputs from multiple sources that are synthesized as part of the study:		
3	Describe how the data were identified and how the data were accessed.	5,6
4	Specify the inclusion and exclusion criteria. Identify all ad-hoc exclusions.	5
5	Provide information on all included data sources and their main characteristics. For each data source used, report reference information or contact name/institution, population represented, data collection method, year(s) of data collection, sex and age range, diagnostic criteria or measurement method, and sample size, as relevant.	6
6	Identify and describe any categories of input data that have potentially important biases (e.g., based on characteristics listed in item 5).	6
For data inputs that contribute to the analysis but were not synthesized as part of the study:		
7	Describe and give sources for any other data inputs.	6
For all data inputs:		
8	Provide all data inputs in a file format from which data can be efficiently extracted (e.g., a spreadsheet rather than a PDF), including all relevant meta-data listed in item 5. For any data inputs that cannot be shared because of ethical or legal reasons, such as third-party ownership, provide a contact name or the name of the institution that retains the right to the data.	NA
**Data analysis**		
9	Provide a conceptual overview of the data analysis method. A diagram may be helpful.	7
10	Provide a detailed description of all steps of the analysis, including mathematical formulae. This description should cover, as relevant, data cleaning, data pre-processing, data adjustments and weighting of data sources, and mathematical or statistical model(s).	7
11	Describe how candidate models were evaluated and how the final model(s) were selected.	8

12	Provide the results of an evaluation of model performance, if done, as well as the results of any relevant sensitivity analysis.	Appendix 5
13	Describe methods for calculating uncertainty of the estimates. State which sources of uncertainty were, and were not, accounted for in the uncertainty analysis.	8
14	State how analytic or statistical source code used to generate estimates can be accessed.	8
**Results and Discussion**		
15	Provide published estimates in a file format from which data can be efficiently extracted.	Tables
16	Report a quantitative measure of the uncertainty of the estimates (e.g. uncertainty intervals).	Throughout
17	Interpret results in light of existing evidence. If updating a previous set of estimates, describe the reasons for changes in estimates.	12,13
18	Discuss limitations of the estimates. Include a discussion of any modelling assumptions or data limitations that affect interpretation of the estimates.	15

APPENDIX 2: SEARCH STRATEGY

Data obtained from the WHO Maternal Mortality Database

The WHO Mortality Database is a compilation of mortality data as reported annually by Member States from their civil registration systems. The database only contains medically certified deaths (not deaths reported by lay-persons), using ICD codes. The data appear as submitted by Member States, WHO makes no adjustment for under-coverage.

The WHO Mortality Database is available online here: https://www.who.int/data/data-collection-tools/who-mortality-database

All deaths recorded as being due to any codes from obstetric causes in Chapter 15 of ICD-10 “Pregnancy, Childbirth and the Puerperium” (the “O-codes”) in addition to code A34 (obstetrical tetanus) occurring between 2009 and 2020 were extracted and included in this study.

Data obtained from the WHO Archive of reports received from Member States and Hand-searching of Member State websites

Member States regularly provide WHO with copies of confidential enquiries, censuses and other official publications reporting on maternal death, both ongoing and via a country consultation conducted as part of the MMR estimation process.

For search 1, this archive was hand-searched to identify relevant data, in particular those reports received for the country consultation conducted between May to June 2019 for the MMEIG maternal mortality estimates: *Trends in maternal mortality 2000 to 2017: estimates by WHO, UNICEF, UNFPA, World Bank Group and the United Nations Population Division. Geneva: World Health Organization; 2019. Licence: CC BY-NC-SA 3.0 IGO.*

For search 2, we searched additional reports received during the country consultation conducted between August and October 2022 for the updated MMEIG maternal mortality estimates: *Trends in maternal mortality 2000 to 2020: estimates by WHO, UNICEF, UNFPA, World Bank Group and UNDESA/Population Division; 2023. Licence: CC BY-NC-SA 3.0 IGO*

For search 1 only, this was also supplemented by a hand-search of the websites of the Ministry of Health (MOH) and National Statistics Office (NSO) of Member States conducted in June to July 2019. Table S2 shows the websites searched for the 2009-2017 update:

Table S2 Details of Member States website search

Member State	MINISTRY OF HEALTH	NATIONAL STATISTICS OFFICE
Algeria	http://www.sante.gov.dz/	http://www.ons.dz/
Angola	http://www.minsa.gov.ao/	https://www.ine.gov.ao/
Benin	http://beninmoh.eu5.org/index1.html	https://www.insae-bj.org/
Botswana	https://www.moh.gov.bw/	http://www.statsbots.org.bw/
Burkina Faso	http://www.sante.gov.bf/	http://www.insd.bf/n/
Burundi	http://minisante.bi/	http://www.isteebu.bi/
Cameroon	http://www.minsante.cm/	http://www.statistics-cameroon.org/
Cape Verde	https://www.minsaude.gov.cv/	http://ine.cv/en/
Central African Republic	Website not identified	http://www.stat-centrafrique.com/
Chad	https://sante-tchad.org/	http://www.inseed-td.net/
Comoros	https://www.gouvernement.km/	
Congo	http://www.sante.gouv.cg/	http://www.cnsee.org/
Côte d'Ivoire	http://www.sante.gouv.ci/	http://www.ins.ci/n/
Democratic Republic of the Congo	https://www.minisanterdc.cd/	http://ins-rdc.org/
Equatorial Guinea	https://www.guineaecuatorialpress.com/	
Eritrea	http://www.shabait.com/	
Ethiopia	http://www.moh.gov.et/ejcc/	www.csa.gov.et but “website could not be retrieved”
Gabon	http://www.sante.gouv.ga/	http://www.stat-gabon.org/
Gambia	www.moh.gov.gm/ but “website could not be retrieved”	www.gbos.gov.gm but “website could not be retrieved”
Ghana	http://www.moh.gov.gh/	http://www.statsghana.gov.gh/
Guinea	http://sante.gov.gn/	http://www.stat-guinee.org/
Guinea-Bissau	Website not found	http://www.stat-guinebissau.com/
Kenya	http://www.health.go.ke/	https://www.knbs.or.ke/
Lesotho	https://www.gov.ls/ministry-of-health/	http://www.bos.gov.ls/
Liberia	http://moh.gov.lr/	http://www.lisgis.net/
Madagascar	http://www.sante.gov.mg/home/n	https://www.instat.mg/
Malawi	http://www.health.gov.mw/	http://www.nsomalawi.mw/
Mali	http://www.sante.gov.ml/	http://www.instat-mali.org/
Mauritania	http://www.sante.gov.mr/ar/	http://www.ons.mr/
Mauritius	http://health.govmu.org/English/Pages/default.aspx	http://statsmauritius.govmu.org/English/Pages/default.aspx
Mozambique	http://www.misau.gov.mz/ but “website could not be retrieved”	http://www.ine.gov.mz/
Namibia	http://www.mhss.gov.na/	https://nsa.org.na/
Niger	http://www.msp.ne/	http://www.stat-niger.org/statistique/
Nigeria	https://www.health.gov.ng/	https://www.nigerianstat.gov.ng/
Rwanda	http://www.moh.gov.rw/	http://www.statistics.gov.rw/
Sao Tome and Principe	http://saude.portal-stp.net/spip.php?rubrique1 but “website could not be retrieved”	https://www.ine.st/
Senegal	http://www.sante.gouv.sn/ but “website could not be retrieved”	http://www.ansd.sn/
Seychelles	http://www.health.gov.sc/	https://www.nbs.gov.sc/
Sierra Leone	http://health.gov.sl/	https://www.statistics.sl/
South Africa	http://www.health.gov.za/	http://www.statssa.gov.za/
Swaziland	http://www.gov.sz/index.php/ministries-departments/ministry-of-health	http://www.swazistats.org.sz/
Tanzania, United Republic of	http://www.moh.go.tz/en/	http://www.nbs.go.tz/index.php/en/
Togo	http://www.togoleseministryofhealthlome.mywebsite.com/	http://www.stat-togo.org/
Uganda	https://health.go.ug/	https://www.ubos.org/
Zambia	http://www.moh.gov.zm/	https://www.zamstats.gov.zm/
Zimbabwe	www.mohcc.gov.zw/ Server times out	www.zimstat.co.zw/
Afghanistan	https://moph.gov.af/en	http://cso.gov.af/en
Bahrain	https://www.moh.gov.bh	http://www.data.gov.bh/
Djibouti	http://www.sante.gouv.dj/	http://www.ministere-finances.dj/
Egypt	http://www.mohp.gov.eg	http://www.sis.gov.eg
Iran, Islamic Republic of	http://behdasht.gov.ir	https://www.amar.org.ir/english
Iraq	https://moh.gov.iq	http://www.cosit.gov.iq/en/
Jordan	http://www.moh.gov.jo	http://dosweb.dos.gov.jo

Kuwait	https://www.moh.gov.kw/en	https://www.csb.gov.kw/Default_EN
Lebanon	https://www.moph.gov.lb/en	http://www.cas.gov.lb
Libya	http://seha.ly/en/	http://www.bsc.ly
Morocco	https://www.sante.gov.ma/	https://www.men.gov.ma
Oman	https://www.moh.gov.om/ar/1	https://www.ncsi.gov.om
Pakistan	http://www.nhsrc.gov.pk	http://www.pbs.gov.pk
Qatar	https://www.moph.gov.qa	https://www.mdps.gov.qa
Saudi Arabia	https://www.moh.gov.sa	https://www.stats.gov.sa/en
Somalia	http://moh.gov.so/en/	http://www.dns.org.so
Sudan	http://www.sho.gov.sd/	http://cbs.gov.sd/index.php/en/
South Sudan	https://moh-rss.org	http://ssnbs.org but "website could not be retrieved"
Syrian Arab Republic	http://www.moh.gov.sy/en	http://cbssyr.sy/index-EN.htm "This service is unavailable"
Tunisia	http://www.santetunisie.rns.tn/fr/	http://www.ins.tn/en/front
United Arab Emirates	http://www.mohap.gov.ae/en	http://fcsa.gov.ae/en-us
Yemen	http://www.mophp-ye.org/english/minister_Yassin.html	http://www.cso-yemen.org "This service is unavailable"
Albania	http://shendetesia.gov.al	http://www.instat.gov.al
Andorra	https://www.salut.ad	https://www.estadistica.ad/serveiestudis/web/index.asp?lang=4
Armenia	http://moh.am/#1/0	https://www.armstat.am
Austria	http://www.bmg.gv.at/home	https://www.statistik.at/web_de/statistiken/index.html
Azerbaijan	http://www.sehiyye.gov.az	https://www.stat.gov.az/?lang=en
Belarus	http://minzdrav.gov.by/en/	http://www.belstat.gov.by/en/
Belgium	https://www.health.belgium.be/en	http://statbel.fgov.be/en
Bosnia and Herzegovina	http://www.fmoh.gov.ba	http://www.bhas.ba/?option=com_content&view=article&id=46&lang=en
Bulgaria	http://www.mh.government.bg/en/	http://www.nsi.bg/en
Croatia	https://zdravstvo.gov.hr	https://www.dzs.hr/default_e.htm
Cyprus	https://www.moh.gov.cy	https://www.mof.gov.cy
Czech Republic	https://www.mzcr.cz/en/	https://www.czso.cz/csu/czso/home
Denmark	http://www.sum.dk/	https://www.dst.dk/en.aspx
Estonia	https://www.sm.ee/en	https://www.stat.ee/en
Finland	https://stm.fi/en/frontpage	https://www.stat.fi/index_en.html
France	https://solidarites-sante.gouv.fr/	https://www.insee.fr/en/accueil
Georgia	https://www.moh.gov.ge/en/	https://www.geostat.ge/en
Germany	https://www.bundesgesundheitsministerium.de/en/en.html	https://www.destatis.de/EN/Home/_node.html
Greece	http://www.moh.gov.gr/	https://www.statistics.gr/en/home/
Hungary	https://www.kormany.hu/en/ministry-of-human-resources	https://www.ksh.hu/?lang=en
Iceland	https://www.landlaeknir.is/english/	https://www.statice.is
Ireland	https://health.gov.ie	https://cso.ie
Israel	https://www.health.gov.il/English/Pages/HomePage.aspx	https://www.cbs.gov.il/en
Italy	http://www.salute.gov.it/portale/p5_11.jsp	https://www.istat.it/en/
Kazakhstan	http://dsm.gov.kz/en	http://stat.gov.kz
Kyrgyzstan	http://www.med.kg/ru/	http://www.stat.kg/en/
Latvia	https://www.vm.gov.lv/en/	https://www.csb.gov.lv/en/sakums
Lithuania	http://sam.lrv.lt/en/	https://www.stat.gov.lt/en
Luxembourg	http://sante.public.lu/fr/politique-sante/ministere-sante/index.html	https://statistiques.public.lu/en/actors/statec/index.html
Macedonia, the former Yugoslav Republic of	http://zdravstvo.gov.mk	http://www.stat.gov.mk/Default_en.aspx
Malta	https://deputyprimeminister.gov.mt/en/Pages/health.aspx	https://nso.gov.mt
Moldova, Republic of	https://msmps.gov.md/en	http://statistica.gov.md/pageview.php?l=en&idc=263&id=2193
Monaco	https://en.gouv.mc/Government-Institutions/The-Government/Ministry-of-Health-and-Social-Affairs	http://www.monacostatistics.mc
Montenegro	http://www.mzdravlja.gov.me/en/ministry	https://www.monstat.org/eng/
Netherlands	https://www.government.nl/ministries/ministry-of-health-welfare-and-sport	https://www.cbs.nl/en-gb

Norway	https://www.helsedirektoratet.no/english	https://www.ssb.no/en
Poland	http://www.mz.gov.pl	https://stat.gov.pl/en/
Portugal	https://www.min-saude.pt/portal	https://www.ine.pt/xportal/xmain?xpid=INE&xpgid=ine_main
Romania	http://www.ms.ro	http://www.insse.ro/cms/en
Russian Federation	https://www.rosminzdrav.ru/	http://www.gks.ru/wps/wcm/connect/rosstat_main/rosstat/en/main/
San Marino	http://www.sanita.sm/on-line/home.html	http://www.statistica.sm/on-line/en/home.html
Serbia	https://www.zdravlje.gov.rs/	http://www.stat.gov.rs/en-us/
Slovakia	https://www.health.gov.sk/Titulka	https://slovak.statistics.sk
Slovenia	http://www.mz.gov.si/en/	https://www.stat.si/StatWeb/en
Spain	https://www.mscbs.gob.es/en/home.htm	https://www.ine.es/en/
Sweden	https://www.government.se/government-of-sweden/ministry-of-health-and-social-affairs/	https://www.scb.se/en/
Switzerland	https://www.bag.admin.ch/bag/en/home.html	https://www.bfs.admin.ch/bfs/en/home.html
Tajikistan	http://moh.tj/?lang=en	https://www.stat.tj/en/
Turkey	http://www.kanser.gov.tr	http://www.turkstat.gov.tr/VeriBilgi.do?alt_id=39
Turkmenistan	http://www.saglykhm.gov.tm	http://www.stat.gov.tm
Ukraine	http://en.moz.gov.ua	https://ukrstat.org/en/menu/publikac_e.htm
United Kingdom	https://www.gov.uk/government/organisations/department-of-health-and-social-care	https://www.npeu.ox.ac.uk/mbrrace-uk
Uzbekistan	https://www.minzdrav.uz/en/	https://stat.uz/en/
Antigua and Barbuda	https://ab.gov.ag/detail_page.php?page=29	https://statistics.gov.ag
Argentina	https://www.argentina.gob.ar/salud	https://www.indec.gov.ar/el-indec-eng.asp “HTTP Error 404. The requested resource is not found.”
Bahamas	http://www.bahamas.gov.bs/wps/portal/public/!ut/p/b1/04_Sj9CPykssy0xPLMnMz0vMAfGjzOKNDdx9HR1NLHz9jUIsDTwNnQ3NvENNDSxczIAKIoEKDHAARwNC-sP1o_ArMYEqwGOFn0d-bqp-QW6EQZaJoyIAQ77YBg!!/dl4/d5/L2dBISEvZ0FBIS9nQSEh/	http://www.bahamas.gov.bs/wps/portal/public/!ut/p/b1/vdPLkqIwGAXgZ_EBIHCHJQhy0QQJiMAmhYCICKIioE_fztTspnp6M0Wyyl8n-eosQsVUSMVN0pdF0pXXJrn8OscCcVXgehuZVSRN54AicIqo8pgFK5raU6EZPcalZA26fiBemztcsB48PK5SDM2WTfBrHiQ-UyR13xPDfs3lkZcgRHAfdfPIuXcceyEhyR_NZs-C_mCxztlQXZLtnyzTho_2dnGDW6c5apA5oRGi4wvZcFWxaeNpkNel-uRg5Jf6iJcCzMw44oq865cyUZb9PrLGT4noUwJ8sxTwc8f4d4QFBlQUToIGDwRg7bYbZSuajMOBP4F_ENEnIH6LBIDyqRBwxDu_WutdvfH57Q4Q0Aie4ecBeo0qskO7i4M6FaENtj4zB2kIeYcl-CK2YHfcZgHeqYrWjWY2_wmkpwbZaUEDcFODwtQgPzFogKlBZmrw_38Lm4rLQ70Y0noBFjTP8RwQBAAkWWAAQwV29JK1qzWs9PioRomYPslj3ehvrRQtt7q1zNWft1vGo-95U-V12jjmMK6lXl9Klb3e74aEfwJo4GBzFH3V9hm9YNQoOKKlK-RqsW162Rvw1ibh6nD_XH9ErF0zybn07mqX3VYZyR4q352y_CADkpqnel7XymxGIfNa51Rb9xsBS_Rfmy1mXwSp7a8!/dl4/d5/L2dBISEvZ0FBIS9nQSEh/
Barbados	http://www.health.gov.bb	http://www.barstats.gov.bb “HTTP Error 404. The requested resource is not found.”
Belize	http://health.gov.bz/www/	http://sib.org.bz
Bolivia	https://www.minsalud.gob.bo	https://www.ine.gob.bo
Brazil	http://portalms.saude.gov.br	https://ww2.ibge.gov.br/english/
Canada	https://www.canada.ca/en/health-canada.html	https://www.statcan.gc.ca/eng/start
Chile	https://www.minsal.cl	http://www.ine.cl
Colombia	https://www.minsalud.gov.co/English/Paginas/inicio.aspx	https://www.dane.gov.co/index.php/en/
Costa Rica	https://www.ministeriodesalud.go.cr	http://www.inec.go.cr
Cuba	http://www.sld.cu	http://www.one.cu
Dominica	http://dominica.gov.dm	http://finance.gov.dm/statistics
Dominican Republic	http://www.sespas.gov.do “Can’t find server”	https://www.one.gob.do

Ecuador	https://www.salud.gob.ec	https://www.ecuadorencifras.gob.ec/institucional/home/
El Salvador	http://www.salud.gob.sv	http://www.digestyc.gob.sv "Can't find server"
Grenada	http://health.gov.gd/index.php?lang=en	http://stats.gov.gd
Guatemala	https://www.mspas.gob.gt	https://www.ine.gob.gt
Guyana	https://www.health.gov.gy	https://statisticsguyana.gov.gy
Haiti	http://mspp.gouv.ht/newsite/	http://www.ihsi.ht
Honduras	http://www.salud.gob.hn/site/	https://www.ine.gob.hn
Jamaica	https://www.moh.gov.jm	https://statinja.gov.jm
Mexico	https://www.gob.mx/salud/en	http://en.www.inegi.org.mx
Nicaragua	http://www.minsa.gob.ni	http://www.inide.gob.ni
Panama	http://www.minsa.gob.pa	http://www.contraloria.gob.pa/inec/
Paraguay	https://www.mspbs.gov.py/portal	http://www.dgeec.gov.py
Peru	https://www.gob.pe/minsa/	http://www.inei.gob.pe
Saint Kitts and Nevis	https://www.gov.kn/moh	https://www.facebook.com/pages/category/Public---Government-Service/St-Kitts-Statistics-Department-247905702589265/
Saint Lucia	http://health.govt.lc	https://www.stats.gov.lc
Saint Vincent and the Grenadines	http://www.health.gov.vc	http://www.stats.gov.vc
Suriname	http://www.gov.sr/ministerie-van-volksgezondheid "Can't find server"	http://www.statistics-suriname.org
Trinidad and Tobago	http://www.health.gov.tt	http://cso.gov.tt
United States of America	https://www.hhs.gov	https://www.census.gov
Uruguay	http://www.msp.gub.uy	http://www.ine.gub.uy
Venezuela	http://www.mpps.gob.ve	http://www.ine.gov.ve
Bangladesh	http://www.mohfw.gov.bd	http://203.112.218.65:8008/Home.aspx
Bhutan	http://www.health.gov.bt	http://www.nsb.gov.bt
Democratic Peoples Republic of Korea	Website not found	Website not found
India	https://mohfw.gov.in	http://www.mospi.gov.in
Indonesia	http://www.depkes.go.id/index.php?lg=LN02	https://www.bps.go.id
Maldives	http://www.health.gov.mv	http://statisticsmaldives.gov.mv
Myanmar	https://www.mohs.gov.mm	https://www.csostat.gov.mm
Nepal	https://www.mohp.gov.np/eng/	https://www.cbs.gov.np
Sri Lanka	http://www.health.gov.lk/moh_final/english/	http://www.statistics.gov.lk
Thailand	http://www.moph.go.th	http://www.nso.go.th/sites/2014en
Timor-Leste	http://www.moh.gov.tl	http://www.statistics.gov.tl
Australia	https://www.aihw.gov.au	https://www.abs.gov.au
Brunei Darussalam	http://www.moh.gov.bn	http://www.depd.gov.bn/SitePages/National%20Statistics.aspx
Cambodia	http://moh.gov.kh/?lang=en	https://www.nis.gov.kh/index.php/en/
China	http://en.nhc.gov.cn	http://www.stats.gov.cn/english/
Cook Islands	https://www.health.gov.ck	http://www.mfem.gov.ck/statistics
Fiji	https://www.health.gov.fj	https://www.statsfiji.gov.fj/index.php
Japan	https://www.mhlw.go.jp/english/	https://www.stat.go.jp/english/
Kiribati	http://www.president.gov.ki/ministry-of-health-and-medical-services/	http://www.mfed.gov.ki/statistics/
Korea, Republic of	http://www.mohw.go.kr/eng/	http://kostat.go.kr/portal/eng/index.action
Lao People's Democratic Republic	http://www.moh.gov.la/index.php/lo-la/	https://www.lsb.gov.la/en/
Malaysia	http://www.moh.gov.my	https://www.dosm.gov.my/v1/
Marshall Islands	https://www.facebook.com/rmimoh/	https://rmi.prism.spc.int
Micronesia, Federated States of	http://www.fsmhealth.fm "Can't find server"	http://www.fsmstatistics.fm/?page_id=105
Mongolia	http://www.mohs.mn	http://www.en.nso.mn/index.php
Nauru	http://www.naurugov.nr	https://nauru.prism.spc.int
New Zealand	https://www.health.govt.nz	https://www.hqsc.govt.nz
Niue	http://www.gov.nu/wb/pages/ministries.php	https://niue.prism.spc.int
Palau	http://www.palauhealth.org	http://palaugov.pw/statistics/
Papua New Guinea	http://www.health.gov.pg	https://www.nso.gov.pg
Philippines	https://www.doh.gov.ph	https://psa.gov.ph
Samoa	http://www.health.gov.ws	http://www.sbs.gov.ws
Singapore	https://www.moh.gov.sg	https://www.singstat.gov.sg
Solomon Islands	https://www.facebook.com/pages/Ministry-of-Health-and-Medical-Services/1389486501290579	https://www.statistics.gov.sb
Tonga	http://www.health.gov.to	https://tonga.prism.spc.int

Tuvalu	Website not found	https://tuvalu.prism.spc.int/index.php/contact
Vanuatu	http://www.governmentofvanuatu.gov.vu/index.php?option=com_content&view=article&id=93&Itemid=130	https://vnso.gov.vu
Viet Nam	https://www.moh.gov.vn/en_US/web/ministry-of-health	https://www.gso.gov.vn/Default_en.aspx?tabid=766

Data obtained from the search of the bibliographic databases

Search 1: Data from 2009-2017

For Search 1, searches were separated by script: Search 1A covered MEDLINE, Embase, Global Index Medicus, Web of Science, Popline; Search 1B covered Chinese-script databases (Wanfang, CNKI); Search 1C used a Russian-script database (eLIBRARY.RU). For Search 1A, two reviewers screened for eligibility and a third reviewer adjudicated. Chinese and Russian-script publications were screened by a single reviewer. Overall, 74 272 citations were identified; full text was screened for 5 136 sources, and 15 were eligible Search terms are listed below.

Search 1A: English language databases

The following searches were conducted in April 2019.

MEDLINE

1 Maternal Death/ (604)
2 (mortal or mortality or mortalities).tw,kf. (699826)
3 (dead or death or deaths or decease* or demise* or die or died or dies or dying).tw,kf. (1048125)
4 Fatal Outcome/ (63559)
5 (fatal or fatality or fatalities).tw,kf. (138291)
6 (non-survival* or nonsurvival*).tw,kf. (894)
7 "not surviv*".tw,kf. (4574)
8 Suicide/ (38332)
9 suicid*.tw,kf. (72020)
10 Homicide/ (13309)
11 (homicid* or kill or killed or kills or killing or murder*).tw,kf. (155590)
12 or/2-11 [DEATH/FATALITY/MORTALITY] (1857265)
13 exp Pregnancy/ (896173)
14 exp Pregnancy Complications/ (425515)
15 Pregnant Women/ (6883)
16 exp Pregnancy Trimesters/ (40851)
17 pregnan*.tw,kf. (503741)
18 (parturition* or childbirth* or child birth*).tw,kf. (31015)
19 (gravidity or primigrav* or primi-grav* or multigrav* or multi-grav* or nulligrav* or nulli-grav* or parity or primipar* or primi-par* or multipar* or multi-par* or nullipar* or nulli-par*).tw,kf. (74496)
20 dystocia*.tw,kf. (4235)
21 ((uterin* or uterus or uteri*) adj1 inert*).tw,kf. (282)
22 ((labor* or labour*) adj3 induc*).tw,kf. (10808)
23 ((labor* or labour*) adj3 obstruct*).tw,kf. (1154)
24 ((labor* or labour*) adj3 (delayed or failed or false or prolong*)).tw,kf. (2921)
25 ((labor* or labour*) adj3 precipitate*).tw,kf. (127)

26 ((labor* or labour*) adj3 (birth* or deliver*)).tw,kf. (9707)
27 ((abdominal* or vaginal*) adj3 (birth* or deliver*)).tw,kf. (19660)
28 (breech adj3 (birth* or deliver* or extract*)).tw,kf. (2075)
29 ((forceps or instrumental or vacuum) adj3 (birth* or deliver* or extract*)).tw,kf. (5544)
30 ((premature* or pre-mature* or preterm* or pre-term*) adj3 (birth* or deliver* or labor* or labour*)).tw,kf. (44835)
31 ((premature* or pre-mature* or preterm* or pre-term*) adj3 (membran* adj2 ruptur*)).tw,kf. (4719)
32 (caesarean* or cesarean* or "c-section" or "c-sections" or post-caesarean* or post-cesarean*).tw,kf. (58393)
33 (VBAC or VBACs).tw,kf. (647)
34 (contract* adj3 (hypertonic or incoordinate* or prolonged)).tw,kf. (1211)
35 (episiotom* or (perineal adj1 lacerat*)).tw,kf. (3125)
36 ((hydatidiform or nonhydatidiform or non-hydatidiform) adj mole?).tw,kf. (3572)
37 ((multiple* or twin or twins or triplet?) adj gestation*).tw,kf. (3675)
38 Maternal Welfare/ (7066)
39 Maternal Health/ (672)
40 (maternal* or maternit* or mother*).tw,kf. (397484)
41 Prenatal Care/ (25849)
42 Postnatal Care/ (5182)
43 Peripartum Period/ (887)
44 Postpartum Period/ (24335)
45 (antenatal* or ante-natal* or antepartum* or ante-partum* or intrapartum* or intra-partum* or perinatal* or peri-natal* or peripartum* or peri-partum* or prenatal* or pre-natal* or postnatal* or post-natal* or postpartum* or post-partum* or puerperal* or puerperium).tw,kf. (338675)
46 (hyperemesis gravidarum or morning sickness*).tw,kf. (1672)
47 (gestation* adj2 diabet*).tw,kf. (12904)
48 (gestation* adj2 (edema* or hypertensi* or oedema* or proteinuria*)).tw,kf. (3094)
49 (eclamp* or pre-eclamp* or HELLP).tw,kf. (17794)
50 ((EPH or "Edema-Proteinuria-Hypertension" or "Hypertension-Edema-Proteinuria" or "Proteinuria-Edema-Hypertension") adj2 (complex* or gestos#s or tox?emi*)).tw,kf. (575)
51 (placenta* adj3 (disease* or disorder* or syndrome*)).tw,kf. (1582)
52 (placenta* adj3 (abrupti* or accreta* or previa* or retained)).tw,kf. (7964)
53 (placenta* adj3 insufficien*).tw,kf. (1980)
54 (placenta* adj3 (prematur* adj2 separat*)).tw,kf. (181)
55 (amnionit#s or chorioamnionit#s or funisit#s).tw,kf. (4080)
56 (amnio* adj3 infect*).tw,kf. (1154)
57 (amnio* adj3 emboli*).tw,kf. (1251)
58 (gestation$2 adj1 pemphigoid*).tw,kf. (286)
59 (hydramnios or oligohydramnios or polyhydramnios).tw,kf. (5286)
60 vasa previa.tw,kf. (210)
61 exp Obstetric Surgical Procedures/ (132397)
62 obstetric*.tw,kf. (94957)
63 miscarr*.tw,kf. (12903)
64 abort*.tw,kf. (80102)
65 Abortion, Criminal/ (2202)
66 (postabort* or post-abort*).tw,kf. (2166)
67 exp Abortifacient Agents/ (62875)
68 Vacuum Curettage/ (1056)
69 ((dilatat* or dilat* or vacuum* or suction*) adj2 curettage*).tw,kf. (2320)
70 ((uterus or uteri or uterin* or vacuum*) adj2 aspirat*).tw,kf. (1381)

71 ((dilatat* or dilat*) adj2 evacuat*).tw,kf. (434)
72 or/13-71 [PREGNANCY/OBSTETRICS/MATERNAL POPULATION] (1484548)
73 12 and 72 (147416)
74 1 or 73 [DEATH/FATALITY/MORTALITY - PREGNANCY/OBSTETRICS/MATERNAL POPULATION] (147454)
75 Maternal Mortality/ (10295)
76 (maternal* adj3 mortalit*).tw,kf. (14096)
77 Cause of Death/ (47242)
78 Mortality/ (42222)
79 Hospital Mortality/ (35834)
80 mo.fs. [MORTALITY, I.E., MORTALITY STATISTICS] (560181)
81 Maternal Death/et [Etiology] (85)
82 Autopsy/ (43547)
83 autops*.tw,kf. (76636)
84 Death Certificates/ (5335)
85 (death? adj2 (certificat* or certify or certified or certifies)).tw,kf. (8077)
86 (death? adj2 review?).tw,kf. (1319)
87 exp Data Collection/ (2057062)
88 exp Epidemiologic Factors/ (1510721)
89 Epidemiological Monitoring/ (6374)
90 epidemiolog*.tw,kf. (386316)
91 ep.fs. [EPIDEMIOLOGY] (1593087)
92 (survey* or questionnaire*).tw,kf. (937634)
93 Incidence/ (249587)
94 incidenc*.tw,kf. (722400)
95 Prevalence/ (273020)
96 prevalen*.tw,kf. (682359)
97 exp Databases, Factual/ (120751)
98 ((clinical or disease* or factual) adj database*).tw,kf. (3073)
99 (register or registers or registry or registries).tw,kf. (167373)
100 sn.fs. [STATISTICS & NUMERICAL DATA] (741812)
101 surveillance*.tw,kf. (157712)
102 Survival Rate/ (170321)
103 Vital Statistics/ (5384)
104 (vital statistic* or vital registration?).tw,kf. (7747)
105 ((dead or death or deaths or decease* or demise* or die or died or dies or dying or fatal or fatality or fatalities or homicid* or mortal or mortality or mortalities or murder* or nonsurvival* or non-survival* or suicid*) adj3 (audit* or cause or causes or data or determinant* or estimat* or frequenc* or number or numbers or rate or rates or reason or reasons or report* or statistic* or trend*)).tw,kf. (378052)
106 (Reproductive Age Mortality Study or RAMOS).tw,kf. (1193)
107 (cause-specific mortality fraction* or CSMF).tw,kf. (113)
108 (confidential adj (inquir* or enquir*)).tw,kf. (652)
109 or/75-108 [MORTALITY STATISTICS/EPIDEMIOLGY] (5574833)
110 74 and 109 [DEATH/FATALITY/MORTALITY - PREGNANCY/MATERNAL POPULATION - EPIDEMIOLOGY] (93274)
111 Developing Countries.sh,kf. (86154)
112 (Africa? or Asia? or Caribbean or West Indies or South America? or Latin America? or Central America?).hw,kf,tw,cp. (504970)
113 (Afghanistan or Albania or Algeria or Angola or Antigua or Barbuda or Argentina or Armenia or Armenian or Aruba or Azerbaijan or Bahrain or Bangladesh or Barbados or Benin or Byelarus or Byelorussian or Belarus or Belorussian or Belorussia or Belize or

Bhutan or Bolivia or Bosnia or Herzegovina or Hercegovina or Botswana or Brasil or Brazil or "Brazzavilee" or Bulgaria or Burkina Faso or Burkina Fasso or Upper Volta or Burundi or Urundi or Cambodia or Khmer Republic or Kampuchea or Cameroon or Cameroons or Cameron or Camerons or "Canary Islands" or Cape Verde or Central African Republic or Chad or Chile or China or Colombia or Comoros or Comoro Islands or Comores or Mayotte or Congo or Zaire or Costa Rica or Cote d'Ivoire or Ivory Coast or Croatia or Cuba or Cyprus or Czechoslovakia or Czech Republic or Slovakia or Slovak Republic or Djibouti or French Somaliland or Dominica or Dominican Republic or East Timor or East Timur or Timor Leste or Ecuador or Egypt or United Arab Republic or El Salvador or Eritrea or Estonia or Ethiopia or Fiji or Gabon or Gabonese Republic or Gambia or Gaza or Georgia Republic or Georgian Republic or Ghana or Gold Coast or Greece or Grenada or Guatemala or Guinea or Guam or Guiana or Guyana or Haiti or Honduras or Hungary or India or Maldives or Indonesia or Iran or Iraq or Isle of Man or Jamaica or Jamahiriya? or Jamahiryia? or Jordan or Kazakhstan or Kazakh or Kenya or Kiribati or Korea or Kosovo or Kyrgyzstan or Kirghizia or Kyrgyz Republic or Kirghiz or Kirgizstan or Lao PDR or Laos or Latvia or Lebanon or Lesotho or Basutoland or Liberia or Libia or Libya or Lithuania or Macedonia or Madagascar or Maghreb or Maghrib or Malagasy Republic or Malaysia or Malaya or Malay or Mayote or Mocambique or Principe or Reunion or Sabah or Sarawak or Malawi or Nyasaland or Mali or Malta or Marshall Islands or Mauritania or Mauritius or Agalega Islands or Mexico or Micronesia or Middle East or Moldova or Moldovia or Moldovian or Mongolia or Montenegro or Morocco or Ifni or Mozambique or Myanmar or Myanma or Burma or Namibia or Nepal or Netherlands Antilles or New Caledonia or Nicaragua or Niger or Nigeria or Northern Mariana Islands or Oman or Muscat or Pakistan or Palau or Palestine or Panama or Paraguay or Peru or Philippines or Philipines or Phillipines or Phillippines or Poland or Portugal or Puerto Rico or Romania or Rumania or Roumania or Russia or Russian or Rwanda or Ruanda or Saint Kitts or St Kitts or Nevis or Saint Lucia or St Lucia or Saint Vincent or St Vincent or Grenadines or Samoa or Samoan Islands or Navigator Island or Navigator Islands or "St Helena" or "Saint Helena" or Sao Tome or Saudi Arabia or Senegal or Serbia or Montenegro or Seychelles or Sierra Leone or Slovenia or Sri Lanka or Ceylon or Solomon Islands or Somalia or South Africa or Sudan or Suriname or Surinam or Swaziland or Syria or Tajikistan or Tadzhikistan or Tadjikistan or Tadzhik or Tanzania or Thailand or Togo or Togolese Republic or Tonga or Trinidad or Tobago or Tunisia or Turkey or Turkmenistan or Turkmen or Uganda or Ukraine or Uruguay or USSR or Soviet Union or Union of Soviet Socialist Republics or Uzbekistan or Uzbek or Vanuatu or New Hebrides or Venezuela or Vietnam or Viet Nam or West Bank or "Western Sahara" or "Western Saharan" or Yemen or Yugoslavia or Zambia or Zimbabwe or Rhodesia).hw,kf,ti,ab,cp. (3459317)
114 ((developing or less* developed or under developed or underdeveloped or middle income or low* income or underserved or under served or deprived or poor*) adj (countr* or nation? or population? or world)).tw,kf. (117966)
115 ((developing or less* developed or under developed or underdeveloped or middle income or low* income) adj (economy or economies)).tw,kf. (464)
116 (low* adj (gdp or gnp or gross domestic or gross national)).tw,kf. (228)
117 (low adj3 middle adj3 countr*).tw,kf. (11083)
118 (lmic or lmics or third world or lami countr*).tw,kf. (5874)
119 transitional countr*.tw,kf. (160)
120 or/111-119 [LMICS] (3779263)
121 74 and 120 [DEATH/FATALITY/MORTALITY - PREGNANCY/MATERNAL POPULATION - LMICS] (40390)
122 110 or 121 [DEATH/FATALITY/MORTALITY - PREGNANCY/MATERNAL POPULATION - EPIDEMIOLOGY OR LMICS] (100758)
123 exp animals/ not (exp animals/ and humans/) (4805554)

124 122 not 123 [ANIMAL-ONLY REMOVED] (92540)
125 male/ not (male/ and female/) (2802556)
126 124 not 125 [MALE-ONLY REMOVED] (90836)
127 exp Child/ not (exp Child/ and exp Adult/) (1204619)
128 exp Infant/ not (exp Infant/ and exp Adult/) (840985)
129 126 not (127 or 128) [CHILD-ONLY REMOVED] (65089)
130 limit 129 to yr="2009-CURRENT" (28787)

Embase

Embase

1 maternal death/ (979)
2 (mortal or mortality or mortalities).tw,kw. (942656)
3 (dead or death or deaths or decease* or demise* or die or died or dies or dying).tw,kw. (1421008)
4 fatality/ (93559)
5 (fatal or fatality or fatalities).tw,kw. (185643)
6 (non-survival* or nonsurvival*).tw,kw. (1166)
7 "not surviv*".tw,kw. (6114)
8 suicide/ (52968)
9 suicid*.tw,kw. (86760)
10 homicide/ (16572)
11 (homicid* or kill or killed or kills or killing or murder*).tw,kw. (184374)
12 or/2-11 [DEATH/FATALITY/MORTALITY] (2452155)
13 exp pregnancy/ (732473)
14 exp pregnancy disorder/ (543358)
15 exp named groups by pregnancy/ (89660)
16 pregnan*.tw,kw. (624729)
17 birth/ (18243)
18 (parturition* or childbirth* or child birth*).tw,kw. (37466)
19 (gravidity or primigrav* or primi-grav* or multigrav* or multi-grav* or nulligrav* or nulli-grav* or parity or primipar* or primi-par* or multipar* or multi-par* or nullipar* or nulli-par*).tw,kw. (93448)
20 dystocia*.tw,kw. (5214)
21 ((uterin* or uterus or uteri*) adj1 inert*).tw,kw. (545)
22 ((labor* or labour*) adj3 induc*).tw,kw. (14421)
23 ((labor* or labour*) adj3 obstruct*).tw,kw. (1404)
24 ((labor* or labour*) adj3 (delayed or failed or false or prolong*)).tw,kw. (4146)
25 ((labor* or labour*) adj3 precipitate*).tw,kw. (206)
26 ((labor* or labour*) adj3 (birth* or deliver*)).tw,kw. (12779)
27 ((abdominal* or vaginal*) adj3 (birth* or deliver*)).tw,kw. (28303)
28 (breech adj3 (birth* or deliver* or extract*)).tw,kw. (2830)
29 ((forceps or instrumental or vacuum) adj3 (birth* or deliver* or extract*)).tw,kw. (8199)
30 ((premature* or pre-mature* or preterm* or pre-term*) adj3 (birth* or deliver* or labor* or labour*)).tw,kw. (60813)
31 ((premature* or pre-mature* or preterm* or pre-term*) adj3 (membran* adj2 ruptur*)).tw,kw. (6500)
32 (caesarean* or cesarean* or "c-section" or "c-sections" or post-caesarean* or post-cesarean*).tw,kw. (79217)
33 (VBAC or VBACs).tw,kw. (993)
34 (contract* adj3 (hypertonic or incoordinate* or prolonged)).tw,kw. (1555)
35 (episiotom* or (perineal adj1 lacerat*)).tw,kw. (4093)

36 ((hydatidiform or nonhydatidiform or non-hydatidiform) adj mole?).tw,kw. (4424)
37 ((multiple* or twin or twins or triplet?) adj gestation*).tw,kw. (5329)
38 exp maternal care/ (39811)
39 (maternal* or maternit* or mother*).tw,kw. (490875)
40 prenatal care/ (34830)
41 perinatal care/ (13180)
42 postnatal care/ (6326)
43 puerperium/ (38501)
44 perinatal period/ (30233)
45 prenatal period/ (9371)
46 (antenatal* or ante-natal* or antepartum* or ante-partum* or intrapartum* or intra-partum* or perinatal* or peri-natal* or peripartum* or peri-partum* or prenatal* or pre-natal* or postnatal* or post-natal* or postpartum* or post-partum* or puerperal* or puerperium).tw,kw. (411438)
47 (hyperemesis gravidarum or morning sickness*).tw,kw. (2067)
48 (gestation* adj2 diabet*).tw,kw. (18993)
49 (gestation* adj2 (edema* or hypertensi* or oedema* or proteinuria*)).tw,kw. (4618)
50 (eclamp* or pre-eclamp* or HELLP).tw,kw. (24387)
51 ((EPH or "Edema-Proteinuria-Hypertension" or "Hypertension-Edema-Proteinuria" or "Proteinuria-Edema-Hypertension") adj2 (complex* or gestos#s or tox?emi*)).tw,kw. (634)
52 (placenta* adj3 (disease* or disorder* or syndrome*)).tw,kw. (2310)
53 (placenta* adj3 (abrupti* or accreta* or previa* or retained)).tw,kw. (10828)
54 (placenta* adj3 insufficien*).tw,kw. (3206)
55 (placenta* adj3 (prematur* adj2 separat*)).tw,kw. (394)
56 (amnionit#s or chorioamnionit#s or funisit#s).tw,kw. (5869)
57 (amnio* adj3 infect*).tw,kw. (1560)
58 (amnio* adj3 emboli*).tw,kw. (1467)
59 (gestation$2 adj1 pemphigoid*).tw,kw. (379)
60 (hydramnios or oligohydramnios or polyhydramnios).tw,kw. (7176)
61 vasa previa.tw,kw. (281)
62 obstetric procedure/ (2103)
63 exp obstetric delivery/ (134487)
64 exp obstetric operation/ (152259)
65 obstetric*.tw,kw. (132386)
66 miscarr*.tw,kw. (20702)
67 abort*.tw,kw. (95257)
68 (postabort* or post-abort*).tw,kw. (2428)
69 exp abortive agent/ (160498)
70 ((dilatat* or dilat* or vacuum* or suction*) adj2 curettage*).tw,kw. (2891)
71 ((uterus or uteri or uterin* or vacuum*) adj2 aspirat*).tw,kw. (1353)
72 ((dilatat* or dilat*) adj2 evacuat*).tw,kw. (515)
73 or/13-72 [PREGNANCY/OBSTETRICS/MATERNAL POPULATION] (1823409)
74 12 and 73 (202314)
75 1 or 74 [DEATH/FATALITY/MORTALITY - PREGNANCY/OBSTETRICS/MATERNAL POPULATION] (202475)
76 maternal mortality/ (21470)
77 (maternal* adj3 mortalit*).tw,kw. (16915)
78 causality/ (1096)
79 "cause of death"/ (103140)
80 mortality/ (720221)
81 hospital mortality/ (10755)
82 autopsy/ (180564)

83 autops*.tw,kw. (116416)
84 death certificate/ (8375)
85 (death? adj2 (certificat* or certify or certified or certifies)).tw,kw. (9998)
86 epidemiological monitoring/ (1373)
87 epidemiological data/ (29875)
88 epidemiolog*.tw,kw. (480366)
89 ep.fs. [epidemiology] (985953)
90 exp health survey/ (197759)
91 exp questionnaire/ (577421)
92 (survey* or questionnaire*).tw,kw. (1181392)
93 exp incidence/ (364295)
94 incidenc*.tw,kw. (984808)
95 exp prevalence/ (601328)
96 prevalen*.tw,kw. (871292)
97 factual database/ (19697)
98 ((clinical or disease* or factual) adj database*).tw,kw. (4980)
99 (register or registers or registry or registries).tw,kw. (231860)
100 sentinel surveillance/ (2006)
101 surveillance*.tw,kw. (200896)
102 exp mortality rate/ (21015)
103 vital statistics/ (4831)
104 (vital statistic* or vital registration?).tw,kw. (5395)
105 ((dead or death or deaths or decease* or demise* or die or died or dies or dying or fatal or fatality or fatalities or homicid* or mortal or mortality or mortalities or murder* or nonsurvival* or non-survival* or suicid*) adj3 (audit* or cause or causes or data or determinant* or estimat* or frequenc* or number or numbers or rate or rates or reason or reasons or report* or statistic* or trend*)).tw,kw. (513729)
106 (Reproductive Age Mortality Study or RAMOS).tw,kw. (1795)
107 (cause-specific mortality fraction* or CSMF).tw,kw. (99)
108 (confidential adj (inquir* or enquir*)).tw,kw. (1032)
109 or/76-108 [MORTALITY STATISTICS/EPIDEMIOLGY] (4911611)
110 75 and 109 [DEATH/FATALITY/MORTALITY - PREGNANCY/MATERNAL POPULATION - EPIDEMIOLOGY] (113948)
111 Developing Country.sh,kw. (90016)
112 (Africa? or Asia? or Caribbean or West Indies or South America? or Latin America? or Central America?).hw,kw,tw,cp. (585643)
113 (Afghanistan or Albania or Algeria or Angola or Antigua or Barbuda or Argentina or Armenia or Armenian or Aruba or Azerbaijan or Bahrain or Bangladesh or Barbados or Benin or Byelarus or Byelorussian or Belarus or Belorussian or Belorussia or Belize or Bhutan or Bolivia or Bosnia or Herzegovina or Hercegovina or Botswana or Brasil or Brazil or "Brazzavilee" or Bulgaria or Burkina Faso or Burkina Fasso or Upper Volta or Burundi or Urundi or Cambodia or Khmer Republic or Kampuchea or Cameroon or Cameroons or Cameron or Camerons or "Canary Islands" or Cape Verde or Central African Republic or Chad or Chile or China or Colombia or Comoros or Comoro Islands or Comores or Mayotte or Congo or Zaire or Costa Rica or Cote d'Ivoire or Ivory Coast or Croatia or Cuba or Cyprus or Czechoslovakia or Czech Republic or Slovakia or Slovak Republic or Djibouti or French Somaliland or Dominica or Dominican Republic or East Timor or East Timur or Timor Leste or Ecuador or Egypt or United Arab Republic or El Salvador or Eritrea or Estonia or Ethiopia or Fiji or Gabon or Gabonese Republic or Gambia or Gaza or Georgia Republic or Georgian Republic or Ghana or Gold Coast or Greece or Grenada or Guatemala or Guinea or Guam or Guiana or Guyana or Haiti or Honduras or Hungary or India or Maldives or Indonesia or Iran or Iraq or Isle of Man or Jamaica or Jamahiriya? or

Jamahiryia? or Jordan or Kazakhstan or Kazakh or Kenya or Kiribati or Korea or Kosovo or Kyrgyzstan or Kirghizia or Kyrgyz Republic or Kirghiz or Kirgizstan or Lao PDR or Laos or Latvia or Lebanon or Lesotho or Basutoland or Liberia or Libia or Libya or Lithuania or Macedonia or Madagascar or Maghreb or Maghrib or Malagasy Republic or Malaysia or Malaya or Malay or Mayote or Mocambique or Principe or Reunion or Sabah or Sarawak or Malawi or Nyasaland or Mali or Malta or Marshall Islands or Mauritania or Mauritius or Agalega Islands or Mexico or Micronesia or Middle East or Moldova or Moldovia or Moldovian or Mongolia or Montenegro or Morocco or Ifni or Mozambique or Myanmar or Myanma or Burma or Namibia or Nepal or Netherlands Antilles or New Caledonia or Nicaragua or Niger or Nigeria or Northern Mariana Islands or Oman or Muscat or Pakistan or Palau or Palestine or Panama or Paraguay or Peru or Philippines or Philipines or Phillipines or Phillippines or Poland or Portugal or Puerto Rico or Romania or Rumania or Roumania or Russia or Russian or Rwanda or Ruanda or Saint Kitts or St Kitts or Nevis or Saint Lucia or St Lucia or Saint Vincent or St Vincent or Grenadines or Samoa or Samoan Islands or Navigator Island or Navigator Islands or "St Helena" or "Saint Helena" or Sao Tome or Saudi Arabia or Senegal or Serbia or Montenegro or Seychelles or Sierra Leone or Slovenia or Sri Lanka or Ceylon or Solomon Islands or Somalia or South Africa or Sudan or Suriname or Surinam or Swaziland or Syria or Tajikistan or Tadzhikistan or Tadjikistan or Tadzhik or Tanzania or Thailand or Togo or Togolese Republic or Tonga or Trinidad or Tobago or Tunisia or Turkey or Turkmenistan or Turkmen or Uganda or Ukraine or Uruguay or USSR or Soviet Union or Union of Soviet Socialist Republics or Uzbekistan or Uzbek or Vanuatu or New Hebrides or Venezuela or Vietnam or Viet Nam or West Bank or "Western Sahara" or "Western Saharan" or Yemen or Yugoslavia or Zambia or Zimbabwe or Rhodesia).hw,kw,tw,cp. (3875288)
114 ((developing or less* developed or under developed or underdeveloped or middle income or low* income or underserved or under served or deprived or poor*) adj (countr* or nation? or population? or world)).tw,kw. (104532)
115 ((developing or less* developed or under developed or underdeveloped or middle income or low* income) adj (economy or economies)).tw,kw. (546)
116 (low* adj (gdp or gnp or gross domestic or gross national)).tw,kw. (306)
117 (low adj3 middle adj3 countr*).tw,kw. (11371)
118 (lmic or lmics or third world or lami countr*).tw,kw. (6435)
119 transitional countr*.tw,kw. (202)
120 or/111-119 [LMICs] (4280114)
121 75 and 120 [DEATH/FATALITY/MORTALITY - PREGNANCY/MATERNAL POPULATION - LMICS] (46646)
122 110 or 121 [DEATH/FATALITY/MORTALITY - PREGNANCY/MATERNAL POPULATION - EPIDEMIOLOGY OR LMICS] (126333)
123 exp animal experimentation/ or exp animal model/ or exp animal experiment/ or nonhuman/ or exp vertebrate/ (25461025)
124 exp human/ or exp human experimentation/ or exp human experiment/ (19377434)
125 123 not 124 (6084659)
126 122 not 125 [ANIMAL-ONLY REMOVED] (116481)
127 male/ not (male/ and female/) (2606348)
128 126 not 127 [MALE-ONLY REMOVED] (113516)
129 exp child/ not (exp child/ and exp adult/) (1985986)
130 exp infant/ not (exp infant/ and exp adult/) (837365)
131 fetus/ not (fetus/ and exp adult/) (169009)
132 128 not (129 or 130 or 131) [CHILD, FETUS-ONLY REMOVED] (75773)
133 limit 132 to yr="2009-CURRENT" (40861)
134 conference abstract.pt. (2877925)
135 133 not 134 [CONFERENCE ABSTRACTS REMOVED] (28567)

136 exp child/ not (exp adult/ or adolescent/) (1665948)
137 exp infant/ not (exp adult/ or adolescent/) (762021)
138 fetus/ not (exp adult/ or adolescent/) (166942)
139 126 not (136 or 137 or 138) (78668)
140 limit 139 to yr="2009-CURRENT" (42721)
141 140 not 134 (29509)
142 ("20171217" or "20171218" or "20171219" or 2017122* or 2017123* or 2018*).dc. (273145)
143 135 not 142 [UPDATE PERIOD EXCLUDED] (27943)
144 141 not 142 [UPDATE PERIOD EXCLUDED] (28867)
145 144 not 143 [ADOLESCENT RECORDS, RECOVERED] (924)

Popline

CONTROLLED VOCABULARY:
Maternal Mortality OR Mortality OR Suicide OR Homicide OR Death

OR

ALL FIELDS:
mortal OR mortality OR mortalities OR dead OR death OR deaths OR decease* OR demise* OR die OR died OR dies OR dying OR fatal OR fatality OR fatalities OR non-survival* OR nonsurvival* OR suicid* OR homicid* OR kill OR killed OR kills OR killing OR murder*

AND

CONTROLLED VOCABULARY:
Pregnancy OR Adolescent Vocabulary OR Pregnancy Abdominal OR Pregnancy Complications OR Pregnancy Ectopic OR Pregnancy First Trimester OR Pregnancy High Risk OR Pregnancy Ovarian OR Pregnancy Prolonged OR Pregnancy Second Trimester OR Pregnancy Third Trimester OR Pregnancy Tubal OR Pregnancy Unplanned OR Pregnancy Unwanted OR Pregnant Women OR Maternal Health OR Postpartum OR Postpartum Women OR Obstetrical Surgery OR Abortion OR Habitual Spontaneous Abortion OR Postabortion OR Postabortion Care OR Spontaneous Abortion OR Preeclampsia OR Eclampsia OR Cesarean Section OR Post-Cesarean Section OR Childbirth OR Puerperium OR Maternal Health Services OR Forceps OR Parity OR Multiparity or Nulliparity OR Multiple Birth OR Premature Labor OR Premature Birth OR Curettage OR Maternal Mortality

OR
ALL FIELDS:

pregnan* OR parturition* OR childbirth* OR "child birth" OR "child births" OR dystocia* OR caesarean* OR cesarean* OR "c-section" OR "c-sections" OR maternal* OR maternit* OR mother* OR antenatal* OR ante-natal* OR antepartum OR ante-partum* OR perinatal* OR peri-natal* OR peripartum* OR peri-partum* OR prenatal* OR pre-natal* OR postnatal* OR post-natal* OR puerperal* OR puerperium OR eclamp* OR pre-eclamp* OR
HELLP OR obstetric* OR abort* OR miscarr* OR postabort* OR post-abort* OR curettage* OR postcesarean* OR post-cesarean* OR postcaesarean* OR post-caesarean* OR VBAC OR VBACs OR "hyperemesis gravidarum" OR "morning sickness" OR "gestational diabetes" OR "gestational edema" OR "gestational oedema" OR "gestational proteinuria" OR amnionitis OR chorioamnionitis OR funisitis OR hydramnios OR oligohydramnios OR

polyhydramnios OR "vasa previa" OR parity OR primipar* OR primi-par* OR multipar* OR multi-par* OR nullipar* OR nulli-par* OR gravidity OR primigrav* OR primi-grav* OR multigrav* OR multi-grav* OR nulligrav* OR nulli-grav* OR gestational hypertension

OR

(abdominal* OR breech OR forceps OR instrumental* or vaginal* OR vacuum*) AND (birth* OR deliver* OR extract*)

OR

"labor induce" ~3 OR "labor induced" ~3 OR "labor induces" ~3 OR "labor inducing" ~3 OR "labor induction" ~3 OR "labor inductions" ~3 OR "labour induce" ~3 OR "labour induced" ~3 OR "labour induces" ~3 OR "labour inducing" ~3 OR "labour induction" ~3 OR "labour inductions" ~3 OR "labors induce" ~3 OR "labors induced" ~3 OR "labors induces" ~3 OR "labors inducing" ~3 OR "labors induction" ~3 OR "labors inductions" ~3 OR "labours induce" ~3 OR "labours induced" ~3 OR "labours induces" ~3 OR "labours inducing" ~3 OR "labours induction" ~3 OR "labours inductions" ~3

OR

"obstructed labor" ~3 OR "obstruction labor" ~3 OR "obstructions labor" ~3 OR "obstructing labor" ~3 OR "obstructs labor" ~3 OR "obstruct labour" ~3 OR "obstructed labour" ~3 OR "obstruction labour" ~3 OR "obstructions labour" ~3 OR "obstructing labour" ~3 OR "obstructs labour" ~3 OR "obstruct labour" ~3 OR "obstructed labors" ~3 OR "obstruction labors" ~3 OR "obstructions labors" ~3 OR "obstructing labors" ~3 OR "obstructs labors" ~3 OR "obstruct labours" ~3 OR "obstructed labours" ~3 OR "obstruction labours" ~3 OR "obstructions labours" ~3 OR "obstructing labours" ~3 OR "obstructs labours" ~3 OR "obstruct labours" ~3

OR

"prolonged contraction" ~3 OR "prolongation contraction" ~3 OR "prolongations contraction" ~3 OR "prolonging contraction" ~3 OR "prolongs contraction" ~3 OR "prolong contraction" ~3 OR "prolonged contractions" ~3 OR "prolongation contractions" ~3 OR "prolongations contractions" ~3 OR "prolonging contractions" ~3 OR "prolongs contractions" ~3 OR "prolong contractions" ~3

OR

"prolonged labor" ~3 OR "prolongation labor" ~3 OR "prolongations labor" ~3 OR "prolonging labor" ~3 OR "prolongs labor" ~3 OR "prolong labour" ~3 OR "prolonged labour" ~3 OR "prolongation labour" ~3 OR "prolongations labour" ~3 OR "prolonging labour" ~3 OR "prolongs labour" ~3 OR "prolong labour" ~3 OR "prolonged labors" ~3 OR "prolongation labors" ~3 OR "prolongations labors" ~3 OR "prolonging labors" ~3 OR "prolongs labors" ~3 OR "prolong labours" ~3 OR "prolonged labours" ~3 OR "prolongation labours" ~3 OR "prolongations labours" ~3 OR "prolonging labours" ~3 OR "prolongs labours" ~3 OR "prolong labours" ~3

OR

"failed labor" ~3 OR "failed labors" ~3 OR "failed labour" OR "failed labours" ~3 OR "precipitate labor" ~3 OR "precipitates labor" ~3 OR "precipitating labor" ~3 OR "precipitated labor" ~3 OR "precipitate labour" ~3 OR "precipitates labour" ~3 OR "precipitating labour" ~3 OR "precipitated labour" ~3 OR "precipitate labors" ~3 OR "precipitates labors" ~3 OR "precipitating labors" ~3 OR "precipitated labors" ~3 OR "precipitate labours" ~3 OR "precipitates labours" ~3 OR "precipitating labours" ~3 OR "precipitated labours" ~3

OR

"placental disease" ~3 OR "placental disorder" ~3 OR "placental syndrome" ~3 OR "placental diseases" ~3 OR "placental disorders" ~3 OR "placental syndromes" ~3 OR "placentae disease" ~3 OR "placentae disorder" ~3 OR "placentae syndrome" ~3 OR "placentae diseases" ~3 OR "placentae disorders" ~3 OR "placentae syndromes" ~3 OR "placental abruption" ~3 OR "placental accreta" ~3 OR "placental previa" ~3 OR "placental retained" ~3 OR "placental abruptions" ~3 OR "placental accretas" ~3 OR "placental previas" ~3 OR "placentae abruption" ~3 OR "placentae accreta" ~3 OR "placentae previa" ~3 OR "placentae retained" ~3 OR "placentae abruptions" ~3 OR "placentae accretas" ~3 OR "placentae previas" ~3 OR "placenta abruptio" ~3 OR "placentae abruptio" ~3 OR "placental insufficiency" ~3 OR "placental insufficiencies" ~3 OR "placentae insufficiency" ~3 OR "placentae insufficiencies" ~3 OR "amnion infection" ~3 OR "amnion infections" ~3 OR "amniotic infection" ~3 OR "amniotic infections" ~3 OR "amnion embolism" ~3 OR "amnion embolisms" ~3 OR "amniotic embolism" ~3 OR "amniotic embolisms" ~3 OR "gestational pemphigoid" ~3 OR "gestationis pemphigoid" ~3

OR

"uterine rupture" ~3 OR "uterine ruptured" ~3 OR "multiple gestation" ~3 OR "multiple gestations" ~3 OR "twin gestation" ~3 OR "twin gestations" ~3 OR "triplet gestation" ~3 OR "triplet gestations" ~3

OR

"premature rupture of membranes" OR "rupture membranes prematurely" OR "premature birth" ~3 OR "premature births" ~3 OR "pre-mature birth" ~3 OR "pre-mature births" ~3 OR "preterm birth" ~3 OR "preterm births" ~3 OR "pre-term birth" ~3 OR "pre-term births" ~3 OR "premature delivery" ~3 OR "premature deliveries" ~3 OR "pre-mature delivery" ~3 OR "pre-mature deliveries" ~3 OR "preterm delivery" ~3 OR "preterm deliveries" ~3 OR "pre-term delivery" ~3 OR "pre-term deliveries" ~3

AND

CONTROLLED VOCABULARY:
Autopsy OR Data Collection OR Health Surveys OR Incidence OR Prevalence OR Vital Statistics OR Epidemiology OR Causes of Death OR Mortality Changes OR Mortality Decline OR Mortality Determinants OR Death Rate OR Death Records OR Age Specific Death Rate

OR

ALL FIELDS:

autops* OR causality OR "death certificate" OR "death certificates" OR "certificate of death" OR "death review" OR "data collection" OR "data collections" OR epidemiolog* OR incidenc* OR prevalen* OR statistic OR statistics OR statistical OR surveillance* OR survey OR surveys OR questionnaire* OR rate OR rates OR "vital statistics" OR register OR registers OR registry OR registries OR "clinical database" OR "clinical databases" OR "disease database" OR "disease databases" OR "factual database" OR "factual databases" OR "Reproductive Age Mortality Study" OR RAMOS OR "cause-specific mortality fraction" OR CSMF OR "cause death" ~3 OR "causes death" ~3 OR "caused death" ~3 OR "caused deaths" ~3 OR "causing death" ~3 OR "causing deaths" ~3 OR "cause mortality" ~3 OR "causes mortalities" ~3 OR "caused mortality" ~3 OR "caused mortalities" ~3 OR "causing mortality" ~3 OR "causing mortalities" ~3 OR "reason death" ~3 OR "reasons death" ~3 OR "reasons death" ~3 OR "reasons deaths" ~3 OR "number death" ~3 OR "numbers deaths" ~3 OR "number fatality" ~3 OR "number fatalities" OR "numbers fatality" OR "numbers fatalities" ~3 OR "number mortality" ~3 OR "number mortalities" OR "numbers mortality" OR "numbers mortalities" ~3 OR "report death" ~3 OR "report deaths" ~3 OR "reports death" ~3 OR "reports deaths" ~3 OR "reporting death" ~3 OR "reporting deaths" ~3 OR "reported death" ~3 OR "reported deaths" ~3 OR "report fatality" ~3 OR "report fatalities" ~3 OR "reports fatality" ~3 OR "reports fatalities" ~3 OR "reporting fatality" ~3 OR "reporting fatalities" ~3 OR "reported fatality" ~3 OR "reported fatalities" ~3 OR "report mortality" ~3 OR "report mortalities" ~3 OR "reports mortality" ~3 OR "reports mortalities" ~3 OR "reporting mortality" ~3 OR "reporting mortalities" ~3 OR "reported mortality" ~3 OR "reported mortalities" ~3 OR "death registration" ~3 OR "deaths registration" ~3 OR "death registrations" ~3 OR "deaths registrations" ~3 OR "confidential inquiry" OR "confidential inquiries" OR "confidential enquiry" OR "confidential enquiries"

OR

"maternal mortality" ~3 OR "maternal mortalities" ~3 OR "survival rate" ~3 OR "survival rates" ~3 OR "audit death" ~3 OR "audit deaths" ~3 OR "audits death" ~3 OR "audits deaths" ~3 OR "auditing death"~3 OR "auditing deaths" ~3 OR "audited death" ~3 OR "audited deaths" ~3 OR "determinant death" ~3 OR "determinant deaths" ~3 OR "determinants death" ~3 OR "determinants deaths" ~3 OR "estimate death" ~3 OR "estimate deaths" ~3 OR "estimates death" ~3 OR "estimates deaths" ~3 OR "estimated death" ~3 OR "estimated deaths" ~3 OR "frequency death" ~3 OR "frequency deaths" ~3 OR "frequencies death" ~3 OR "frequencies deaths" ~3 OR "death trend" ~3 OR "death trends" ~3 OR "deaths trend" ~3 OR "deaths trends" ~3 OR "mortality trend"~3 OR "mortality trends" ~3 OR "mortalities trend" ~3 OR "mortalities trends" ~3

Popline – LMIC – Pt 1
2876 records

CONTROLLED VOCABULARY:
Maternal Mortality OR Mortality OR Suicide OR Homicide OR Death

OR

ALL FIELDS:
mortal OR mortality OR mortalities OR dead OR death OR deaths OR decease* OR demise* OR die OR died OR dies OR dying OR fatal OR fatality OR fatalities OR non-survival* OR nonsurvival* OR suicid* OR homicid* OR kill OR killed OR kills OR killing OR murder*

AND

CONTROLLED VOCABULARY:
Pregnancy OR Adolescent Vocabulary OR Pregnancy Abdominal OR Pregnancy Complications OR Pregnancy Ectopic OR Pregnancy First Trimester OR Pregnancy High Risk OR Pregnancy Ovarian OR Pregnancy Prolonged OR Pregnancy Second Trimester OR Pregnancy Third Trimester OR Pregnancy Tubal OR Pregnancy Unplanned OR Pregnancy Unwanted OR Pregnant Women OR Maternal Health OR Postpartum OR Postpartum Women OR Obstetrical Surgery OR Abortion OR Habitual Spontaneous Abortion OR Postabortion OR Postabortion Care OR Spontaneous Abortion OR Preeclampsia OR Eclampsia OR Cesarean Section OR Post-Cesarean Section OR Childbirth OR Puerperium OR Maternal Health Services OR Forceps OR Parity OR Multiparity or Nulliparity OR Multiple Birth OR Premature Labor OR Premature Birth OR Curettage OR Maternal Mortality

OR
ALL FIELDS:

pregnan* OR parturition* OR childbirth* OR "child birth" OR "child births" OR dystocia* OR caesarean* OR cesarean* OR "c-section" OR "c-sections" OR maternal* OR maternit* OR mother* OR antenatal* OR ante-natal* OR antepartum OR ante-partum* OR perinatal* OR peri-natal* OR peripartum* OR peri-partum* OR prenatal* OR pre-natal* OR postnatal* OR post-natal* OR puerperal* OR puerperium OR eclamp* OR pre-eclamp* OR HELLP OR obstetric* OR abort* OR miscarr* OR postabort* OR post-abort* OR curettage* OR postcesarean* OR post-cesarean* OR postcaesarean* OR post-caesarean* OR VBAC OR VBACs OR "hyperemesis gravidarum" OR "morning sickness" OR "gestational diabetes" OR "gestational edema" OR "gestational oedema" OR "gestational proteinuria" OR amnionitis OR chorioamnionitis OR funisitis OR hydramnios OR oligohydramnios OR polyhydramnios OR "vasa previa" OR parity OR primipar* OR primi-par* OR multipar* OR multi-par* OR nullipar* OR nulli-par* OR gravidity OR primigrav* OR primi-grav* OR multigrav* OR multi-grav* OR nulligrav* OR nulli-grav* OR gestational hypertension

OR

(abdominal* OR breech OR forceps OR instrumental* or vaginal* OR vacuum*) AND (birth* OR deliver* OR extract*)

OR

"labor induce" ~3 OR "labor induced" ~3 OR "labor induces" ~3 OR "labor inducing" ~3 OR "labor induction" ~3 OR "labor inductions" ~3 OR "labour induce" ~3 OR "labour induced" ~3 OR "labour induces" ~3 OR "labour inducing" ~3 OR "labour induction" ~3 OR "labour inductions" ~3 OR "labors induce" ~3 OR "labors induced" ~3 OR "labors induces" ~3 OR "labors inducing" ~3 OR "labors induction" ~3 OR "labors inductions" ~3 OR "labours induce" ~3 OR "labours induced" ~3 OR "labours induces" ~3 OR "labours inducing" ~3 OR "labours induction" ~3 OR "labours inductions" ~3

OR

"obstructed labor" ~3 OR "obstruction labor" ~3 OR "obstructions labor" ~3 OR "obstructing labor" ~3 OR "obstructs labor" ~3 OR "obstruct labour" ~3 OR "obstructed labour" ~3 OR "obstruction labour" ~3 OR "obstructions labour" ~3 OR "obstructing labour" ~3 OR

"obstructs labour" ~3 OR "obstruct labour" ~3 OR "obstructed labors" ~3 OR "obstruction labors" ~3 OR "obstructions labors" ~3 OR "obstructing labors" ~3 OR "obstructs labors" ~3 OR "obstruct labours" ~3 OR "obstructed labours" ~3 OR "obstruction labours" ~3 OR "obstructions labours" ~3 OR "obstructing labours" ~3 OR "obstructs labours" ~3 OR "obstruct labours" ~3

OR

"prolonged contraction" ~3 OR "prolongation contraction" ~3 OR "prolongations contraction" ~3 OR "prolonging contraction" ~3 OR "prolongs contraction" ~3 OR "prolong contraction" ~3 OR "prolonged contractions" ~3 OR "prolongation contractions" ~3 OR "prolongations contractions" ~3 OR "prolonging contractions" ~3 OR "prolongs contractions" ~3 OR "prolong contractions" ~3

OR

"prolonged labor" ~3 OR "prolongation labor" ~3 OR "prolongations labor" ~3 OR "prolonging labor" ~3 OR "prolongs labor" ~3 OR "prolong labour" ~3 OR "prolonged labour" ~3 OR "prolongation labour" ~3 OR "prolongations labour" ~3 OR "prolonging labour" ~3 OR "prolongs labour" ~3 OR "prolong labour" ~3 OR "prolonged labors" ~3 OR "prolongation labors" ~3 OR "prolongations labors" ~3 OR "prolonging labors" ~3 OR "prolongs labors" ~3 OR "prolong labours" ~3 OR "prolonged labours" ~3 OR "prolongation labours" ~3 OR "prolongations labours" ~3 OR "prolonging labours" ~3 OR "prolongs labours" ~3 OR "prolong labours" ~3

OR

"failed labor" ~3 OR "failed labors" ~3 OR "failed labour" OR "failed labours" ~3 OR "precipitate labor" ~3 OR "precipitates labor" ~3 OR "precipitating labor" ~3 OR "precipitated labor" ~3 OR "precipitate labour" ~3 OR "precipitates labour" ~3 OR "precipitating labour" ~3 OR "precipitated labour" ~3 OR "precipitate labors" ~3 OR "precipitates labors" ~3 OR "precipitating labors" ~3 OR "precipitated labors" ~3 OR "precipitate labours" ~3 OR "precipitates labours" ~3 OR "precipitating labours" ~3 OR "precipitated labours" ~3

OR

"placental disease" ~3 OR "placental disorder" ~3 OR "placental syndrome" ~3 OR "placental diseases" ~3 OR "placental disorders" ~3 OR "placental syndromes" ~3 OR "placentae disease" ~3 OR "placentae disorder" ~3 OR "placentae syndrome" ~3 OR "placentae diseases" ~3 OR "placentae disorders" ~3 OR "placentae syndromes" ~3 OR "placental abruption" ~3 OR "placental accreta" ~3 OR "placental previa" ~3 OR "placental retained" ~3 OR "placental abruptions" ~3 OR "placental accretas" ~3 OR "placental previas" ~3 OR "placentae abruption" ~3 OR "placentae accreta" ~3 OR "placentae previa" ~3 OR "placentae retained" ~3 OR "placentae abruptions" ~3 OR "placentae accretas" ~3 OR "placentae previas" ~3 OR "placenta abruptio" ~3 OR "placentae abruptio" ~3 OR "placental insufficiency" ~3 OR "placental insufficiencies" ~3 OR "placentae insufficiency" ~3 OR "placentae insufficiencies" ~3 OR "amnion infection" ~3 OR "amnion infections" ~3 OR "amniotic infection" ~3 OR "amniotic infections" ~3 OR "amnion embolism" ~3 OR "amnion embolisms" ~3 OR "amniotic embolism" ~3 OR "amniotic embolisms" ~3 OR "gestational pemphigoid" ~3 OR "gestationis pemphigoid" ~3

OR

"uterine rupture" ~3 OR "uterine ruptured" ~3 OR "multiple gestation" ~3 OR "multiple gestations" ~3 OR "twin gestation" ~3 OR "twin gestations" ~3 OR "triplet gestation" ~3 OR "triplet gestations" ~3

OR

"premature rupture of membranes" OR "rupture membranes prematurely" OR "premature birth" ~3 OR "premature births" ~3 OR "pre-mature birth" ~3 OR "pre-mature births" ~3 OR "preterm birth" ~3 OR "preterm births" ~3 OR "pre-term birth" ~3 OR "pre-term births" ~3 OR "premature delivery" ~3 OR "premature deliveries" ~3 OR "pre-mature delivery" ~3 OR "pre-mature deliveries" ~3 OR "preterm delivery" ~3 OR "preterm deliveries" ~3 OR "pre-term delivery" ~3 OR "pre-term deliveries" ~3

AND

CONTROLLED VOCABULARY:
Africa OR Africa Central OR Africa Eastern OR Africa North OR Africa Southern OR Africa Sub Saharan
OR Africa Western

OR

Algeria OR Angola OR Benin OR Botswana OR Burkina Faso OR Burundi OR Cameroon OR Cape Verde OR
Central African Republic OR Chad OR Comoros OR Democratic Republic of the Congo OR Republic of the Congo OR Djibouti OR Egypt OR Equatorial Guinea OR Eritrea OR Ethiopia

OR

Gabon OR Gambia OR Ghana OR Guinea OR Guinea-Bissau OR Cote D'ivoire OR Kenya OR Lesotho OR Liberia OR Libya OR Madagascar OR Malawi OR Mali OR Mauritania OR Mauritius OR Morocco OR × Mozambique

OR

Namibia OR Niger OR Nigeria OR Reunion OR Rwanda OR Sao Tome and Principe OR Senegal OR Seychelles OR Sierra Leone OR Somalia OR South Africa OR South Sudan OR Sudan OR Saint Helena OR
Swaziland OR Tanzania OR Togo OR Tunisia OR Uganda OR Zambia OR Zimbabwe

OR

Asia OR Afghanistan OR Armenia OR Azerbaijan OR Bahrain OR Bangladesh OR Bhutan OR Cambodia OR China OR Cyprus OR Georgia OR India OR Indonesia OR Iran OR Iraq OR Jordan OR Kazakhstan OR Kyrgyzstan OR Laos OR Lebanon OR Malaysia OR Maldives OR Mongolia OR Myanmar OR Nepal OR Democratic People's Republic of Korea OR Oman OR Pakistan OR Philippines OR Russia OR Saudi Arabia OR Sri Lanka OR Syria OR Tajikistan OR Thailand OR Timor-Leste OR Turkey OR Turkmenistan OR Uzbekistan OR Vietnam OR Yemen

OR

Central America OR Belize OR Costa Rica OR El Salvador OR Guatemala OR Honduras OR Nicaragua OR Panama

OR

South America OR Argentina OR Brazil OR Bolivia OR Chile OR Colombia OR Ecuador OR Guyana OR Paraguay OR Peru OR Suriname OR Uruguay OR Venezuela

OR

Caribbean OR Anguilla OR Antigua and Barbuda OR Barbados OR Cuba OR Dominica OR Dominican Republic OR Grenada OR Haiti OR Jamaica OR Netherlands Antilles OR Puerto Rico OR Saint Kitts and Nevis OR Saint Lucia OR Saint Vincent and The Grenadines OR Trinidad and Tobago

OR

Oceania OR Fiji OR Kiribati OR Marshall Islands OR Federated States of Micronesia OR Papua New Guinea OR Samoa OR American Samoa OR Solomon Islands OR Tonga OR Tuvalu OR Vanuatu

OR

Belarus OR Bosnia and Herzegovina OR Bulgaria OR Macedonia OR Mexico OR Mongolia OR Serbia and Montenegro OR Romania OR Turkey OR Ukraine
OR West Bank

Popline – LMIC – Pt 2
2851 records

CONTROLLED VOCABULARY:
Maternal Mortality OR Mortality OR Suicide OR Homicide OR Death

OR

ALL FIELDS:
mortal OR mortality OR mortalities OR dead OR death OR deaths OR decease* OR demise* OR die OR died OR dies OR dying OR fatal OR fatality OR fatalities OR non-survival* OR nonsurvival* OR suicid* OR homicid* OR kill OR killed OR kills OR killing OR murder*

AND

CONTROLLED VOCABULARY:
Pregnancy OR Adolescent Vocabulary OR Pregnancy Abdominal OR Pregnancy Complications OR Pregnancy Ectopic OR Pregnancy First Trimester OR Pregnancy High Risk OR Pregnancy Ovarian OR Pregnancy Prolonged OR Pregnancy Second Trimester OR Pregnancy Third Trimester OR Pregnancy Tubal OR Pregnancy Unplanned OR Pregnancy Unwanted OR Pregnant Women OR Maternal Health OR Postpartum OR Postpartum Women OR Obstetrical Surgery OR Abortion OR Habitual Spontaneous

Abortion OR Postabortion OR Postabortion Care OR Spontaneous Abortion OR Preeclampsia OR Eclampsia OR Cesarean Section OR Post-Cesarean Section OR Childbirth OR Puerperium OR Maternal Health Services OR Forceps OR Parity OR Multiparity or Nulliparity OR Multiple Birth OR Premature Labor OR Premature Birth OR Curettage OR Maternal Mortality

OR
ALL FIELDS:

pregnan* OR parturition* OR childbirth* OR "child birth" OR "child births" OR dystocia* OR caesarean* OR cesarean* OR "c-section" OR "c-sections" OR maternal* OR maternit* OR mother* OR antenatal* OR ante-natal* OR antepartum OR ante-partum* OR perinatal* OR peri-natal* OR peripartum* OR peri-partum* OR prenatal* OR pre-natal* OR postnatal* OR post-natal* OR puerperal* OR puerperium OR eclamp* OR pre-eclamp* OR HELLP OR obstetric* OR abort* OR miscarr* OR postabort* OR post-abort* OR curettage* OR postcesarean* OR post-cesarean* OR postcaesarean* OR post-caesarean* OR VBAC OR VBACs OR "hyperemesis gravidarum" OR "morning sickness" OR "gestational diabetes" OR "gestational edema" OR "gestational oedema" OR "gestational proteinuria" OR amnionitis OR chorioamnionitis OR funisitis OR hydramnios OR oligohydramnios OR polyhydramnios OR "vasa previa" OR parity OR primipar* OR primi-par* OR multipar* OR multi-par* OR nullipar* OR nulli-par* OR gravidity OR primigrav* OR primi-grav* OR multigrav* OR multi-grav* OR nulligrav* OR nulli-grav* OR gestational hypertension

OR

(abdominal* OR breech OR forceps OR instrumental* or vaginal* OR vacuum*) AND (birth* OR deliver* OR extract*)

OR

"labor induce" ~3 OR "labor induced" ~3 OR "labor induces" ~3 OR "labor inducing" ~3 OR "labor induction" ~3 OR "labor inductions" ~3 OR "labour induce" ~3 OR "labour induced" ~3 OR "labour induces" ~3 OR "labour inducing" ~3 OR "labour induction" ~3 OR "labour inductions" ~3 OR "labors induce" ~3 OR "labors induced" ~3 OR "labors induces" ~3 OR "labors inducing" ~3 OR "labors induction" ~3 OR "labors inductions" ~3 OR "labours induce" ~3 OR "labours induced" ~3 OR "labours induces" ~3 OR "labours inducing" ~3 OR "labours induction" ~3 OR "labours inductions" ~3

OR

"obstructed labor" ~3 OR "obstruction labor" ~3 OR "obstructions labor" ~3 OR "obstructing labor" ~3 OR "obstructs labor" ~3 OR "obstruct labour" ~3 OR "obstructed labour" ~3 OR "obstruction labour" ~3 OR "obstructions labour" ~3 OR "obstructing labour" ~3 OR "obstructs labour" ~3 OR "obstruct labour" ~3 OR "obstructed labors" ~3 OR "obstruction labors" ~3 OR "obstructions labors" ~3 OR "obstructing labors" ~3 OR "obstructs labors" ~3 OR "obstruct labours" ~3 OR "obstructed labours" ~3 OR "obstruction labours" ~3 OR "obstructions labours" ~3 OR "obstructing labours" ~3 OR "obstructs labours" ~3 OR "obstruct labours" ~3

OR

"prolonged contraction" ~3 OR "prolongation contraction" ~3 OR "prolongations contraction" ~3 OR "prolonging contraction" ~3 OR "prolongs contraction" ~3 OR "prolong contraction" ~3 OR "prolonged contractions" ~3 OR "prolongation contractions" ~3 OR "prolongations contractions" ~3 OR "prolonging contractions" ~3 OR "prolongs contractions" ~3 OR "prolong contractions" ~3

OR

"prolonged labor" ~3 OR "prolongation labor" ~3 OR "prolongations labor" ~3 OR "prolonging labor" ~3 OR "prolongs labor" ~3 OR "prolong labour" ~3 OR "prolonged labour" ~3 OR "prolongation labour" ~3 OR "prolongations labour" ~3 OR "prolonging labour" ~3 OR "prolongs labour" ~3 OR "prolong labour" ~3 OR "prolonged labors" ~3 OR "prolongation labors" ~3 OR "prolongations labors" ~3 OR "prolonging labors" ~3 OR "prolongs labors" ~3 OR "prolong labours" ~3 OR "prolonged labours" ~3 OR "prolongation labours" ~3 OR "prolongations labours" ~3 OR "prolonging labours" ~3 OR "prolongs labours" ~3 OR "prolong labours" ~3

OR

"failed labor" ~3 OR "failed labors" ~3 OR "failed labour" OR "failed labours" ~3 OR "precipitate labor" ~3 OR "precipitates labor" ~3 OR "precipitating labor" ~3 OR "precipitated labor" ~3 OR "precipitate labour" ~3 OR "precipitates labour" ~3 OR "precipitating labour" ~3 OR "precipitated labour" ~3 OR "precipitate labors" ~3 OR "precipitates labors" ~3 OR "precipitating labors" ~3 OR "precipitated labors" ~3 OR "precipitate labours" ~3 OR "precipitates labours" ~3 OR "precipitating labours" ~3 OR "precipitated labours" ~3

OR

"placental disease" ~3 OR "placental disorder" ~3 OR "placental syndrome" ~3 OR "placental diseases" ~3 OR "placental disorders" ~3 OR "placental syndromes" ~3 OR "placentae disease" ~3 OR "placentae disorder" ~3 OR "placentae syndrome" ~3 OR "placentae diseases" ~3 OR "placentae disorders" ~3 OR "placentae syndromes" ~3 OR "placental abruption" ~3 OR "placental accreta" ~3 OR "placental previa" ~3 OR "placental retained" ~3 OR "placental abruptions" ~3 OR "placental accretas" ~3 OR "placental previas" ~3 OR "placentae abruption" ~3 OR "placentae accreta" ~3 OR "placentae previa" ~3 OR "placentae retained" ~3 OR "placentae abruptions" ~3 OR "placentae accretas" ~3 OR "placentae previas" ~3 OR "placenta abruptio" ~3 OR "placentae abruptio" ~3 OR "placental insufficiency" ~3 OR "placental insufficiencies" ~3 OR "placentae insufficiency" ~3 OR "placentae insufficiencies" ~3 OR "amnion infection" ~3 OR "amnion infections" ~3 OR "amniotic infection" ~3 OR "amniotic infections" ~3 OR "amnion embolism" ~3 OR "amnion embolisms" ~3 OR "amniotic embolism" ~3 OR "amniotic embolisms" ~3 OR "gestational pemphigoid" ~3 OR "gestationis pemphigoid" ~3

OR

"uterine rupture" ~3 OR "uterine ruptured" ~3 OR "multiple gestation" ~3 OR "multiple gestations" ~3 OR "twin gestation" ~3 OR "twin gestations" ~3 OR "triplet gestation" ~3 OR "triplet gestations" ~3

OR

"premature rupture of membranes" OR "rupture membranes prematurely" OR "premature birth" ~3 OR "premature births" ~3 OR "pre-mature birth" ~3 OR "pre-mature births" ~3 OR "preterm birth" ~3 OR "preterm births" ~3 OR "pre-term birth" ~3 OR "pre-term births" ~3 OR "premature delivery" ~3 OR "premature deliveries" ~3 OR "pre-mature delivery" ~3 OR "pre-mature deliveries" ~3 OR "preterm delivery" ~3 OR "preterm deliveries" ~3 OR "pre-term delivery" ~3 OR "pre-term deliveries" ~3

AND

Africa OR African OR Asia OR Asian OR Caribbean OR "West Indies" OR "South America" OR "South American" OR "Latin America" OR "Latin American" OR "Central America" OR "Central American"

OR

Afghanistan OR Albania OR Algeria OR Angola OR Antigua OR Barbuda OR Argentina OR Armenia OR Armenian OR Aruba OR Azerbaijan OR Bahrain OR Bangladesh OR Barbados OR Benin OR Byelarus OR Byelorussian OR Belarus OR Belorussian OR Belorussia OR Belize OR Bhutan OR Bolivia OR Bosnia OR Herzegovina OR Hercegovina OR Botswana OR Brasil OR Brazil OR "Brazzavilee" OR Bulgaria OR Burkina Faso OR Burkina Fasso OR Upper Volta OR Burundi OR Urundi OR Cambodia OR Khmer Republic OR Kampuchea OR Cameroon OR Cameroons OR Cameron OR Camerons OR "Canary Islands" OR Cape Verde OR Central African Republic OR Chad OR Chile OR China OR Colombia OR Comoros OR Comoro Islands OR Comores OR Mayotte OR Congo OR Zaire OR Costa Rica OR Cote d'Ivoire OR Ivory Coast OR Croatia OR Cuba OR Cyprus OR Czechoslovakia OR Czech Republic OR Slovakia OR "Slovak Republic" OR Djibouti OR "French Somaliland" OR Dominica OR "Dominican Republic" OR "East Timor" OR "East Timur" OR "Timor Leste" OR Ecuador OR Egypt OR "United Arab Republic" OR El Salvador OR Eritrea OR Estonia OR Ethiopia OR Fiji OR Gabon OR "Gabonese Republic" OR Gambia OR Gaza OR "Georgia Republic" OR "Georgian Republic" OR Ghana OR Gold Coast OR Greece OR Grenada OR Guatemala OR Guinea OR Guam OR Guiana OR Guyana OR Haiti OR Honduras OR Hungary OR India OR Maldives OR Indonesia OR Iran OR Iraq OR Isle of Man OR Jamaica OR Jamahiriya OR Jamahiryia OR Jordan OR Kazakhstan OR Kazakh OR Kenya OR Kiribati OR Korea OR Kosovo OR Kyrgyzstan OR Kirghizia OR Kyrgyz Republic OR Kirghiz OR Kirgizstan OR Lao PDR OR Laos OR Latvia OR Lebanon OR Lesotho OR Basutoland OR Liberia OR Libia OR Libya OR Lithuania OR Macedonia OR Madagascar OR Maghreb OR Maghrib OR "Malagasy Republic" OR Malaysia OR Malaya OR Malay OR Mayote OR Mocambique OR Principe OR Reunion OR Sabah OR Sarawak OR Malawi OR Nyasaland OR Mali OR Malta OR "Marshall Islands" OR Mauritania OR Mauritius OR Agalega Islands OR Mexico OR Micronesia OR "Middle East" OR Moldova OR Moldovia OR Moldovian OR Mongolia OR Montenegro OR Morocco OR Ifni OR Mozambique OR Myanmar OR Myanma OR Burma OR Namibia OR Nepal OR "Netherlands Antilles" OR New Caledonia OR Nicaragua OR Niger OR Nigeria OR "Northern Mariana Islands" OR Oman OR Muscat OR Pakistan OR Palau OR Palestine OR Panama OR Paraguay OR Peru OR Philippines OR Philipines OR Phillipines OR Phillippines OR Poland OR Portugal OR "Puerto Rico" OR Romania OR Rumania OR Roumania OR Russia OR Russian OR Rwanda OR Ruanda OR "Saint Kitts" OR "St Kitts" OR Nevis OR "Saint Lucia" OR "St Lucia" OR "Saint Vincent" OR "St Vincent" OR Grenadines OR Samoa OR "Samoan Islands" OR "Navigator Island" OR "Navigator Islands" OR "St Helena" OR "Saint Helena" OR "Sao Tome" OR "Saudi Arabia" OR Senegal OR Serbia OR Montenegro OR Seychelles OR Sierra Leone OR Slovenia OR "Sri Lanka" OR Ceylon OR "Solomon Islands" OR Somalia OR "South Africa" OR Sudan OR

Suriname OR Surinam OR Swaziland OR Syria OR Tajikistan OR Tadzhikistan OR Tadjikistan OR Tadzhik OR Tanzania OR Thailand OR Togo OR Togolese Republic OR Tonga OR Trinidad OR Tobago OR Tunisia OR Turkey OR Turkmenistan OR Turkmen OR Uganda OR Ukraine OR Uruguay OR USSR OR "Soviet Union" OR "Union of Soviet Socialist Republics" OR Uzbekistan OR Uzbek OR Vanuatu OR "New Hebrides" OR Venezuela OR Vietnam OR Viet Nam OR West Bank OR "Western Sahara" OR "Western Saharan" OR Yemen OR Yugoslavia OR Zambia OR Zimbabwe OR Rhodesia

OR

“developing country” OR “developing countries” OR “developing nation” OR “developing nations” OR “developing population” OR “developing populations” OR “developing world” OR “developing worlds” OR “less developed country” OR “less developed countries” OR “less developed nation” OR “less developed nations” OR “less developed population” OR “less developed populations” OR “less developed world” OR “less developed worlds” OR “lesser developed country” OR “lesser developed countries” OR “lesser developed nation” OR “lesser developed nations” OR “lesser developed population” OR “lesser developed populations” OR “lesser developed world” OR “lesser developed worlds” OR “under developed country” OR “under developed countries” OR “under developed nation” OR “under developed nations” OR “under developed population” OR “under developed populations” OR “under developed world” OR “under developed worlds” OR “underdeveloped country” OR “underdeveloped countries” OR “underdeveloped nation” OR “underdeveloped nations” OR “underdeveloped population” OR “underdeveloped populations” OR “underdeveloped world” OR “underdeveloped worlds” OR “middle income country” OR “middle income countries” OR “middle income nation” OR “middle income nations” OR “middle income population” OR “middle income populations” OR “middle income world” OR “middle income worlds” OR “low income country” OR “low income countries” OR “low income nation” OR “low income nations” OR “low income population” OR “low income populations” OR “low income world” OR “low income worlds” OR “lower income country” OR “lower income countries” OR “lower income nation” OR “lower income nations” OR “lower income population” OR “lower income populations” OR “lower income world” OR “lower income worlds” OR “underserved country” OR “underserved countries” OR “underserved nation” OR “underserved nations” OR “underserved population” OR “underserved populations” OR “underserved world” OR “underserved worlds” OR “under-served country” OR “under-served countries” OR “under-served nation” OR “under-served nations” OR “under-served population” OR “under-served populations” OR “under-served world” OR “under-served worlds” OR “deprived country” OR “deprived countries” OR “deprived nation” OR “deprived nations” OR “deprived population” OR “deprived populations” OR “deprived world” OR “deprived worlds” OR “poor country” OR “poor countries” OR “poor nation” OR “poor nations” OR “poor population” OR “poor populations” OR “poor world” OR “poor worlds” OR “poorer country” OR “poorer countries” OR “poorer nation” OR “poorer nations” OR “poorer population” OR “poorer populations” OR “poorer world” OR “poorer worlds”

OR

“developing economy” OR “developing economies” OR “less developed economy” OR “less developed economies” OR “lesser developed economy” OR “lesser developed economies” OR “under developed economy” OR “under developed economies” OR “underdeveloped economy” OR “underdeveloped economics” OR “middle income economy” OR “middle income economies” OR “low income economy” OR “low income economies” OR “lower income economy” OR “lower income economies”

OR

"low income country" OR "low income countries" OR "middle income countries" OR "middle income countires"

OR

“low GDP” OR “low GNP” OR “low gross domestic” OR “low gross nation” OR “lower GDP” OR “lower GNP” OR “lower gross domestic” OR “lower gross national”

OR

lmic OR lmics OR "third world" OR "lami country" OR "lami countries"

OR

"transitional country" OR "transitional countries"

Web of Science

31 29,268
#30 AND #29
Indexes=SCI-EXPANDED, CPCI-S Timespan=All years
# 30 17,740,570
PY=(2009-2017)
Indexes=SCI-EXPANDED, CPCI-S Timespan=All years
# 29 54,027
#28 OR #18
Indexes=SCI-EXPANDED, CPCI-S Timespan=All years
# 28 20,236
#27 AND #10
Indexes=SCI-EXPANDED, CPCI-S Timespan=All years
# 27 2,411,882
#26 OR #25 OR #24 OR #23 OR #22 OR #21 OR #20 OR #19
Indexes=SCI-EXPANDED, CPCI-S Timespan=All years
# 26 186
TS=("transitional country" OR "transitional countries")
Indexes=SCI-EXPANDED, CPCI-S Timespan=All years
# 25 4,603
TS=(lmic OR lmics OR "third world" OR "lami country" OR "lami countries")
Indexes=SCI-EXPANDED, CPCI-S Timespan=All years
# 24 222
TS=(“low GDP” OR “low GNP” OR “low gross domestic” OR “low gross national” OR “lower GDP” OR “lower GNP” OR “lower gross domestic” OR “lower gross national”)
Indexes=SCI-EXPANDED, CPCI-S Timespan=All years
# 23 1,043
TS=("developing economy" OR "developing economies" OR "less developed economy" OR "less developed economies" OR "lesser developed economy" OR "lesser developed economies" OR "under developed economy" OR "under developed economies" OR "underdeveloped economy" OR "underdeveloped economics" OR "middle income

economy" OR "middle income economies" OR "low income economy" OR "low income economies" OR "lower income economy" OR "lower income economies")
Indexes=SCI-EXPANDED, CPCI-S Timespan=All years
# 22 124,549
TS=((developing or "less developed" or "lesser developed" or "under developed" or underdeveloped or "middle income" or "low income" or "lower income" or underserved or "under served" or deprived or poor*) NEAR/1 (countr* or nation$ or population$ or world$))
Indexes=SCI-EXPANDED, CPCI-S Timespan=All years
# 21 2,061,205
TS=(Afghanistan or Albania or Algeria or Angola or Antigua or Barbuda or Argentina or Armenia or Armenian or Aruba or Azerbaijan or Bahrain or Bangladesh or Barbados or Benin or Byelarus or Byelorussian or Belarus or Belorussian or Belorussia or Belize or Bhutan or Bolivia or Bosnia or Herzegovina or Hercegovina or Botswana or Brasil or Brazil or "Brazzavilee" or Bulgaria or Burkina Faso or Burkina Fasso or "Upper Volta" or Burundi or Urundi or Cambodia or "Khmer Republic" or Kampuchea or Cameroon or Cameroons or Cameron or Camerons or "Canary Islands" or "Cape Verde" or "Central African Republic" or Chad or Chile or China or Colombia or Comoros or "Comoro Islands" or Comores or Mayotte or Congo or Zaire or "Costa Rica" or "Cote d'Ivoire" or "Ivory Coast" or Croatia or Cuba or Cyprus or Czechoslovakia or "Czech Republic" or Slovakia or "Slovak Republic" or Djibouti or "French Somaliland" or Dominica or "Dominican Republic" or "East Timor" or "East Timur" or "Timor Leste" or Ecuador or Egypt or "United Arab Republic" or "El Salvador" or Eritrea or Estonia or Ethiopia or Fiji or Gabon or "Gabonese Republic" or Gambia or Gaza or "Georgia Republic" or "Georgian Republic" or Ghana or "Gold Coast" or Greece or Grenada or Guatemala or Guinea or Guam or Guiana or Guyana or Haiti or Honduras or Hungary or India or Maldives or Indonesia or Iran or Iraq or Isle of Man or Jamaica or Jamahiriya$ or Jamahiryia$ or Jordan or Kazakhstan or Kazakh or Kenya or Kiribati or Korea or Kosovo or Kyrgyzstan or Kirghizia or "Kyrgyz Republic" or Kirghiz or Kirgizstan or "Lao PDR" or Laos or Latvia or Lebanon or Lesotho or Basutoland or Liberia or Libia or Libya or Lithuania or Macedonia or Madagascar or Maghreb or Maghrib or "Malagasy Republic" or Malaysia or Malaya or Malay or Mayote or Mocambique or Principe or Reunion or Sabah or Sarawak or Malawi or Nyasaland or Mali or Malta or "Marshall Islands" or Mauritania or Mauritius or "Agalega Islands" or Mexico or Micronesia or "Middle East" or Moldova or Moldovia or Moldovian or Mongolia or Montenegro or Morocco or Ifni or Mozambique or Myanmar or Myanma or Burma or Namibia or Nepal or "Netherlands Antilles" or "New Caledonia" or Nicaragua or Niger or Nigeria or "Northern Mariana Islands" or Oman or Muscat or Pakistan or Palau or Palestine or Panama or Paraguay or Peru or Philippines or Philipines or Phillipines or Phillippines or Poland or Portugal or "Puerto Rico" or Romania or Rumania or Roumania or Russia or Russian or Rwanda or Ruanda or "Saint Kitts" or "St Kitts" or Nevis or "Saint Lucia" or "St Lucia" or "Saint Vincent" or "St Vincent" or Grenadines or Samoa or "Samoan Islands" or "Navigator Island" or "Navigator Islands" or "St Helena" or "Saint Helena" or "Sao Tome" or "Saudi Arabia" or Senegal or Serbia or Montenegro or Seychelles or "Sierra Leone" or Slovenia or "Sri Lanka" or Ceylon or "Solomon Islands" or Somalia or "South Africa" or Sudan or Suriname or Surinam or Swaziland or Syria or Tajikistan or Tadzhikistan or Tadjikistan or Tadzhik or Tanzania or Thailand or Togo or "Togolese Republic" or Tonga or Trinidad or Tobago or Tunisia or Turkey or Turkmenistan or Turkmen or Uganda or Ukraine or Uruguay or USSR or "Soviet Union" or "Union of Soviet Socialist Republics" or Uzbekistan or Uzbek or Vanuatu or "New Hebrides" or Venezuela or Vietnam or "Viet Nam" or "West Bank" or "Western Sahara" or "Western Saharan" or Yemen or Yugoslavia or Zambia or Zimbabwe or Rhodesia)
Indexes=SCI-EXPANDED, CPCI-S Timespan=All years
# 20 543,014

TS=(Africa$ or Asia$ or Caribbean or "West Indies" or "South America" OR "South American" or "Latin America" or "Latin American" or "Central America" or "Central American")
Indexes=SCI-EXPANDED, CPCI-S Timespan=All years
# 19 61,752
TS="Developing Countries"
Indexes=SCI-EXPANDED, CPCI-S Timespan=All years
# 18 48,259
#17 AND #10
Indexes=SCI-EXPANDED, CPCI-S Timespan=All years
# 17 2,896,816
#16 OR #15 OR #14 OR #13 OR #12 OR #11
Indexes=SCI-EXPANDED, CPCI-S Timespan=All years
# 16 631
TS=(confidential NEAR/1 (inquir* or enquir*))
Indexes=SCI-EXPANDED, CPCI-S Timespan=All years
# 15 88
TS=("cause-specific mortality" NEAR/1 fraction*) OR TS=CSMF
Indexes=SCI-EXPANDED, CPCI-S Timespan=All years
# 14 362,859
TS=((dead or death or deaths or decease* or demise* or die or died or dies or dying or fatal or fatality or fatalities or homicid* or mortal or mortality or mortalities or murder* or nonsurvival* or non-survival* or suicid*) NEAR/3 (audit* or cause or causes or data or determinant* or estimat* or frequenc* or number or numbers or rate or rates or reason or reasons or report* or statistic* or trend*))
Indexes=SCI-EXPANDED, CPCI-S Timespan=All years
# 13 3,314
TS=("vital statistic" or "vital statistics" or "vital registration" or "vital registrations")
Indexes=SCI-EXPANDED, CPCI-S Timespan=All years
# 12 2,604,034
TS=epidemiolog* OR TS=(survey* or questionnaire*) OR TS=(incidenc* or prevalen*) OR TS=((clinical or disease* or factual) NEAR/1 database*) OR TS=(register or registers or registry or registries) OR TS=surveillance*
Indexes=SCI-EXPANDED, CPCI-S Timespan=All years
# 11 62,842
TS=(maternal* NEAR/3 mortalit*) OR TS=autops* OR TS=(death$ NEAR/2 (certificat* or certify or certified or certifies)) OR TS=(death$ NEAR/2 review$)
Indexes=SCI-EXPANDED, CPCI-S Timespan=All years
# 10 103,075
#9 AND #1
Indexes=SCI-EXPANDED, CPCI-S Timespan=All years
# 9 1,037,330
#8 OR #7 OR #6 OR #5 OR #4 OR #3 OR #2
Indexes=SCI-EXPANDED, CPCI-S Timespan=All years
# 8 131,077
TS=(obstetric* or miscarr* or abort*) OR TS=(postabort* or post-abort*) OR TS=((dilatat* or dilat* or vacuum* or suction*) NEAR/2 curettage*) OR TS=((uterus or uteri or uterin* or vacuum*) NEAR/2 aspirat*) OR TS=((dilatat* or dilat*) NEAR/2 evacuat*)
Indexes=SCI-EXPANDED, CPCI-S Timespan=All years
# 7 19,066
TS=(placenta* NEAR/3 (disease* or disorder* or syndrome*)) OR TS=(placenta* NEAR/3 (abrupti* or accreta* or previa* or retained)) OR TS=(placenta* NEAR/3 insufficien*) OR

TS=(placenta* NEAR/3 (prematur* NEAR/2 separat*)) OR TS=(amnionit?s or chorioamnionit?s or funisit?s) OR TS=(amnio* NEAR/3 infect*) OR TS=(amnio* NEAR/3 emboli*) OR TS=(gestation* NEAR/1 pemphigoid*) OR TS=(hydramnios or oligohydramnios or polyhydramnios) OR TS="vasa previa"
Indexes=SCI-EXPANDED, CPCI-S Timespan=All years
# 6 30,073
TS=(gestation* NEAR/2 diabet*) OR TS=(gestation* NEAR/2 (edema* or hypertensi* or oedema* or proteinuria*)) OR TS=(eclamp* or pre-eclamp* or HELLP) OR TS=((EPH or "Edema-Proteinuria-Hypertension" or "Hypertension-Edema-Proteinuria" or "Proteinuria-Edema-Hypertension") NEAR/2 (complex* or gestos?s or tox$emi*))
Indexes=SCI-EXPANDED, CPCI-S Timespan=All years
# 5 533,774
TS=(maternal* or maternit* or mother*) OR TS=(antenatal* or ante-natal* or antepartum* or ante-partum* or intrapartum* or intra-partum* or perinatal* or peri-natal* or peripartum* or peri-partum* or prenatal* or pre-natal* or postnatal* or post-natal* or postpartum* or post-partum* or puerperal* or puerperium) OR TS=("hyperemesis gravidarum" or "morning sickness")
Indexes=SCI-EXPANDED, CPCI-S Timespan=All years
# 4 89,426
TS=((premature* or pre-mature* or preterm* or pre-term*) NEAR/3 (birth* or deliver* or labor* or labour*)) OR TS=((premature* or pre-mature* or preterm* or pre-term*) NEAR/3 (membran* NEAR/1 ruptur*)) OR TS=(caesarean* or cesarean* or "c-section" or "c-sections" or post-caesarean* or post-cesarean*) OR TS=(VBAC or VBACs) OR TS=(contract* NEAR/3 (hypertonic or incoordinate* or prolonged)) OR TS=episiotom* OR TS=(perineal NEAR/1 lacerat*) OR TS=((hydatidiform or nonhydatidiform or non-hydatidiform) NEAR/1 mole$) OR TS=((multiple* or twin or twins or triplet$) NEAR/1 gestation*)
Indexes=SCI-EXPANDED, CPCI-S Timespan=All years
# 3 34,627
TS=((uterin* or uterus or uteri*) NEAR/1 inert*) OR TS=((labor* or labour*) NEAR/3 induc*) OR TS=((labor* or labour*) NEAR/3 obstruct*) OR TS=((labor* or labour*) NEAR/3 (delayed or failed or false or prolong*)) OR TS=((labor* or labour*) NEAR/3 precipitate*) OR TS=((labor* or labour*) NEAR/3 (birth* or deliver*)) OR TS=((abdominal* or vaginal*) NEAR/3 (birth* or deliver*)) OR TS=(breech NEAR/3 (birth* or deliver* or extract*)) OR TS=((forceps or instrumental or vacuum) NEAR/3 (birth* or deliver* or extract*))
Indexes=SCI-EXPANDED, CPCI-S Timespan=All years
# 2 567,343
TS=(pregnan* or parturition* or childbirth* or child birth* or gravidity or primigrav* or primi-grav* or multigrav* or multi-grav* or nulligrav* or nulli-grav* or parity or primipar* or primi-par* or multipar* or multi-par* or nullipar* or nulli-par*) OR TS=dystocia*
Indexes=SCI-EXPANDED, CPCI-S Timespan=All years
# 1 2,029,943
TS=(mortal or mortality or mortalities) OR TS=(dead or death or deaths or decease* or demise* or die or died or dies or dying) OR TS=(fatal or fatality or fatalities) OR TS=(non-survival$ or nonsurvival$) OR TS="not survive" OR TS=(suicid* or homicid* or kill or killed or kills or killing or murder*)
Indexes=SCI-EXPANDED, CPCI-S Timespan=All years

Global Index Medicus
(tw:(death or deaths or fatal or fatality or fatalities or deceased or demise or die or died or dies or dying)) AND (tw:(maternal or pregnancy or pregnant or prenatal or perinatal or peripartum or antenatal or antepartum or intrapartum or postnatal or postpartum or

puerperal or puerperium)) AND (tw:(mortality or epidemiology or epidemiological or frequency or frequencies or incidence or prevalence or surveillance))

(tw:(mort or deces or mortels or mortels or mortels or decedes or morts or morts or morts or meurt or mourants)) AND (tw:(maternel or grossesse or enceinte or prenatal or perinatal or perinatal or prenatal or antepartum or intrapartum or postnatal or post-partum or puerperale or puerperalite)) AND (tw:(mortalite or epidemiologie or epidemiologique or frequence or incidence or prevalence or surveillance))

(tw:(Muerte or muerte or muerte or fatalidad or muerte or muerte or fallecimiento or muere or muere or muere or muere)) AND (tw:(maternal or embarazo or embarazada or prenatal or perinatal or periparto or prenatal or anteparto or intraparto or postnatal or postparto or puerperal or puerperio)) AND (tw:(mortalidad or epidemiología or epidemiología or frecuencia or frecuencia or incidencia or prevalencia or vigilancia))

Figure S1 PRISMA for Search 1a

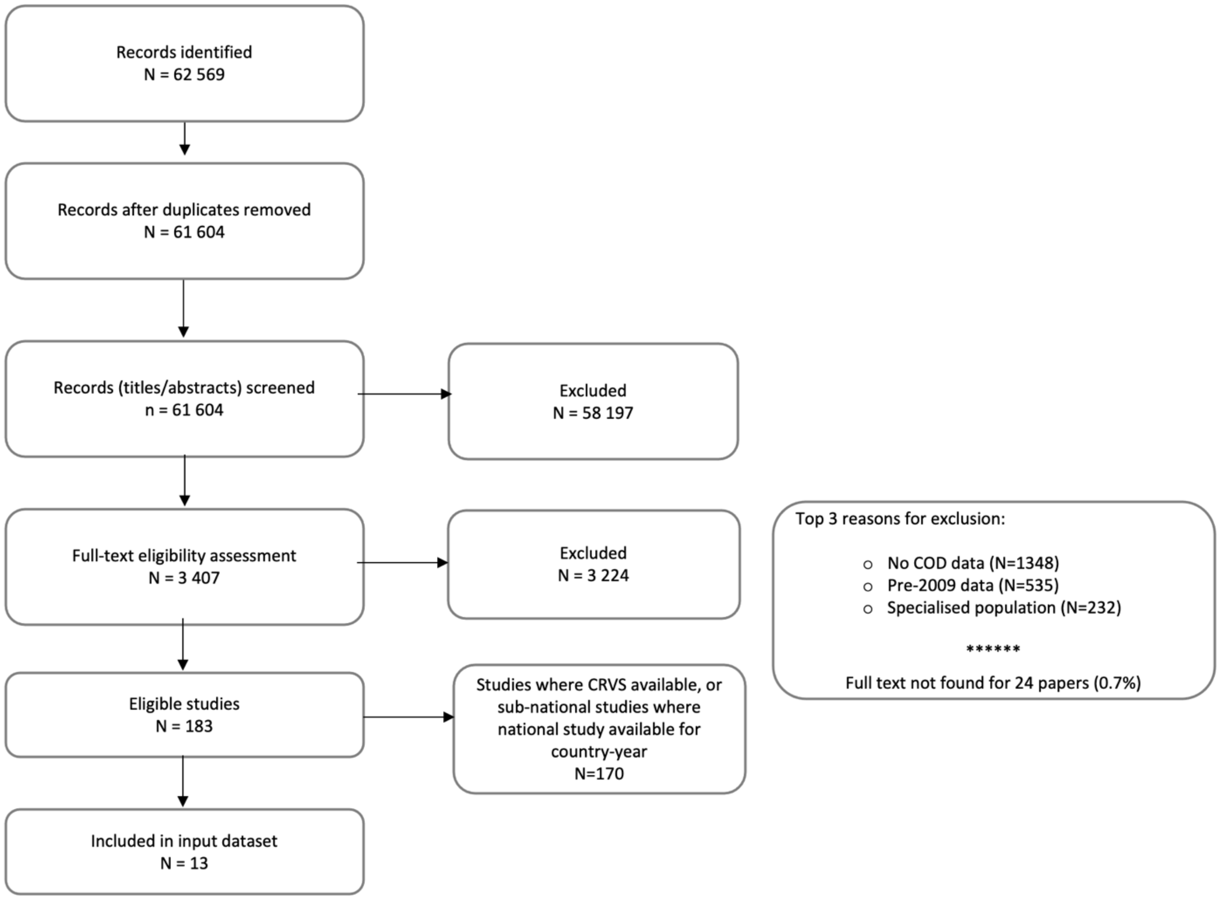

Search 1B: Russian language database

eLIBRARY.RU

The following search was conducted in January 2018.

Название подборки
На 23.01.18
материнская смертность
смертность беременность
материнская летальность
материнская смертность_итог
смертность аборт
смертность роды
смертность родоразрешение
смертность кесарево сечение
смертность перинатальный период
смертность перинатальный период материнская
смертность плацента
акушерская смертность
смертность матка беременность
летальность материнская
суицид беременность

причины смерти беременность

на 25.01.18
материнская смертность
смертность беременность
материнская летальность
материнская смертность_итог
смертность аборт
смертность роды
смертность родоразрешение
смертность кесарево сечение
смертность перинатальный период
смертность перинатальный период материнская
смертность плацента
акушерская смертность
смертность матка беременность
летальность материнская
суицид беременность
причины смерти беременность
летальность материнская
летальность беременность
материнская популяция смертность

Figure S2 PRISMA for Search 1b

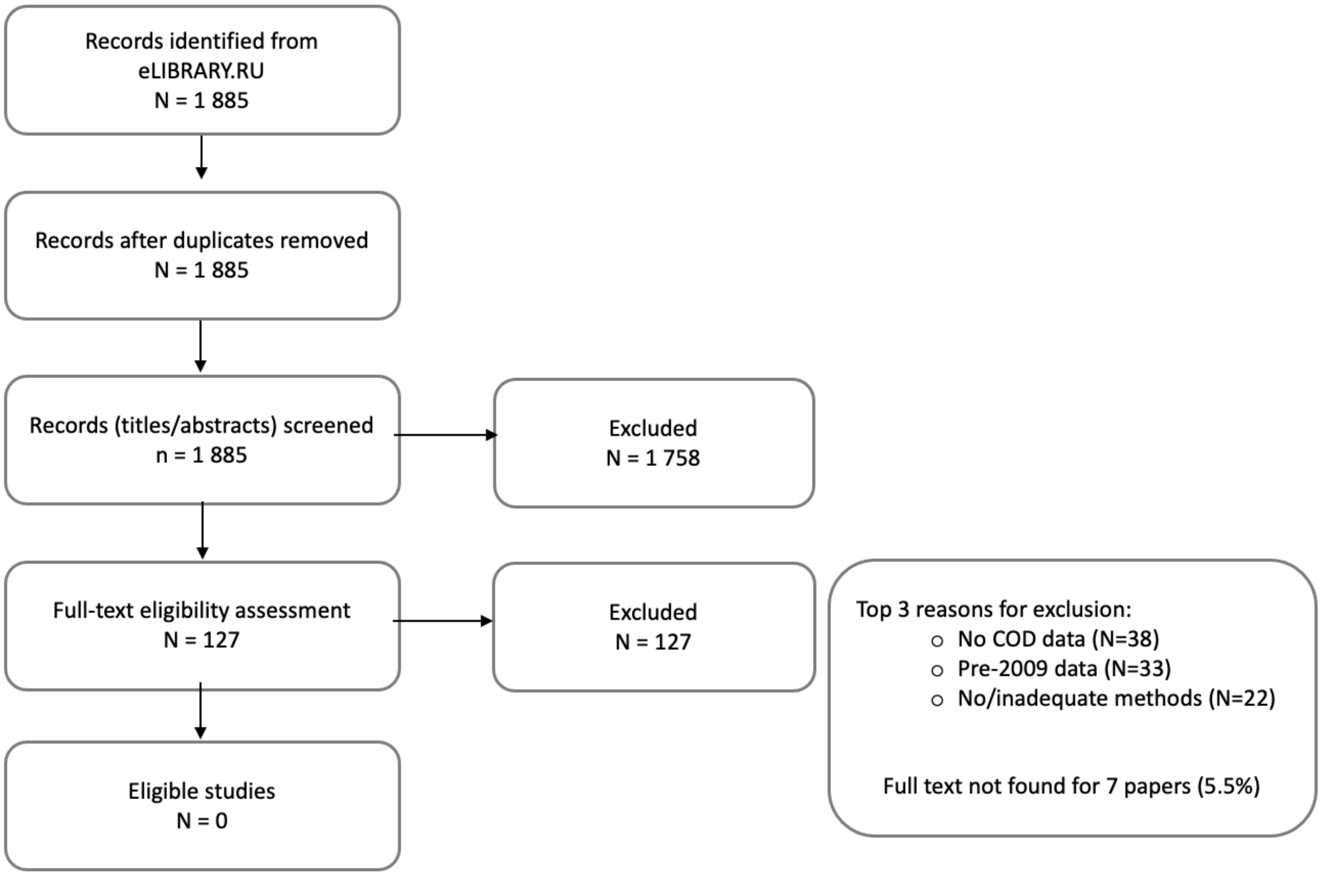

Search 1C: Chinese language databases

Wanfang

主题:(孕产妇死亡)+(摘要:(死+自杀+流行病)*主题:(孕产妇)*摘要:(孕期+怀孕+妊娠+妊娠期+怀孕期+怀孕妇女+孕妇+孕期妇女+妊娠妇女+产妇+妊娠妇+经产妇+初产妇+产前+产时+产后+妊娠晚期+分娩)*摘要:(难产+孕期风险+妊娠期合并症+妊娠并发症+妊娠期并发症+妊娠合并症+怀孕并发症+阻塞性分娩+滞产+急产+剖宫产+臀位分娩+器械分娩+早产+高张性挛缩+小产+流产+引产+死胎+妊娠期糖尿病+妊娠高血压+妊娠子痫前期+妊娠先兆子痫+妊娠HELLP综合征+妊娠高血压综合征+胎盘早剥+产褥期感染+感染+产科出血+?疾病))^题名:(动物+胎儿+新生儿+婴儿+围产儿+儿童)

CNKI

AB=('死'+'自杀'+'流行病') and SU='孕产妇' and AB= ('孕期'+'怀孕'+'妊娠'+'妊娠期'+'怀孕期'+'怀孕妇女'+'孕妇'+'孕期妇女'+'妊娠妇女'+'产妇'+'妊娠妇'+'经产妇'+'初产妇'+'产前'+'产时'+'产后'+'妊娠晚期'+'分娩') and AB= ('难产'+'孕期风险'+'妊娠期合并症'+'妊娠并发症'+'妊娠期并发症'+'妊娠合并症'+'怀孕并发症'+'阻塞性分娩'+'滞产'+'急产'+'剖宫产'+'臀位分娩'+'器械分娩'+'早产'+'高张性挛缩'+'小产'+'流产'+'引产'+'死胎'+'妊娠期糖尿病'+'妊娠高血压'+'妊娠子痫前期'+'妊娠先兆子痫'+'妊娠HELLP综合征'+'妊娠高血压综合征'+'胎盘早剥'+'产褥期感染'+'感染'+'产科出血'+'?疾病') or AB=孕产妇死亡 not TI=('动物'+'胎儿'+'新生儿'+'婴儿'+'围产儿'+'儿童')

Figure S3 PRISMA for Search 1C

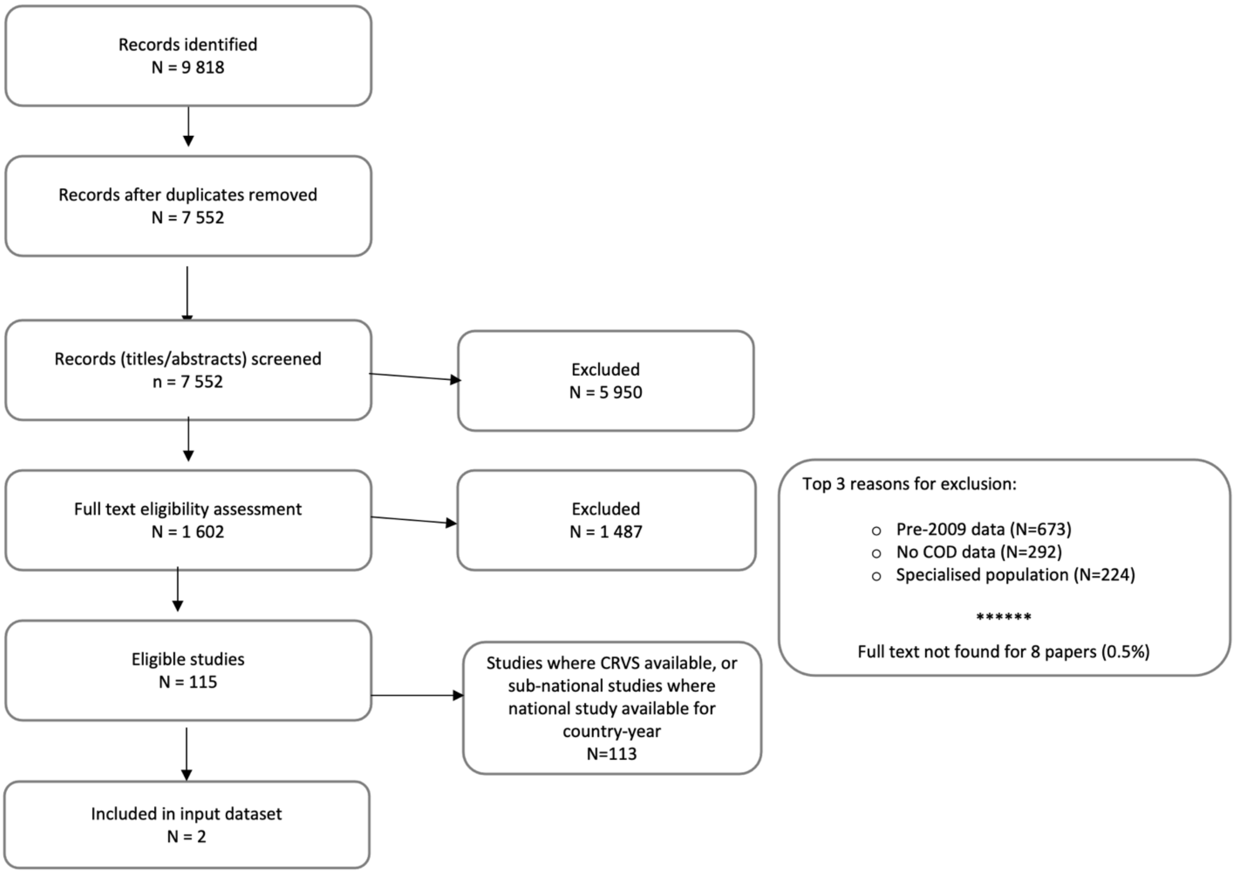

Search 2: Data from 2017-2020

Only Search 1A on MEDLINE, Embase, and Global Index Medicus was repeated for the second update for data from 2017-2020 due to low yield of Search 1B and 1C. The search terms were also simplified. The following searches were conducted in August 2023.

MEDLINE

\# Searches Results
Ovid MEDLINE(R) ALL 1946 to August 02, 2023
1 Maternal Death/ or Fatal Outcome/ or Suicide/ or Homicide/ 118711
2 (mortal or mortality or mortalities or dead or death or deaths or decease* or demise* or died or dies or dying or fatal or fatality or fatalities or non-survival* or nonsurvival* or not-surviv* or suicid* or homicid* or kill or killed or kills or killing or murder*).tw,kf. 2381219
3 1 or 2 [DEATH] 2427368
4 exp Pregnancy/ or exp Pregnancy Complications/ or Pregnant Women/ or exp Pregnancy Trimesters/ or Prenatal Care/ or Postnatal Care/ or Peripartum Period/ or Postpartum Period/ or Maternal Health/ or Abortion, Criminal/ or Vacuum Curettage/ or exp Abortifacient Agents/ 1105593
5 (pregnant or pregnanc* or (parturition* or childbirth* or child-birth*) or ((labor or labour) adj3 (birth* or deliver*)) or (maternal* or maternit* or mother*) or miscarr* or abortion* abortive* or (postabort* or post-abort*)).tw,kf. 941461
6 or/4-5 [Pregnancy|Maternal|Obstetrics|Abortion] 1451604
7 Maternal Mortality/ or Cause of Death/ or Mortality/ or Hospital Mortality/ or Autopsy/ or Death Certificates/ or Incidence/ or Prevalence/ or exp Databases, Factual/ or Survival Rate/ or Vital Statistics/ 1113537.00

8 sn.fs. [STATISTICS & NUMERICAL DATA] 1039339.00
9 mo.fs. [MORTALITY STATISTICS] 628545
10 (autops* or (death? adj2 (certificat* or certify or certified or certifies)) or (death? adj2 review?) or (death? adj2 classification or classify) or incidenc* or prevalen* or ((clinical or disease* or factual) adj database*) or (register or registers or registry or registries) or surveillance* or (vital statistic* or vital registration?) or ((dead or death or deaths or decease* or demise* or died or dies or dying or fatal or fatality or fatalities or homicid* or mortal or mortality or mortalities or murder* or nonsurvival* or non-survival* or suicid*) adj3 (burden or data or estimat* or frequenc* or figure* or ratio or ratios or rate or rates or report* or statistic* or reporting or underreporting or index*)) or Reproductive-Age-Mortality-Study or cause-specific-mortality-fraction* or cause-specifc* or "cause specific" or (confidential adj (inquir* or enquir*))).tw,kf. 2585637
11 7 or 8 or 9 or 10 [MORTALITY STATISTICS] 4072963
12 3 and 6 and 11 71112
13 limit 12 to dt=20171216-20230807 20774
14 exp Child/ not (exp Child/ and exp Adult/)
15 exp Infant/ not (exp Infant/ and exp Adult/)
16 exp fetus/ not (exp fetus/ and exp adult/)
17 exp newborn/ not (exp newborn/and exp adult/)
18 exp animals/ not (exp animals/ and humans/)
19 male/ not (male/ and female/)
20 (cow or cows or bovine* or calve or calves or bitch* or pig or pigs or piglet*).ti.
21 14 or 15 or 16 or 17 or 18 or 19 or 20
22 13 not 21 [Child & Animal Removed]
23 ("case report" or "case series" or "case presentation" or "single-case study" or "randomi*ed control* trial" or "RCT" or "placebo" or "drug therapy").ti.
24 22 not 23 [study design removed]
25 (("a tertiary" adj2 (hospital or facility or centre or center or "care center" or "care centre")) or ("a referral" adj2 (hospital or facility or center or centre or "care center" or "care centre"))).ti.
26 24 not 25 [single hospital studies removed]
27 (australia* or mauritius* or antigua* or barbuda* or argentin* or aruba* or brazil* or chile* or "Costa Rica" or colombia* or cuba* or dominica* or ecuador* or grenada* or guatemala* or mexico* or montserrat* or nicaragua* or paraguay* or peru* or "Saint Lucia" or "Saint Vincent and Grenadines" or "United States of America" or uruguay* or cyprus* or Israel* or Japan* or Malaysia* or mongolia* or oman* or "Republic of Korea" or Singapore* or Armenia* or Andorra* or Austria* or "Bosnia and Herzegovina" or Bulgaria* or "Czech Republic" or Czechia* or denmark* or Estonia* or finland* or georgia* or germany* or Greece* or Iceland* or Kazakhstan* or Latvia* or Lithuania* or Luxembourg* or Macedonia* or malta* or netherlands* or Poland* or serbia* or "San Marino" or Slovenia* or Spain* or Switzerland* or "United Kingdom" or England* or "England and Wales" or Wales* or "Northern Ireland" or scotland*).tw,kf.
28 26 not 27 [CRVS COUNTRIES Removed] 10252

EMBASE

#	Searches	Results
	Embase Classic+Embase 1947 to 2023 August 02	
1	Maternal Death/ or Fatality/ or Suicide/ or Homicide/	191'524
2	(mortal or mortality or mortalities or dead or death or deaths or decease* or demise* or died or dies or dying or fatal or fatality or fatalities or non-survival* or nonsurvival* or not-surviv* or suicid* or homicid* or kill or killed or kills or killing or murder*).tw,kf.	3'526'195
3	1 or 2 [DEATH]	3'578'591

4 exp Pregnancy/ or exp pregnancy disorder/ or exp named groups by pregnancy/ or exp maternal care/ or prenatal care/ or perinatal care/ or postnatal care/ or puerperium/ or perinatal period/ or prenatal period/ or exp obstetric delivery/ or Postpartum Period/ or Maternal Health/ or Abortion, Criminal/ or Vacuum Curettage/ or exp abortive agent/ 1'643'071
5 (pregnant or pregnanc* or (parturition* or childbirth* or child-birth*) or ((labor or labour) adj3 (birth* or deliver*)) or (maternal* or maternit* or mother*) or miscarr* or abortion* abortive* or (postabort* or post-abort*)).tw,kf. 1'278'681
6 or/4-5 [Pregnancy|Maternal|Obstetrics|Abortion] 2'055'616
7 Maternal Mortality/ or "cause of death"/ or Mortality/ or Hospital Mortality/ or Autopsy/ or Death Certificate/ or exp Data Collection/ or exp Incidence/ or exp Prevalence/ or Factual Database/ or sentinel surveillance/ or exp mortality rate/ or Vital Statistics/ 5'231'051
8 (autops* or (death? adj2 (certificat* or certify or certified or certifies)) or (death? adj2 review?) or (death? adj2 classification or classify) or incidenc* or prevalen* or ((clinical or disease* or factual) adj database*) or (register or registers or registry or registries) or surveillance* or (vital statistic* or vital registration?) or ((dead or death or deaths or decease* or demise* or died or dies or dying or fatal or fatality or fatalities or homicid* or mortal or mortality or mortalities or murder* or nonsurvival* or non-survival* or suicid*) adj3 (burden or data or estimat* or frequenc* or figure* or ratio or ratios or rate or rates or report* or statistic* or reporting or underreporting or index*)) or Reproductive-Age-Mortality-Study or cause-specific-mortality-fraction* or cause-specifc or "cause specific" or (confidential adj (inquir* or enquir*))).tw,kf. 3'802'986
9 or/7-8 [MORTALITY STATISTICS/EPIDEMIOLGY] 7'162'750
10 3 and 6 and 9 128'764
11 limit 10 to dt=20171216-20230807
12 exp animal experimentation/ or exp animal model/ or exp animal experiment/ or nonhuman/ or exp vertebrate/
13 exp human/ or exp human experimentation/ or exp human experiment/
14 12 not 13
15 (cow or cows or bovine* or calve or calves or bitch* or pig or pigs or piglet*).ti.
16 14 or 15
17 11 not 16 [ANIMALS REMOVED]
18 male/ not (male/ and female/)
19 17 not 18 [MALE REMOVED]
20 exp child/ not (exp child/ and exp adult/)
21 exp infant/ not (exp infant/ and exp adult/)
22 exp fetus/ not (exp fetus/ and exp adult/)
23 exp newborn/ not (exp newborn/and exp adult/)
24 19 not (20 or 21 or 22 or 23) [CHILD REMOVED]
25 ("case report" or "case series" or "case presentation" or "single-case study" or "randomi*ed control* trial" or "RCT" or "placebo" or "drug therapy").ti.
26 24 not 25 [study design removed]
27 (("a tertiary" adj2 (hospital or facility or centre or center or "care center" or "care centre")) or ("a referral" adj2 (hospital or facility or center or centre or "care center" or "care centre"))).ti.
28 26 not 27 [single hospital studies removed]
29 (australia* or mauritius* or antigua* or barbuda* or argentin* or aruba* or brazil* or chile* or "Costa Rica" or colombia* or cuba* or dominica* or ecuador* or grenada* or guatemala* or mexico* or montserrat* or nicaragua* or paraguay* or peru* or "Saint Lucia" or "Saint Vincent and Grenadines" or "United States of America" or uruguay* or cyprus* or Israel* or Japan* or Malaysia* or mongolia* or oman* or "Republic of Korea" or Singapore* or Armenia* or Andorra* or Austria* or "Bosnia and Herzegovina" or Bulgaria* or "Czech

Republic" or Czechia* or denmark* or Estonia* or finland* or georgia* or germany* or Greece* or Iceland* or Kazakhstan* or Latvia* or Lithuania* or Luxembourg* or Macedonia* or malta* or netherlands* or Poland* or serbia* or "San Marino" or Slovenia* or Spain* or Switzerland* or "United Kingdom" or England* or "England and Wales" or Wales* or "Northern Ireland" or scotland*).tw,kf.
30 28 not 29 [CRVS COUNTRIES REMOVED] 24309

Global Index Medicus

\# Searches Results
1 (mh:("Maternal Death" OR "Fatal Outcome" OR Suicide OR Homicide) OR (mortal OR mortality OR mortalities OR dead OR death OR deaths OR decease* OR demise* OR died OR dies OR dying OR fatal OR fatality OR fatalities OR non-survival* OR nonsurvival* OR not-surviv* OR suicid* OR homicid* OR kill OR killed OR kills OR killing OR murder*)) 200'201
2 (mh:(Pregnancy OR "Pregnancy Complications" OR "Pregnant Women" OR "Pregnancy Trimesters" OR "Postpartum Period" OR "Maternal Health" OR "Abortion, Criminal" OR "Vacuum Curettage" OR "Abortifacient Agents") OR (pregnant OR pregnanc* OR parturition* OR childbirth* OR "child birth" OR "abdominal birth"~3 OR "vaginal birth"~3 OR "abdominal delivery"~3 OR "vaginal delivery"~3 OR caesarean* OR cesarean* OR "c-section" OR "c-sections" OR maternal* OR maternit* OR mother* OR postpartum* OR "post-partum" OR miscarr* OR abortion* OR postabort* OR "post-abort" OR "dilatation curettage"~2 OR "dilation curettage"~2 OR "vacuum curettage"~2 OR "suction curettage"~2 OR "uterus aspiration"~2 OR "uteri aspiration"~2 OR "uterine aspiration"~2 OR "vacuum aspiration"~2 OR "dilatation evacuation"~2 OR "dilation evacuation"~2)) 141'746
3 (mh:("Maternal Mortality" OR "Cause of Death" OR Mortality OR "Hospital Mortality" OR Autopsy OR "Death Certificates" OR "Data Collection" OR "Epidemiologic Factors" OR "Epidemiological Monitoring" OR Incidence OR Prevalence OR "Databases, Factual" OR "Survival Rate" OR "Vital Statistics") OR (autops* OR "death certificate" OR incidenc* OR prevalen* OR "clinical database" OR "disease database" OR "factual database" OR register OR registers OR registry OR registries OR surveillance* OR vital statistic* OR "vital registration" OR "death burden"~3 OR "death data"~3 OR "death estimate"~3 OR "death rate"~3 OR "mortality burden"~3 OR "mortality data"~3 OR "mortality estimate"~3 OR "mortality rate"~3 OR "fatality ratio"~3 OR "fatality rate"~3 OR "fatality burden"~3 OR "fatality estimate"~3 OR "fatality data"~3 OR "death reporting"~3 OR "death report"~3 OR "death underreporting"~3 OR "mortality report"~3 OR "mortality reporting"~3 OR "mortality underreporting"~3 OR "mortality statistic"~3 OR "death statistic"~3 OR "fatality statistic"~3 OR "suicide rate"~3 OR "suicide statistic"~3 OR "suicide ratio"~3 OR "suicide burden"~3 OR "suicide estimate"~3 OR "suicide data"~3 OR "suicide report"~3 OR "Reproductive-Age-Mortality-Study" or "cause-specific-mortality-fraction" OR "confidential inquiry"~3 OR "confidential enquiry"~3)) 599'081
4 entry_date:([20171215 TO 20231231])
5 1 AND 2 AND 3 AND 4 ~2000
6 ti:(children or neonat* or "child mortality" or congenital or newborn*)
7 5 AND NOT 6

Combined ((tw:((mh:("Maternal Death" OR "Fatal Outcome" OR Suicide OR Homicide) OR (mortal OR mortality OR mortalities OR dead OR death OR deaths OR decease* OR demise* OR died OR dies OR dying OR fatal OR fatality OR fatalities OR non-survival* OR nonsurvival* OR not-surviv* OR suicid* OR homicid* OR kill OR killed OR kills OR killing OR murder*)))) AND (tw:((mh:(Pregnancy OR "Pregnancy Complications" OR "Pregnant Women" OR "Pregnancy Trimesters" OR "Postpartum Period" OR "Maternal Health" OR "Abortion, Criminal" OR "Vacuum Curettage" OR "Abortifacient Agents") OR (pregnant OR

pregnanc* OR parturition* OR childbirth* OR "child birth" OR "abdominal birth"~3 OR "vaginal birth"~3 OR "abdominal delivery"~3 OR "vaginal delivery"~3 OR caesarean* OR cesarean* OR "c-section" OR "c-sections" OR maternal* OR maternit* OR mother* OR postpartum* OR "post-partum" OR miscarr* OR abortion* OR postabort* OR "post-abort" OR "dilatation curettage"~2 OR "dilation curettage"~2 OR "vacuum curettage"~2 OR "suction curettage"~2 OR "uterus aspiration"~2 OR "uteri aspiration"~2 OR "uterine aspiration"~2 OR "vacuum aspiration"~2 OR "dilatation evacuation"~2 OR "dilation evacuation"~2)))) AND (tw:((mh:("Maternal Mortality" OR "Cause of Death" OR Mortality OR "Hospital Mortality" OR Autopsy OR "Death Certificates" OR "Data Collection" OR "Epidemiologic Factors" OR "Epidemiological Monitoring" OR Incidence OR Prevalence OR "Databases, Factual" OR "Survival Rate" OR "Vital Statistics") OR (autops* OR "death certificate" OR incidenc* OR prevalen* OR "clinical database" OR "disease database" OR "factual database" OR register OR registers OR registry OR registries OR surveillance* OR vital statistic* OR "vital registration" OR "death burden"~3 OR "death data"~3 OR "death estimate"~3 OR "death rate"~3 OR "mortality burden"~3 OR "mortality data"~3 OR "mortality estimate"~3 OR "mortality rate"~3 OR "fatality ratio"~3 OR "fatality rate"~3 OR "fatality burden"~3 OR "fatality estimate"~3 OR "fatality data"~3 OR "death reporting"~3 OR "death report"~3 OR "death underreporting"~3 OR "mortality report"~3 OR "mortality reporting"~3 OR "mortality underreporting"~3 OR "mortality statistic"~3 OR "death statistic"~3 OR "fatality statistic"~3 OR "suicide rate"~3 OR "suicide statistic"~3 OR "suicide ratio"~3 OR "suicide burden"~3 OR "suicide estimate"~3 OR "suicide data"~3 OR "suicide report"~3 OR "Reproductive-Age-Mortality-Study" or "cause-specific-mortality-fraction" OR "confidential inquiry"~3 OR "confidential enquiry"~3)))) entry_date:([20171215 TO 20231231])) AND NOT ti:(children or neonat* or "child mortality" or congenital or newborn*)   1680

Figure S4 PRISMA for Search 2

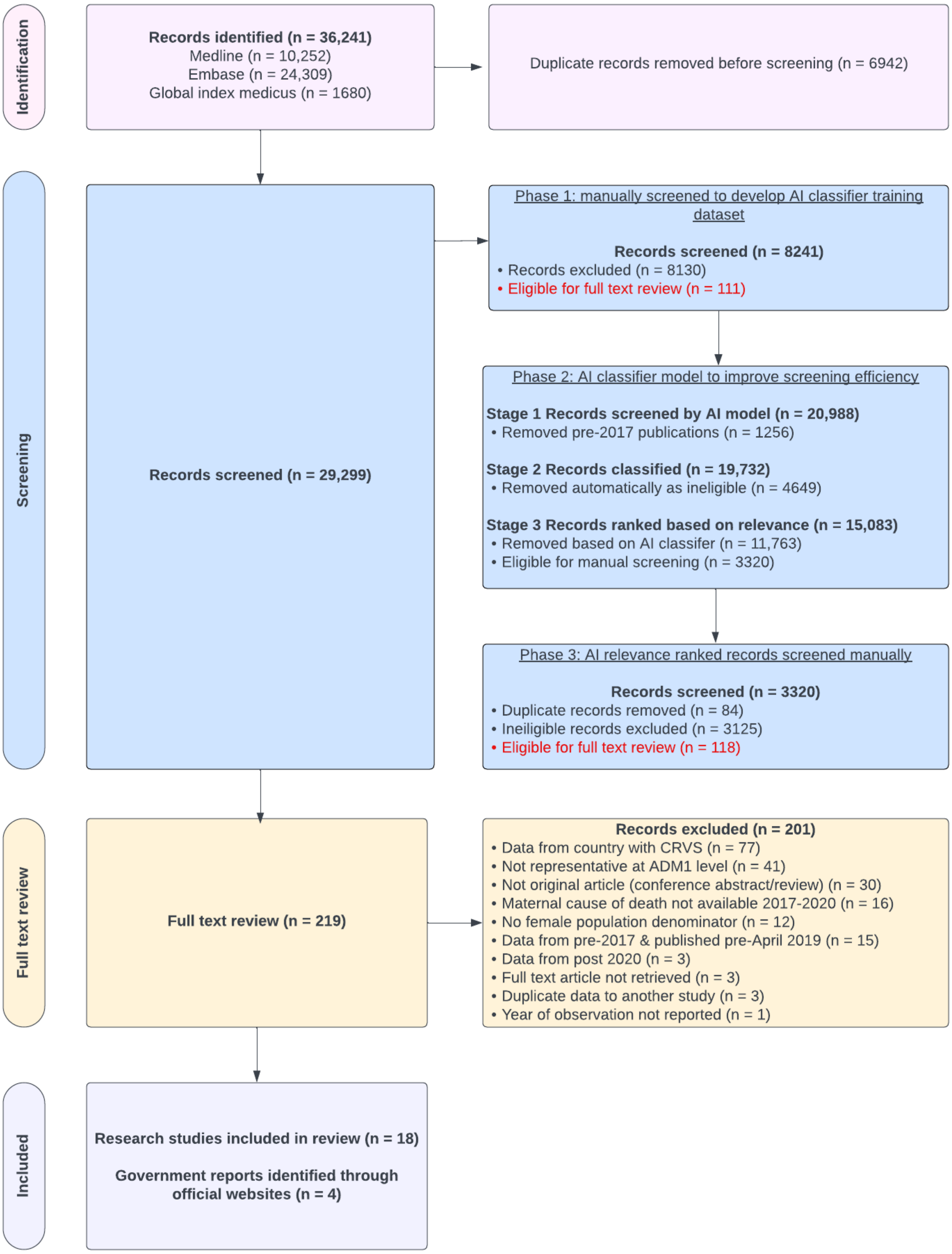

APPENDIX 3: ARTIFICIAL INTELLIGENCE CLASSIFIER MODEL

We used an artificial intelligence classifier model to improve the efficiency of the bibliographic search for 2017-2020 data. Development and use of this AI classifier involved the following steps:

1. From records identified from bibliographic databases, prior to title and abstract screening, the classifier was used to automatically eliminate pre-2017 studies and those with ineligible study designs.
2. Roughly 9000 records were manually screened based on title and abstract to develop a training dataset.
3. Using this training dataset, the AI model classified the remaining records into five categories of relevance (from most to least relevant): 1,2,3,4 or 5 stars. Articles attributed 3 stars had a 99.4% exclusion accuracy. Records with 3 stars or higher were therefore excluded.
4. The AI model then classified the remaining records into batches that were ranked on their relevance based on subject-specific keywords (1. Cause of death, 2. Maternal death/maternal mortality/pregnancy-related mortality/pregnancy-associated death/maternal outcome; 3. International classification of diseases/ ICD-MM/ ICD-10/ICD-11; 4. Cause-specific mortality/ cause-specific mortality fraction (CSMF).
5. Batches were ranked into deciles based on their keyword relevance: 90-100, 80-90, 70-80, etc. based on their relevance score. Manual screening began starting with the highest relevance batch (90-100). As soon as a batch contained no records eligible for full-text review, we assumed batches with a lower relevance score were also irrelevant and these records were excluded.
6. Eligible records proceeded to manual full-text review.

APPENDIX 4: PROCESS FOR ASSIGNING DEATHS TO AN ICD CODE OR GROUPING WHERE THE CAUSE OF DEATH WAS REPORTED IN FREE TEXT FORMAT

All data from the WHO Mortality Database was directly reported as an ICD code; among the grey and peer-reviewed literature sources, 342 (69%) reported cause of death directly as a numeric ICD code; 14 (3%) reported a high-level ICD group; 62 (13%) reported the cause of death in free-text format; 76 (15%) reported a combination. Where the cause of death existed in a free-text format, the relevant ICD code was assigned.[1] The assignment of ICD codes was completed by a single author (HL) with an OBGYN with advanced training in maternal fetal medicine. Queries were initially discussed with JC and DC; with inputs and guidance sought from the scientific technical advisory group, as appropriate.

Wherever possible, a single ICD cause was assigned. For example, “amniotic fluid embolism” was coded as O88.1; “ectopic pregnancy” was coded as O00. Wherever possible, the full 3-digit ICD code was applied; where the level of detail reported did not allow for this a 2-digt code or the relevant ICD group was assigned. For example, “early pregnancy deaths” without further descriptive information were coded as “ICD 10 Group 1: Pregnancy with abortive outcome”, or “preeclampsia-eclampsia” deaths, without further description was coded as ICD 10 Group 2: Hypertensive disorders.

In cases where there was uncertainty, multiple codes were assigned with relative probability always adding up to 100%. (6% of assignments). When the original source was in English, the full text of the original source was reviewed in detail to determine what additional information could be identified to aid in ICD code assignment. For example, deaths extracted as “haemorrhage” where the original report noted that 75% of hemorrhage deaths occurred postpartum. In this instance, “haemorrhage” would be coded as “O72 (75%)” and “Haemorrhage NOS [Not Otherwise Specified] (25%)”. Original text was reviewed for further details particularly where the following free-text labels were used: “abortion”, “hypertension”, “preeclampsia/eclampsia”, “haemorrhage”, “anaesthesia death”, “other infection”, “HIV/AIDS”.

For deaths that the primary source had identified as either ”Direct Obstetric”, “Indirect Obstetric” or “non obstetric” the text was examined to determine if additional detail could be obtained for coding. In the absence of additional specific deaths these deaths were coded as either “or “Direct NOS” or “Indirect NOS”

In effort to minimize the introduction of bias, deference was given to the primary source whenever possible if the extracted text included identification of the cause as being due to a “direct” or “indirect” obstetric cause. For example a death described as “indirect-cardiac death” would be coded under Group 7 Non Obstetric Complications as O99.4. In circumstances where the primary coder felt the text designation of direct or indirect could not be clinically correlated these deaths were coded according to the reported cause and the provided text of ‘direct’ or ‘indirect’ was disregarded. An example of this would be a reported free text cause of “Indirect death- obstetric sepsis”, which was subsequently coded

---

[1] World Health Organization. *The WHO Application of ICD-10 to Deaths during Pregnancy, Childbirth and the Puerperium: ICD-MM*. World Health Organization; 2012. https://apps.who.int/iris/bitstream/handle/10665/70929/9789241548458_eng.pdf.

as “O85 Puerperal Sepsis” a direct obstetric death. A list of these circumstances was kept as they arose by the primary coder and periodically reviewed by JC + DC to ensure consistency and minimize bias.

In effort to minimize the introduction of bias, deference was given to the primary source whenever possible when the extracted text modified the cause as being “direct/indirect”. In circumstances where HL felt the modifier did not correlate clinically to the cause reported, the designation of “direct” or “indirect” was disregarded. An example of this is “indirect death-obstetric sepsis” coded as O85 Puerperal Sepsis.

-Deaths that are ill defined as to cause (ex: shock, organ failure, cardiac arrest, etc) are coded as ‘R’ with an additional organ system code if that information can be determined (ex: R-pulmonary vs R-renal vs R-NOS).

<u>Assumptions</u>

-”aspiration” was coded as Anaesthesia Group 6
-all cancer deaths (malignancy, indirect malignancy, neoplasm etc) with the exception of gestational trophoblastic disease were coded with Group 7

<u>Country Specific Assumptions</u>

China:

The following decisions were made based on conversation with Chinese reviewers
-“Internal + Surgical diseases complicating pregnancy etc” coded as Group 7
-“Cancer complicating pregnancy/childbirth etc” coded as generic cancer – not GTD
-“Tumor in reproductive system” or “tumor” both coded as generic cancer
-“Internal diseases complicating pregnancy etc” coded as Group 7
-“Diseases complicating pregnancy, childbirth etc” coded as DIRECT NOS \

South Africa:

For South Africa, in order to better assess the impact of HIV/AIDs among reported TB deaths, deaths coded as TB for this country were adjusted in the model as described in the methods.

<u>Cause Specific Assumptions</u>

<u>Cause Specific Direct Deaths Assumptions</u>

- “medication reactions” coded as DIRECT NOS
-“anaphylactic reactions” coded as DIRECT NOS
-“transfusion reactions” coded as “DIRECT NOS”
- “direct obstetric” coded as “DIRECT NOS”
-“Sudden death/Acute collapse” with no other info coded as DIRECT NOS
-“preterm premature rupture of membranes” were coded as DIRECT NOS
-“ multiple gestation/twins” coded as DIRECT NOS
- “obstetric pathology” coded as DIRECT NOS
-anesthesia deaths that referred to/suggested a complication from a spinal were coded as O74.6

- “hypovolemia” coded as Haemorrhage NOS
-“liver disease” coded as O26.6 if there was descriptive information supporting Preeclampsia/Acute Fatty Liver of Pregnancy.
-“sepsis” without additional information were coded as O85 Puerperal Sepsis
-“retained placenta” coded as O73 (50%) and O72 (50%)
-“cardiac failure” coded as O90.3
-“cesarean section complications” coded as O75.4
-“cephalopelvic disproportion” coded as O66.9
- “uterine rupture NOS” coded as O71.9
-“deep vein thrombosis/pulmonary embolism/thromboembolism” coded as O88
-“cerebral vascular accident coded as Group 2
- “non-traumatic intracranial haemorrhage” coded as Group 2
-“stroke” coded as Group 2
-deaths reported as being due to both abruption and hypertension were coded as Group 2
-“DIC” coded as Group 2 (50%) and Hemorrhage (50%)
- “malignancy (direct)” coded as C58

Cause Specific Indirect Deaths Assumptions
- “indirect sepsis (influenza)” coded as O98.5
- “infection (excluding HIV and hepatitis B)” coded as O98.5
- “COVID-19” coded as O98.9
- “liver disease” without any additional information coded as O99.6
-“renal disease NOS” coded as O99.8
-“cardiac disease” coded as O99.4 unless there was information available to suggest cardiomyopathy, in which case it was coded as O90.3
- “ruptured splenic artery aneurysm” was coded as O99.4
-“heart valve mechanical prosthesis” was coded as O99.4
-“malformation of brain vessels” was coded as O99.4
- “indirect neurological conditions” was coded as O99.3
-“pneumonia” coded as O98.9 ‘unspecified maternal infection’
-“thrombotic thrombocytopenia purpura” coded as O99.1
-“thrombotic microangiopathy” coded as O99.1
-“non-pregnancy related UTI” coded as O23.1 (50%) and Group 7 (50%)
-“psychiatric causes – drugs/alcohol/others” coded as Group 7
-“ductal carcinoma” coded as Group 7
- “malignant neoplasm of nipple and areola” coded as Group 7
- “neoplasm of uncertain behaviour of brain” coded as Group 7
-“myxofibrosarcoma with lung metastasis” coded as Group 7

HIV/AIDS

For deaths listed as being caused by AIDS defining lesions, these were coded as O98.7. This includes cryptococcus, PCP pneumonia, Kaposi’s sarcoma, wasting disease and complications of antiretroviral therapy.

Timing of Deaths

-deaths reported as occurring on the day of delivery were grouped with postpartum with the exception of those deaths due to Amniotic Fluid Embolism

Table S3 Classification of deaths by ICD codes

Group Name		ICD Codes
Pregnancy with abortive outcome (ABO)		O00, O00.0, O00.1, O00.2, O00.8, O00.9, O01, O01.0, O01.1, O01.9, O02, O02.0, O02.1, O02.8, O02.9, O03, O03.0, O03.1, O03.2, O03.3, O03.4, O03.5, O03.6, O03.7, O03.8, O03.9, O04, O04.0, O04.1, O04.3, O04.5, O04.6, O04.7, O04.8, O04.9, O05, O05.0, O05.1, O05.2, O05.3, O05.4, O05.5, O05.6, O05.7, O05.8, O05.9, O06, O06.0, O06.1, O06.2, O06.3, O06.4, O06.5, O06.6, O06.7, O06.8, O06.9, O07, O07.0, O07.1, O07.2, O07.3, O07.4, O07.5, O07.6, O07.7, O07.8, O07.9
Hypertensive disorders in pregnancy, childbirth and the puerperium (HYP)		O11, O12, O12.0, O12., O12.2, O13, O14, O14.0, O14.1, O14.2, O14.9, O15, O15.0, O15.1, O15.2, O15.9, O16
Obstetric haemorrhage (HEM)	Antepartum	O20, O20.0, O20.8, O20.9, O44, O44.1, O45, O45.0, O45.8, O45.9, O46, O46.0, O46.8, O46.9, O71.0
	Intrapartum	O43, O43.2, O67, O67.0, O67.8, O67.9, O71.1, Rupture NOS
	Postpartum	O72, O72.0, O72.1, O72.2, O72.3
	Timing Unknown	O71.3, O71.4, O71.7, Hemorrhage NOS
Pregnancy-related infection (SEP)	Antepartum	O23, O23.0, O23.1, O23.2, O23.3, O23.4, O23.5, O23.9, O41.1
	Intrapartum	O75.3
	Postpartum	O85, O86, O86.0, O86.1, O86.2, O86.3, O86.4, O86.8, O91, O91.1, O91.2
	Timing Unknown	A34
Obstetric embolism (EMB)		O22, O22.3, O22.5, O22.8, O22.9, O87, O87.1, O87.3, O87.9, O88, O88.0, O88.1, O88.2, O88.3, O88.8, O87.0, O87.2, O87.8
Other direct causes * (DIR)	Anaesthesia	O29, O29.0, O29.1, O29.2, O29.3, O29.5, O29.6, O29.8, O29.9, O74, O74.0, O74.1, O74.2, O74.3, O74.4, O74.6, O74.7, O74.8, O74.9, O89, O89.0, O89.1, O89.2, O89.5, O89.6, O89.8, O89.9, O74.5, O89.4
	Obstetric trauma	O71.2, O71.5, O71.6, O71.8, O71.9
	Obstructed labour	O33, O33.0, O33.3, O33.4, O33.5, O33.9, O62, O62.0, O62.1, O62.2, O62.3, O62.4, O62.8, O62.9, O63, O63.0, O63.1, O63.2, O63.9, O64, O64.0, O64.1, O64.2, O64.4, O64.5, O64.8, O64.9, O65, O65.1, O65.4, O65.5, O65.9, O66, O66.0, O66.1, O66.2, O66.3, O66.4, O66.9
	Other	O21.1, O24.4, O26.6, O44.0, O73, O73.0, O73.1, O75.4, O75.8, O75.9, O90, O90.0, O90.1, O90.2, O90.3, O90.4, O90.5, O90.8, O90.9, C58, O21,

		O21.0, O21.9, O22.0, O22.1, O22.2, O25, O26, O26.0, O26.1, O26.3, O26.5, O26.8, O26.9, O28, O28.5, O28.8, O30, O30.0, O30.1, O30.9, O31, O31.2, O31.8, O32, O32.1, O32.2, O32.4, O32.8, O32.9, O34, O34.0, O34.1, O34.2, O34.3, O34.4, O34.5, O34.6, O34.8, O34.9, O35, O35.0, O35.1, O35.5, O35.8, O35.9, O36, O36.0, O36.1, O36.2, O36.3, O36.4, O36.5, O36.6, O36.7, O36.8, O36.9, O40, O41, O41.0, O41.8, O41.9, O42, O42.0, O42.1, O42.2, O42.9, O43.0, O43.1, O43.8, O43.9, O47.0, O47.9, O48, O60, O60.0, O60.1, O60.2, O60.3, O61, O61.0, O61.9, O68, O68.0, O68.1, O68.2, O68.8, O68.9, O69, O69.0, O69.1, O69.2, O69.4, O69.5, O69.8, O69.9, O70, O70.0, O70.1, O70.2, O70.3, O70.9, O75, O75.0, O75.1, O75.2, O75.6, O75.7, O92, O92.2, O92.3, O92.5, O92.6
Non-obstetric complications (indirect maternal deaths) (IND)		O10, O10.0, O10.1, O10.2, O10.3, O10.4, O10.9, O24, O24.0, O24.1, O24.2, O24.3, O24.9, O98, O98.0, O98.1, O98.3, O98.4, O98.5, O98.6, O98.8, O98.9, O99.0, O99.1, O99.2, O99.3, O99.4, O99.5, O99.6, O99.7, O99.8

NOS: Not otherwise specified

* Note that this grouping corresponds to both *Group 5 Other obstetric complications* and *Group 6: Unanticipated complications of management* within ICD-MM combined, minus those deaths separately classified as *Obstetric embolism (EMB).* Deaths categorised within *Group 6: Unanticipated complications of management* have been presented as a sub-category (*Anaesthesia*) within this group.

APPENDIX 5: DETAILS OF THE STATISTICAL MODEL

1 Model overview

Observed non-HIV/AIDS cause-specific death counts for each country are grouped into 7 main cause of death categories (abortion (ABO), embolism (EMB), hemorrhage (HEM), sepsis (SEP), hypertension (HYP), other direct causes (DIR), and indirect causes (IND)). Statistically, these death counts are treated as coming from a multinomial distribution where the relative proportions of deaths by cause depend primarily on regional and country-level differences. An additional error term for lower quality data mitigates the effect of potentially misrepresentative observations.

The DIR, HEM, and SEP categories are further divided into separate multinomial sub-distributions and are similarly modeled as the sum of regional and country-level differences.

In order to reflect increased uncertainty in countries where data coverage is low, the weight given to the country's specific estimated term depends on the coverage of the available data in that country.

2 Model for observed cause proportions

Let d_i denote the total number of non-HIV/AIDS maternal deaths in the ith observation, for $i = 1, \ldots, N$. Let $\mathbf{y_i} = (y_{i,1}, \ldots, y_{i,7})$ be the corresponding non-HIV/AIDS cause-specific counts, where $y_{i,j}$ is the number of deaths in cause group j, for $j \in$ {ABO, EMB, HEM, SEP, DIR, IND, HYP}.

The corresponding multinomial proportions $(\phi_{i,1}, \ldots, \phi_{i,7})$ are modeled as

$$\mathbf{y_i} \sim \text{Multinomial}(d_i, \boldsymbol{\phi}_\mathbf{i})$$
$$\boldsymbol{\phi}_\mathbf{i} = (\phi_{i,1}, \ldots, \phi_{i,7})$$
$$\log\left(\frac{\phi_{i,j}}{\phi_{i,7}}\right) = \alpha_j + \beta_{r[c[i]],j} + \gamma_{c[i],j} + \delta_{i,j}$$

where $c[i]$ refers to country of observation i, and $r[c[i]]$ corresponds to the region of that country. The log-ratio of proportions for category j relative to the baseline, category 7 (HYP), depends on an intercept term α_j, a region effect $\beta_{r[c[i]],j}$, a country effect $\gamma_{c[i],j}$ and a data quality adjustment term $\delta_{i,j}$.

For all regions $r = 1, \ldots, R$, the region effects are pooled

$$\beta_{r,j} \sim \text{Normal}(0, \sigma_\beta^2)$$
$$\sigma_\beta \sim \text{Normal}(0, 1^2).$$

This region classification used here is the same as Say et al. (2014), which groups epidemiologically similar countries. Details on the modeling region classification are given in Table S4 below.

Table S4 Bayesian hierarchical model regional classification

Model region	Countries
Central Asia	Kazakhstan; Kyrgyzstan; Tajikistan; Turkmenistan; Uzbekistan
Eastern Asia	China; Democratic People's Republic of Korea; Mongolia; Republic of Korea
Eastern Africa	Burundi; Comoros; Djibouti; Eritrea; Ethiopia; Kenya; Madagascar; Malawi; Mauritius; Mozambique; Rwanda; Seychelles; Somalia; South Sudan; Sudan; United Republic of Tanzania; Uganda; Zambia; Zimbabwe
South-Eastern Asia / Oceania	Brunei Darussalam; Cambodia; Fiji; Indonesia; Kiribati; Lao People's Democratic Republic; Malaysia; Micronesia (Federated States of); Myanmar; Papua New Guinea; Philippines; Samoa; Singapore; Solomon Islands; Thailand; Timor-Leste; Tonga; Vanuatu; Viet Nam
Southern Africa	Botswana; Lesotho; Namibia; South Africa; Eswatini
Western Africa	Benin; Burkina Faso; Cabo Verde; Côte d'Ivoire; Gambia; Ghana; Guinea; Guinea-Bissau; Liberia; Mali; Mauritania; Niger; Nigeria; Senegal; Sierra Leone; Togo
Central America	Belize; Costa Rica; El Salvador; Guatemala; Honduras; Mexico; Nicaragua; Panama
Developed regions	Australia; Austria; Belarus; Belgium; Bulgaria; Canada; Czechia; Denmark; Estonia; Finland; France; Germany; Greece; Hungary; Iceland; Ireland; Italy; Japan; Latvia; Lithuania; Luxembourg; Malta; Netherlands; New Zealand; Norway; Poland; Portugal; Republic of Moldova; Romania; Russian Federation; Slovakia; Slovenia; Spain; Sweden; Switzerland; Ukraine; United Kingdom of Great Britain and Northern Ireland; United States of America
Western Asia	Armenia; Azerbaijan; Bahrain; Cyprus; Georgia; Iraq; Israel; Jordan; Kuwait; Lebanon; West Bank and Gaza Strip; Oman; Qatar; Saudi Arabia; Syrian Arab Republic; Turkey; United Arab Emirates; Yemen
South America	Argentina; Bolivia (Plurinational State of); Brazil; Chile; Colombia; Ecuador; Guyana; Paraguay; Peru; Suriname; Uruguay; Venezuela (Bolivarian Republic of)
Caribbean	Antigua and Barbuda; Bahamas; Barbados; Cuba; Dominican Republic; Grenada; Haiti; Jamaica; Puerto Rico; Saint Lucia; Saint Vincent and the Grenadines; Trinidad and Tobago
Middle Africa	Angola; Cameroon; Central African Republic; Chad; Congo; Democratic Republic of the Congo; Equatorial Guinea; Gabon; Sao Tome and Principe
Northern Africa	Algeria; Egypt; State of Libya; Morocco; Tunisia
Transition countries of South-Eastern Europe	Albania; Bosnia and Herzegovina; Croatia; Montenegro; Serbia; Republic of North Macedonia

Model region	Countries
Southern Asia	Afghanistan; Bangladesh; Bhutan; India; Iran (Islamic Republic of); Maldives; Nepal; Pakistan; Sri Lanka

3 Multinomial likelihood and missing values

When the count in some category k is missing, we wish to treat $y_{i,k}$ as unknown instead. In these cases, we treat the observation as coming from a multinomial distribution with these categories removed. For example, if the count in observation i for ABO ($j = 1$) and EMB ($j = 2$) are missing, then $y_{i,1}$ and $y_{i,2}$ are discarded, and $y_{i,3}, \dots, y_{i,7}$ are modeled with a multinomial with the remaining 5 categories with proportions rescaled to add to 1.

4 Country-specific effect and correlations across causes

The country effects $\gamma_{c,j}$ are modeled as zero-mean multivariate normal with a common covariance matrix Σ, which allows for the possibility of correlations between death categories. For each country $c = 1, \dots, C$, the country effect is modeled

$$(\gamma_{c,1}, \dots, \gamma_{c,6}) \sim \mathrm{MVN}(\mathbf{0}, \Sigma)$$
$$\Sigma = \mathrm{diag}(\boldsymbol{\tau})\Omega\mathrm{diag}(\boldsymbol{\tau})$$
$$\Omega \sim \mathrm{LKJ}(1)$$
$$\boldsymbol{\tau} \sim \mathrm{MVN}(\mathbf{0}, \mathbf{I}_{6\times 6})$$

where $\Sigma = \mathrm{diag}(\boldsymbol{\tau})\Omega\mathrm{diag}(\boldsymbol{\tau})$ is the decomposition of the covariance matrix into variance terms and its correlation matrix Ω, and an LKJ prior is placed on Ω (Lewandowski, Kurowicka, and Joe 2009; Stan Development Team 2019).

5 Data-quality adjustment

We add an additional error term for low quality data that mitigates the effect of these observations on the estimates.

Observations are treated differently according to the nature of the source and then according to several data quality indicators. For each observation i, the data quality terms for $j = 1, \dots, 6$ are modelled

$$\delta_{i,j} \begin{cases} = 0 & \text{for observations from high-quality government reports} \\ \sim N(0, \sigma^2_{\delta,i,j}) & \text{for CRVS and studies observations.} \end{cases}$$

Observations from government reports are assumed to be of the highest quality and the δ terms for these are set to zero. Otherwise, the variance terms are defined as

$$\sigma_{\delta,i,j} = \begin{cases} (1 - u_i)\sigma_{\delta,j} & \text{for CRVS observations} \\ \sigma_{\delta,j} & \text{for studies.} \end{cases}$$

For CRVS observations, the “usability index” u_i takes values between 0 and 1 and scales the variance term according to the data quality indicators of the CRVS observation. Studies are assumed to be of the lowest quality, and are subject to the full variance. The calculation of u_i is similar to the one calculated by Say et al. (2014). Let C_i denote the all-cause (maternal and otherwise) female death coverage of the CRVS observation, let p_i^{ill} denote the proportion of ill-defined deaths, and let $\mathring{d}_{ct}$ denote the MMEIG estimate of the number of non-HIV/AIDS maternal deaths in country c and year t. Then for an observation where the number of observed deaths is no more than 5, the usability index is calculated

$$u_i = C_i(1 - p_i^{\text{ill}}).$$

For observations where the observed number of deaths is greater than 5, we also consider the proportion p_i^{contr} of maternal deaths attributed to contributory causes which indicates CRVS systems of lower quality. In these cases, the usability index is calculated

$$u_i = C_i(1 - p_i^{\text{ill}})(1 - p_i^{\text{contr}}).$$

6 Estimation of true proportions

Recorded deaths may not be representative of all the maternal deaths in that country-year, which we take to be the MMEIG point estimate denoted $\mathring{d}_{ct}$, resulting in overly-certain estimates of underlying proportions. We therefore apply a weighting scheme to better reflect uncertainty from data coverage. For country c, define $w_c \in [0,1]$ to be the maximum coverage among its observations. If $ct[i]$ refers to the country-year of observation i, then

$$w_c := \min\left\{1, \max_{i:c[i]=c} \frac{d_i}{\mathring{d}_{ct[i]}}\right\},$$

where d_i is the total number of observed maternal deaths from data source i. The estimate of the true HIV/AIDS-omitted cause of death distribution is calculated by taking a weighting of the estimated country effect for country c and a new realization of the country effect, denoted $\tilde{\gamma}_c$.

$$\log\left(\frac{\hat{\phi}_{c,j}}{\hat{\phi}_{c,7}}\right) = \alpha_j + \beta_{r[c],j} + w_c\gamma_{c,j} + (1 - w_c)\tilde{\gamma}_{c,j}$$

$$\tilde{\boldsymbol{\gamma}}_c \overset{\text{RNG}}{\sim} \text{MVN}(\mathbf{0}, \Sigma).$$

7 Incorporating HIV/AIDS deaths

Deaths from HIV/AIDS are treated separately. Let $\mathring{d}_{ct,\text{HIV}}$ denote the MMEIG point estimate of the number of HIV/AIDS deaths in country-year ct.

We add the HIV/AIDS deaths $\mathring{d}_{ct,HIV}$ to the to the IND group, and recalculate proportions to obtain the final HIV/AIDS-inclusive country-year distributions $(\hat{\phi}_{ct,1}, \dots, \hat{\phi}_{ct,7})$. We do this by converting the HIV/AIDS-omitted proportions $\hat{\phi}_{c,j}$ into counts $\hat{d}_{ct,j}$, adding the estimated HIV/AIDS counts appropriately, and rescaling these counts into proportions:

$$\hat{d}^*_{ct,j} = \hat{\phi}_{c,j} \cdot \mathring{d}_{ct}$$

$$\hat{d}^*_{ct,j} = \begin{cases} \hat{d}_{ct,j} + \mathring{d}_{ct,\text{HIV}} & \text{if } j = \text{IND} \\ \hat{d}_{ct,j} & \text{otherwise} \end{cases}$$

$$\hat{\phi}^*_{ct,j} = \frac{\hat{d}^*_{ct,j}}{\sum_{k=1}^{7} \hat{d}^*_{ct,k}}.$$

8 Calculating regional and global cause of death distributions

Let $h[c]$ denote the SDG region of country c. For (SDG) regions $h = 1, \dots, H$, the countries' HIV/AIDS-inclusive counts $\hat{d}^*_{ct,j}$ are aggregated accordingly to obtain regional counts for each cause, then normalized to give proportions

$$\hat{d}^*_{h,j} = \sum_{h[c]=h} \hat{d}^*_{ct,j}$$

$$\hat{\phi}^*_{h,j} = \frac{\hat{d}^*_{h,j}}{\sum_{k=1}^{7} \hat{d}^*_{h,l}}.$$

Global estimates are obtained similarly by aggregating regional death counts

$$\hat{d}^*_{\text{global},j} = \sum_{h=1}^{H} \hat{d}^*_{h,j}$$

$$\hat{\phi}^*_{\text{global},j} = \frac{\hat{d}^*_{\text{global},j}}{\sum_{k=1}^{7} \hat{d}^*_{\text{global},k}}.$$

9 Subcause distribution estimation

Within the cause groups HEM, SEP, and DIR, we further divide these into K_j subcategories as follows.

1. Within the hemorrhage (HEM) category, deaths can be classified as antepartum (HEM), intrapartum (HEM), and postpartum (HEM) hemorrhage. $K_{\text{HEM}} = 3$.
2. The sepsis (SEP) category can similarly be divided into antepartum (SEP), intrapartum (SEP), and postpartum sepsis (SEP). $K_{\text{SEP}} = 3$
3. The direct cause (DIR) category can be divided into obstructed labour (DIR), obstetric trauma (DIR), anaesthesia (DIR), and other direct causes (DIR). $K_{\text{DIR}} = 4$.

The estimation of subcategories is similar to that of the main categories' procedure, except that the data quality term δ is not used.

10 Computation in Stan

Posterior samples were obtained using Hamiltonian Monte Carlo implemented in Stan via the *cmdstanr* R package with 4 parallel chains of 3000 warmup iterations and 2000 sampling iterations (Stan Development Team 2019; Gabry and Češnovar 2020). Standard checks for $\hat{R}$ and effective sample size were performed. This computation was enabled in part by resources provided by Compute Ontario (https://computeontario.ca/) and ComputeCanada (https://www.computecanada.ca/).

11 Validation

Several model-checking exercises were performed. We checked the coverage of posterior predictive intervals in three different cross-validation exercises, leaving out (1) studies data, (2) data from "Developed regions" as defined in the modelling region specification, and (3) a random 20% subset of observations, and find that the uncertainty in the model is somewhat conservative (wider than nominal) in predicting category-specific counts. We also studied the performance of the model with respect to misclassification, choice of the baseline category, and missing categories in observations. Details are given in Alexander, Chong, and Pejcinovska (2021).

References

Alexander, M., Chong, M. Y., & Pejcinovska, M. (2021). Estimating causes of maternal death in data-sparse contexts. arXiv preprint arXiv:2101.05240.

Gabry, Jonah, and Rok Češnovar. 2020. *Cmdstanr: R Interface to 'CmdStan'*.

Lewandowski, Daniel, Dorota Kurowicka, and Harry Joe. 2009. "Generating Random Correlation Matrices Based on Vines and Extended Onion Method." *Journal of Multivariate Analysis* 100 (9): 1989–2001.

Say, Lale, Doris Chou, Alison Gemmill, Özge Tunçalp, Ann-Beth Moller, Jane Daniels, A Metin Gülmezoglu, Marleen Temmerman, and Leontine Alkema. 2014. "Global Causes of Maternal Death: A WHO Systematic Analysis." *The Lancet Global Health* 2 (6): e323–33.

Stan Development Team. 2019. "Stan Modeling Language Users Guide and Reference Manual Version 2.25." https://mc-stan.org/.

.

APPENDIX 6: SUSTAINABLE DEVELOPMENT GOAL (SDG) REGIONAL CLASSIFICATION

Table S5 Sustainable Development Goal regional classification

SDG region	Sub regional grouping	Countries and territories
Australia and New Zealand	-	Australia, New Zealand
Central Asia and Southern Asia	Central Asia	Kazakhstan; Kyrgyzstan; Tajikistan; Turkmenistan; Uzbekistan
	Southern Asia	Afghanistan; Bangladesh; Bhutan; India; Iran (Islamic Republic of); Maldives; Nepal; Pakistan; Sri Lanka
Eastern Asia and South-eastern Asia	Eastern Asia	China; China, Hong Kong Special Administrative Region; China, Macao Special Administrative Region; Democratic People's Republic of Korea; Japan; Mongolia; Republic of Korea
	South-eastern Asia	Brunei Darussalam; Cambodia; Indonesia; Lao People's Democratic Republic; Malaysia; Myanmar; Philippines; Singapore; Thailand; Timor-Leste; Viet Nam
Latin America and the Caribbean	Caribbean	Anguilla; Antigua and Barbuda; Aruba; Bahamas; Barbados; Bonaire, Sint Eustatius and Saba; British Virgin Islands; Cayman Islands; Cuba; Curaçao; Dominica; Dominican Republic; Grenada; Guadeloupe; Haiti; Jamaica; Martinique; Montserrat; Puerto Rico' Saint Barthélemy; Saint Kitts and Nevis; Saint Lucia; Saint Vincent and the Grenadines; Sint Maarten (Dutch); Saint Martin (French); Trinidad and Tobago; Turks and Caicos Islands; United States Virgin Islands
	Central America	Belize; Honduras; Costa Rica; El Salvador; Guatemala; Mexico; Nicaragua; Panama
	South America	Argentina; Bolivia (Plurinational State of); Brazil; Chile; Colombia; Ecuador; French Guiana; Falkland Islands (Malvinas); South Georgia and the South Sandwich Islands; Guyana; Paraguay; Peru; Suriname; Uruguay; Venezuela (Bolivarian Republic of)
Northern America and Europe	Northern America	Bermuda; Canada; Greenland; United States of America
	Eastern Europe	Bulgaria; Belarus; Czechia; Hungary; Republic of Moldova; Poland; Romania; Russian Federation; Slovakia; Ukraine

	Northern Europe	Åland islands; Channel Islands; Denmark; Estonia; Faroe Islands; Finland; Isle of Man; United Kingdom of Great Britain and Northern Ireland; Iceland; Latvia; Lithuania; Norway; Svalbard and Jan Mayen Islands; Sweden
	Southern Europe	Albania; Andorra; Bosnia and Herzegovina; Croatia; Gibraltar; Greece; Holy See; Italy; Malta; Montenegro; North Macedonia; Portugal; San Marino; Serbia; Slovenia; Spain
	Western Europe	Austria; Belgium; Switzerland; Germany; France; Liechtenstein; Luxembourg; Monaco; Netherlands
Oceania / Oceania excluding Australia and New Zealand	-	Melanesia; Micronesia; Polynesia
Sub-Saharan Africa	-	Angola; Benin; Botswana; Burkina Faso; Burundi; Cabo Verde; Cameroon; Central African Republic; Chad; Comoros; Congo; Côte d'Ivoire; Democratic Republic of the Congo; Djibouti; Equatorial Guinea; Eritrea; Eswatini; Ethiopia; Gabon; Gambia; Ghana; Guinea; Guinea-Bissau; Kenya; Lesotho; Liberia; Madagascar; Malawi; Mali; Mauritania; Mauritius; Mayotte; Mozambique; Namibia; Niger; Nigeria; Réunion; Rwanda; Sao Tome and Principe; Senegal; Seychelles; Sierra Leone; Somalia; South Africa; South Sudan; Togo; Uganda; United Republic of Tanzania; Zambia; Zimbabwe
Western Asia and Northern Africa	Western Asia	Azerbaijan; Armenia; Bahrain; Cyprus; Georgia; Iraq; Israel; Jordan; Kuwait; Lebanon; State of Palestine; Oman; Qatar; Saudi Arabia; Syrian Arab Republic; Türkiye; United Arab Emirates; Yemen
	Northern Africa	Algeria; Egypt; Libya; Morocco; Sudan; Tunisia; Western Sahara

APPENDIX 7: Countries with and without observed data

Observed data were available for 129 countries, which were used to generate estimates for 185 countries (out of 194 WHO Member States, one Associate Member and one Territory). Table S6 shows the 56 countries without input data but with cause of maternal death estimates, 110 countries with CRVS data, 28 countries with Government reports and 20 countries with bibliographic study data

Table S6 Observed data availability by Sustainable Development Goal region

Data source type	SDG region	Countries and territories
No observed data available	Australia and New Zealand	N/A
	Central Asia and Southern Asia	Bhutan<br>Nepal<br>Pakistan<br>Turkmenistan
	Eastern Asia and South-eastern Asia	Cambodia<br>Democratic People's Republic of Korea<br>Indonesia<br>Kiribati<br>Lao People's Democratic Republic<br>Micronesia (Federated States of)<br>Papua New Guinea<br>Samoa<br>Timor-Leste<br>Tonga<br>Vanuatu<br>Viet Nam
	Latin America and the Caribbean	Bolivia (Plurinational State of)<br>Haiti
	Northern America and Europe	Albania<br>Ukraine
	Oceania / Oceania excluding Australia and New Zealand	N/A
	Sub-Saharan Africa	Angola<br>Benin<br>Burundi<br>Cameroon<br>Central African Republic<br>Chad<br>Comoros<br>Congo<br>Côte d'Ivoire<br>Democratic Republic of the Congo<br>Djibouti<br>Equatorial Guinea<br>Eritrea<br>Eswatini<br>Gabon<br>Gambia

		Guinea<br>Lesotho<br>Liberia<br>Madagascar<br>Mali<br>Mauritania<br>Namibia<br>Niger<br>São Tomé and Príncipe<br>Senegal<br>Seychelles<br>Somalia<br>South Sudan<br>Sudan<br>Togo<br>Uganda<br>United Republic of Tanzania
	Western Asia and Northern Africa	Algeria<br>Azerbaijan<br>Yemen
Civil Registration and Vital Statistics	Australia and New Zealand	Australia, New Zealand
	Central Asia and Southern Asia	Iran (Islamic Republic of), Kazakhstan, Kyrgyzstan, Sri Lanka, Maldives, Tajikistan, Uzbekistan
	Eastern Asia and South-eastern Asia	Brunei Darussalam, Japan, Republic of Korea, Mongolia, Malaysia, Philippines, Singapore, Thailand
	Latin America & the Caribbean	Argentina, Antigua and Barbuda, Bahamas, Belize, Brazil, Barbados, Chile, Colombia, Costa Rica, Cuba, Dominican Republic, Ecuador, Grenada, Guatemala, Guyana, Honduras, Jamaica, Saint Lucia, Mexico, Nicaragua, Panama, Peru, Puerto Rico, Paraguay, El Salvador, Suriname, Trinidad and Tobago, Uruguay, Saint Vincent and the Grenadines, Venezuela (Bolivarian Republic of)
	Northern America and Europe	Austria, Belgium, Bulgaria, Bosnia and Herzegovina, Belarus, Canada, Switzerland, Czechia, Germany, Denmark, Spain, Estonia, Finland, France, United Kingdom, Greece, Croatia, Hungary, Ireland,

		Iceland, Italy, Lithuania, Luxembourg, Latvia, Republic of Moldova, The former Yugoslav Republic of Macedonia, Malta, Montenegro, Netherlands, Norway, Poland, Portugal, Romania, Serbia, Slovakia, Slovenia, Sweden, United States of America
	Oceania excluding Australia and New Zealand	Fiji, Solomon Islands
	Sub-Saharan Africa	Cabo Verde, Mauritius, South Africa
	Western Asia and Northern Africa	United Arab Emirates, Armenia, Bahrain, Cyprus, Egypt, Georgia, Iraq, Israel, Jordan, Kuwait, Lebanon, Libya, Morocco, Oman, Occupied Palestinian Territory, Qatar, Saudi Arabia, Syrian Arab Republic, Tunisia, Turkey
Government reports	Australia and New Zealand	Australia, New Zealand
	Central Asia and Southern Asia	Afghanistan, Bangladesh, Sri Lanka, Kazakhstan, Kyrgyzstan
	Eastern Asia and South-eastern Asia	China, Mongolia
	Latin America & the Caribbean	Dominican Republic, Guatemala, Honduras, Paraguay
	Northern America and Europe	France, Ireland, United Kingdom, United States of America
	Oceania excluding Australia and New Zealand	Fiji
	Sub-Saharan Africa	Botswana, Ghana, Kenya, Mauritius, South Africa, Burkina Faso
	Western Asia and Northern Africa	Georgia, Iraq, Morocco, Jordan
Bibliographic database	Australia and New Zealand	NA
	Central Asia and Southern Asia	India, Iran (Islamic Republic of)
	Eastern Asia and South-eastern Asia	China, Myanmar
	Latin America & the Caribbean	Suriname, Jamaica
	Northern America and Europe	Russian Federation

	Oceania excluding Australia and New Zealand	NA
	Sub-Saharan Africa	Kenya, Nigeria, Malawi, Sierra Leone, Ghana, Rwanda, Zimbabwe, Zambia, Mozambique, Ethiopia, Guinea-Bissau
	Western Asia and Northern Africa	Iraq, Lebanon

APPENDIX 8: GLOBAL DISTRIBUTION OF MATERNAL DEATHS BY SDG REGION

TABLE S7: GLOBAL DISTRIBUTION OF MATERNAL DEATHS BY SDG REGION

7 Table S7 Globa Distribution of Maternal Deaths by SDG Region

SDG region[1]	ABO[2]		HYP		HEM		SEP		EMB		DIR		IND	
	Est. Count[3]	Est % (80% UI)[4]	Est. Count	Est % (80% UI)	Est. Count	Est % (80% UI)	Est. Count	Est % (80% UI)	Est. Count	Est % (80% UI)	Est. Count	Est % (80% UI)	Est. Count	Est % (80% UI)
Global	324926	8.4 (6.7, 10.9)	620754	16.1 (13.8, 18.8)	1028470	26.7 (22.2, 31.7)	252972	6.6 (5.2, 8.7)	283622	7.4 (5.5, 10.1)	379002	9.8 (7.8, 12.5)	886765	23.0 (18.0, 30.3)
Australia and New Zealand	26	10.2 (7.9, 13.0)	24	9.5 (7.6, 11.6)	37	14.9 (12.5, 17.7)	16	6.5 (5.0, 8.3)	49	19.8 (16.9, 22.9)	33	13.1 (10.6, 15.9)	63	25.3 (22.3, 28.5)
Central Asia and Southern Asia	79328	9.8 (6.1, 15.6)	126983	15.6 (11.2, 21.0)	194770	24.0 (16.8, 32.6)	42348	5.2 (3.4, 8.0)	75499	9.3 (5.5, 15.5)	76707	9.4 (6.0, 14.6)	181630	22.4 (13.0, 36.5)
Eastern Asia and South-eastern Asia	24985	9.3 (6.5, 13.6)	47824	17.7 (14.3, 22.2)	57296	21.3 (17.0, 26.6)	11939	4.4 (3.1, 6.4)	30800	11.4 (8.5, 16.1)	29856	11.1 (8.1, 15.4)	57896	21.5 (14.6, 32.5)
Latin America and the Caribbean	9428	9.7 (8.7, 11.2)	21212	21.9 (20.6, 23.4)	16435	17.0 (16.0, 18.2)	6535	6.8 (6.2, 7.5)	4836	5.0 (4.3, 6.1)	10735	11.1 (9.9, 12.7)	26769	27.6 (25.2, 31.0)
Northern America and Europe	1253	7.6 (6.7, 8.8)	2017	12.2 (11.2, 13.4)	2394	14.5 (13.0, 16.2)	901	5.4 (4.9, 6.1)	2809	17.0 (15.4, 19.0)	2470	14.9 (13.4, 16.6)	4590	27.7 (25.6, 30.6)
Oceania / Oceania excluding Australia and New Zealand	721	9.9 (5.0, 18.2)	1221	16.7 (10.1, 25.6)	1287	17.6 (9.9, 28.0)	377	5.2 (2.9, 9.1)	720	9.9 (5.1, 18.4)	749	10.3 (5.2, 18.6)	1633	22.4 (9.8, 45.6)

Sub-Saharan Africa	193902	7.7 (5.6, 11.0)	397575	15.7 (12.8, 19.4)	717502	28.4 (22.2, 35.5)	178272	7.1 (5.2, 10.4)	149096	5.9 (3.7, 9.5)	237864	9.4 (6.9, 13.1)	571129	22.6 (16.4, 32.6)
Western Asia and Northern Africa	7937	6.5 (4.4, 9.9)	19618	16.0 (12.7, 20.1)	35075	28.5 (22.4, 35.2)	7914	6.4 (4.8, 9.0)	10830	8.8 (6.5, 12.1)	13178	10.7 (7.9, 14.8)	24648	20.1 (13.3, 30.8)

[1] Sustainable Development Goal regional groupings are available in Appendix 5 and at: https://unstats.un.org/sdgs/indicators/regional-groups/
[2] Maternal causes of death are abbreviated as follows: ABO = abortion-related; HYP = hypertensive disorders of pregnancy; HEM = obstetric haemorrhage; SEP = sepsis; EMB = embolism; DIR = other direct causes; IND = indirect causes.
[3] Estimated count of deaths
[4] Estimated proportion with 80% uncertainty interval

APPENDIX 9: MATERNAL DEATHS DUE TO SUICIDE

8 Table S8 Maternal Deaths due to Suicide

SDG Region[1]	No. countries contributing data	Countries contributing data	Average no. of suicide maternal deaths reported	Average proportion of total reported maternal deaths due to suicide
Australia and New Zealand	2	AUS, NZL	4	0.26
Central Asia and Southern Asia	2	LKA, IND	4	0.03
Eastern Asia and South-eastern Asia	1	CHN	7	0.21
Latin America and the Caribbean	1	SUR	5	0.09
Northern America and Europe	3	FRA, IRL, GBR	11	0.06
Sub-Saharan Africa	1	MOZ	1	0
Western Asia and Northern Africa	2	GEO, JOR	2	0.04

[1] Sustainable Development Goal regional groupings are available in Appendix 5 and at: https://unstats.un.org/sdgs/indicators/regional-groups/
